# The role of omega-3 fatty acids in preventing glucocorticoid-induced reduction in human hippocampal neurogenesis and increase in apoptosis

**DOI:** 10.1038/s41398-020-00908-0

**Published:** 2020-07-07

**Authors:** Alessandra Borsini, Doris Stangl, Aaron R. Jeffries, Carmine M. Pariante, Sandrine Thuret

**Affiliations:** 1 Section of Stress, Psychiatry and Immunology & Perinatal Psychiatry, King’s College London, Institute of Psychiatry, Psychology & Neuroscience, Department of Psychological Medicine, London, UK; 2 King’s College London, Institute of Psychiatry, Psychology & Neuroscience, Department of Basic and Clinical Neuroscience, London, UK; 3 Biosciences, University of Exeter, Exeter, UK; 4 Department of Neurology, University Hospital Carl Gustav Carus, Technische Universität Dresden, Dresden, Germany

**Keywords:** Molecular neuroscience, Depression

## Abstract

Glucocorticoids have been suggested to be involved in several neuropsychiatric disorders, including depression. One of the possible mechanisms through which glucocorticoids contribute to the development of the depressive symptomatology is via regulation of distinct neurogenic mechanisms in the brain. A preventive or protective approach for these patients might be the use of omega-3 polyunsaturated fatty acids (n-3 PUFAs), eicosapentaenoic acid (EPA) and docosahexaenoic acid (DHA), which are known for they neuroprotective properties. We used the human hippocampal progenitor cell line HPC0A07/03C and pre-treated cells with either EPA or DHA, followed by treatment with the glucocorticoid cortisol either alone, or in co-treatment with the same n-3 PUFA during subsequent 3 days of proliferation and 7 days of differentiation. During proliferation, both EPA and DHA were able to prevent cortisol-induced reduction in proliferation and increase in apoptosis, when used in pre-treatment, and both pre- and co-treatment. During differentiation, EPA was able to prevent cortisol-induced reduction in neurogenesis and increase in apoptosis, when used in pre-treatment, and both pre- and co-treatment only during the proliferation stage; however, DHA required continuous treatment also during the differentiation stage to prevent cortisol-induced reduction in neurogenesis. Using transcriptomic analyses, we showed that both EPA and DHA regulated pathways involved in oxidative stress and immune response [e.g., nuclear factor (erythroid-derived 2)-like 2 (Nrf2), Signal transducer and activator of transcription 3 (STAT3), Interferon (IFN) and Interleukin (IL)-1 signaling], whereas DHA also regulated pathways involved in cell development and neuronal formation [e.g., cAMP-response element binding protein (CREB) signaling]. We provide the first evidence for treatment with both EPA and DHA to prevent cortisol-induced reduction in human hippocampal neurogenesis, and identify novel molecular mechanisms underlying these effects.

## Introduction

Glucocorticoids have been suggested to be involved in neurological disorders associated with stress, including neuropsychiatric disorders, such as depression^1–4^. The level of glucocorticoids in blood is increased in response to environmental stressors and are regulated by the negative feedback loop of the hypothalamic-pituitary-adrenal (HPA) axis^5,6^. Once these steroidal hormones are produced, they can act on a variety of biological and molecular systems in the brain, including neurogenesis^7^. In particular, findings from our group and other researchers, have shown that the endogenous glucocorticoid hormone cortisol, used at physiological concentration, can detrimentally affect cell proliferation and inhibit early hippocampal progenitor cells differentiation into neurons^7–11^. Furthermore, impaired neurogenesis in the hippocampal region may disrupt the HPA-axis functions and ultimately contribute to the onset of the brain diseases mentioned above^12^.

Although alteration in the stress response and in glucocorticoid production have often been associated with the depressive psychopathology, there are still no effective therapeutic approaches for these sub-group of patients. While attempts have been made to identify potential inhibitors of glucocorticoid-related molecules and signaling pathways, no satisfactory advancement has been produced so far^13^. More recently, increasing attention has been given to distinct nutritional components, contained in every day diet and named omega-3 polyunsaturated fatty acids (n-3 PUFAs), eicosapentaenoic acid (EPA) and docosahexaenoic acid (DHA), which have been known to reduce depressive symptoms in both patients^14–17^ and animal models^18^. Although the exact mechanisms underlying their mode of action remain unknown, n-3 PUFAs are important in regulating immune and oxidative stress responses in the brain, by inhibiting activation of pro-inflammatory cytokines and inducing the production of antioxidant molecules^19^. In turns, this would contribute to a sustained cell proliferation and neurogenesis^20^.

However, whether n-3 PUFAs can exert regulatory actions also on the glucocorticoid stress system it is still to be fully understood. The majority of studies published so far have focused on the effect of n-3 PUFAs when mainly used in co-treatment with glucocorticoids^21^, therefore preventing any understanding on whether n-3 PUFAs can also exert a preventive role in the development of stress-related neurogenic changes in the brain. Only one demonstrated the ability of pre-treatment with EPA and DHA to reduce corticosteroid production and depressive-like behaviors in stressed animals^22^, however, these clinical and behavioral changes were only correlated with peripheral biological markers of stress, with no clear indication of the effects of n-3 PUFAs in the CNS and particularly on neurogenesis. Therefore, to address this issue, we used our established in vitro model of human neurogenesis^7,20,23–27^ and exposed our immortalized human hippocampal progenitor cell line HPC0A07/03C to pre-treatment with either EPA or DHA, followed by treatment with cortisol alone, or in co-treatment with the same n-3 PUFAs. Finally, using a transcriptome analysis, we were able to investigate both unique and common signaling pathways putatively underlying the effect of EPA and DHA on hippocampal neurogenesis and apoptosis and relevant to cell development, cell death, inflammation, and oxidative stress.

## Methodology

### Cell culture

We used our established in vitro model of human hippocampal neurogenesis, the multipotent human hippocampal progenitor cell line HPC0A07/03C (provided by ReNeuron, Surrey, UK)^7,8,20,28–30^, which were recently tested for mycoplasma contamination. This model has been previously validated using a hippocampal newborn neuron specific marker, Prospero homeobox protein 1 (Prox1)^7,27^. Cells were left to proliferate in Dulbecco’s Modified Eagle Medium: Nutrient Mixture F-12 (DMEM/F-12) media to which we added the growth factors epidermal growth factor (EGF), basic fibroblast growth factor (bFGF) and 4-hydroxytamoxifen (4-OHT). Differentiation was initiated by removal of the growth factors and 4-OHT. Detailed information on this cell line can be found in our previous publications.

### Proliferation and differentiation assays

**Fig. 1:**
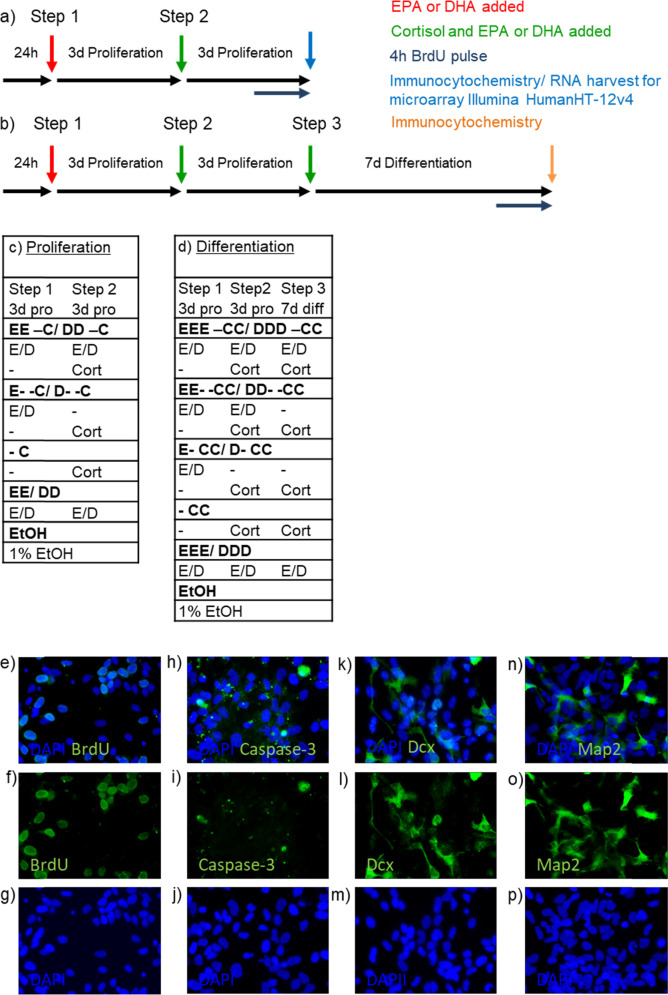
Experimental timeline and representative immunostaining images of HPC0A07/03C cells. **a**, **c** Cells were pre‑treated with EtOH, EPA or DHA for 3 days of proliferation, then EtOH, EPA or DHA, cortisol, or cortisol in co-incubation with EPA or DHA was added for the following 3 days of proliferation. **b**, **d** Differentiation was started, EtOH, EPA or DHA, cortisol, or cortisol with EPA or DHA was added for additional 7 days of differentiation until cells were fixed. **e**–**g** Immunocytochemistry for BrdU incorporation was used to assess progenitor cell proliferation over the total number of cells (DAPI). **h**–**j** Activated Caspase-3 was used to assed apoptosis, whereas **k**–**p** Dcx and MAP2 were used to assess respectively neuronal differentiation and maturation over the total number of cells (DAPI). (Scale bar: 20 µm). EtOH: ethanol; E: EPA; D: DHA; Cort: cortisol; Pro: proliferation; Diff: differentiation; BrdU: 5′-bromodeoxyuridine; DAPI: 4′,6-diamidino-2-phenylindole; Dcx: doublecortin; MAP2: microtubule associated protein-2.

To assess changes in cell proliferation, cells were grown on 96-well plates (Nunclon, Roskilde, Denmark) at an initial density of 1.1 × 10^4^ cells per well. After 24 h, media was changed and cells were pre-treated for 3 days with free form EPA (10 µM) or DHA (10 µM) dissolved in 100% EtOH (see Fig. 1a, Step 1). These concentrations were previously validated and used in all our studies with n-3 PUFA^20,30^, and resemble those found in brain of healthy individuals^31,32^. After 3 days of treatment media was changed and cells were then treated with cortisol alone (100 µM), n-3 PUFA (EPA or DHA) alone, or by co-treatment with cortisol and n-3 PUFA (EPA or DHA) for additional 3 days during proliferation (see Fig. 1a, Step 2). After a total of 6 treatment days, cultures were fixed with 4% paraformaldehyde (PFA) for 15 min at room temperature (RT). The synthetic nucleotide 5′-bromodeoxyuridine (BrdU, 10 μM) was added to the culture media 4 h before cell fixation. To assess changes in neuronal differentiation, cells were plated and grown as described above; after the 6 days of proliferation cultures were washed and cultured in media without growth factors and 4-OHT for subsequent 7 days with cortisol alone (100 µM), n-3 PUFA (EPA or DHA) alone, or in co-treatment with cortisol and the same n-3 PUFA (EPA or DHA) used during the proliferation stage (see Fig. 1b, Step 3). Further details on the experimental set up can be found in Fig. 1c, d. At the end of the total incubation time of 13 days, cells were fixed as described above.

### Immunocytochemistry

During the proliferation stage, cultures were stained for the proliferation marker and BrdU (1:500, Serotec, Oxford, UK, catalog number (cat.n.): BT0030) and the apoptotic marker Caspase-3 (1:500, Cell signalling Technology, Inc., cat.no.: 9661) (Fig. 1a, b). BrdU-containing cells were incubated with hydrochloric acid (HCl 2) for 15 min at RT, blocking solution for 60 min at RT, primary antibody (as above) at 4 °C overnight and secondary antibody (1:500, Alexa 488 donkey anti-rat, Invitrogen, cat.no.: ab150153) for 2 h at RT. In the differentiation experiment cells were stained for the apoptotic marker Caspase-3 (as above), the neuroblast marker Doublecortin (Dcx, 1:1000) and the mature neuronal marker microtubule-associated protein-2 (MAP2) (1:500, both Abcam, Cambridge, UK, cat.no.: ab18723, ab11267) (Fig. 1b). Briefly, PFA-fixed cells were incubated in blocking solution (5% normal donkey serum) in phosphate-buffered saline containing 0.1% Triton-X-100, for 1 h at RT, and with primary antibodies at 4 °C overnight. Cells were incubated sequentially in blocking solution for 30 min, secondary antibodies (Alexa 594 donkey anti-rabbit, Alexa 594 donkey anti-mouse, Alexa 488 donkey anti-rabbit, all 1:500, Invitrogen, Paisley, UK, cat.no.: ab150076, ab150108, ab150073) for 2 h and PBS containing 300 nM 4’,6 diamidino-2-phenylindole (DAPI) for 2 min at RT. The number of BrdU, Dcx, MAP2, and Caspase-3 positive cells over total DAPI positive cells was counted in an unbiased setup with an inverted microscope (IX70, Olympus, Hamburg, Germany) and ImageJ 4.41 software (http://rsbweb.nih.gov). Negative controls were incubated with the secondary antibody only. See Fig. 1e–p for representative images.

### RNA isolation and microarray analysis

Cells were cultured as described earlier and harvested for RNA extraction using RNeasy Mini Kit (Qiagen, Crawley, UK) (Fig. 1a). RNA was isolated using the RNeasy Micro Kit (Qiagen) following the manufacturer’s instructions, and samples were kept frozen at −80 °C until further use. RNA quantity and quality were assessed by evaluation of the A260/280 and A260/230 ratios using a Nanodrop spectrometer (NanoDrop Technologies). Samples were assayed on Illumina HumanHT-12 v4 Expression BeadChip Kit (Illumina, San Diego, CA, www.illumina.com) by BART Genome Centre, The Sir John Vane Science Centre. The raw microarray data were processed using the R based lumi Bioconductor package^33^ by applying variance stabilizing transformation followed by robust spline normalization. Lists of significant differentially expressed genes were then identified using Significance analysis of microarrays^34^ with a *p* value of *p* < 0.05 and corrected for false discovery rate (FDR).

### Pathway and network analysis

Ingenuity Pathway Analyses Software (IPA) was used to identify the regulation of molecular signaling pathways with significance threshold of a log value equal to 1.3 (*p* < 0.05). As a background, we used gene lists that we obtained applying the minimum absolute fold change cut-off of 1.2 and *p*-value < 0.05. Genes that passed these criteria were used to build up the Venn diagram. Within each sub-group of genes identified by the Venn diagram, a protein-protein interaction network with Markov Clustering (MCL) Algorithm was constructed using the Search Tool for the Retrieval of Interacting Genes/Proteins (STRING) Network Analyses software (http://string-db.org/), in order to identify the closest interacting genes and the main networks that are regulated (Figs. S1a–h and S2a–g).

### Statistical analysis

Each experiment was replicated in at least six independent cultures (biological replicates) with 12 technical replicates per condition in order to have >80% power, at *p* = 0.05 and an effect size differences of 0.6, that is the same effect size described when comparing the effects of treatment with cortisol vs. vehicle on neuronal differentiation^35^. All statistical analyses were performed with GraphPad Prism 6.00. Two-Way ANOVA with Bonferroni’s post hoc test was used for multiple comparisons among treatment groups. Wilcoxon’s signed rank test was used to compare means of two independent treatment groups. Variance was similar between the groups that have been statistically compared. Results were regarded as significant with a *p* value of *p* < 0.05.

## Results

### Pre-treatment with EPA prevents cortisol-induced decrease in cell proliferation and increase in apoptosis

**Fig. 2:**
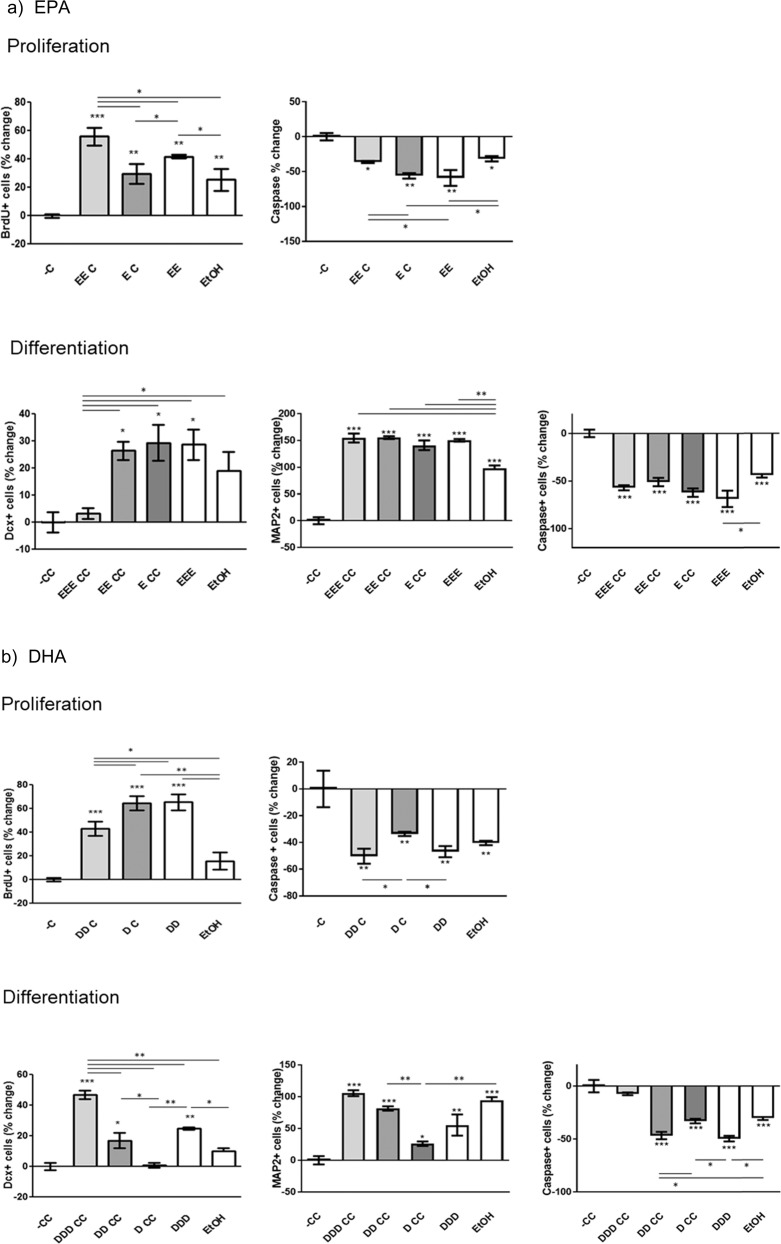
The effect of treatment with EPA and DHA in preventing cortisol-induced reduction in cell proliferation, neurogenesis, and increase in apoptosis. **a** During proliferation, pre-treatment with EPA followed by cortisol alone (EC) or by co-treatment with EPA and cortisol (EEC) prevent the decrease in proliferation (BrdU+ cells) and the increase in apoptosis (Caspase-3+ cells) caused by cortisol (−C). During differentiation, pre-treatment with EPA followed by cortisol either alone (ECC), or by co-treatment with EPA (EECC) during the proliferation stage prevented the reduction in DCX+ and Map2+ cells, and the increase in Caspase-3+cells caused by cortisol (−CC). Similar effects were found when cells were co-treated with EPA also during the differentiation stage (EEECC) for Map2+ and Caspase-3+ cells, but not for DCX. **b** During proliferation, pre-treatment with DHA followed by cortisol alone (DC) or by co-treatment with DHA and cortisol (DDC) prevented the decrease in proliferation (BrdU+ cells) and the increase in apoptosis (Caspase-3+ cells) caused by cortisol (−CC). During differentiation, pre-treatment with DHA followed by cortisol alone (DCC) during the proliferation stage did not prevent the reduction in DCX+ and Map2+ cells caused by cortisol (−CC), but prevented the increase in Caspase-3+ cells. Pre-treatment with DHA followed by cortisol in co-treatment with DHA (DDCC) prevented the reduction in DCX+ and Map2+ cells, and the increase in Caspase-3+ cells caused by cortisol (−CC). Similar effects were found when cells were co-treated with DHA also during the differentiation stage (DDDCC) for DCX+ and Map2+ cells, but not for Caspase-3. Data are shown as mean±standard error of the mean, *p<0.05, **p<0.01, ***p<0.001 compared with cortisol treatment (−C or −CC), unless otherwise indicated in the figure.

Cells were pre-treated with EPA (10 µM) for 3 days of proliferation followed by 3 days with either EPA alone (10 µM), cortisol alone (100 µM) or with cortisol and EPA in co-incubation. We found a significant reduction in the number of BrdU+ cells and an increase in Caspase 3+ cells upon treatment with cortisol alone (**−C**) when compared with control condition (**EtOH**) (−25 and +33%, respectively; Fig. 2a). In contrast, treatment with EPA alone (**EE**) increased the number of BrdU+ cells and decreased the number of Caspase 3+ cells when compared with control (+15 and −25%, respectively; Fig. 2a).

Moreover, pre-treatment with EPA followed by cortisol either alone (**EC**) or in co-treatment with EPA (**EEC**) prevented the reduction in BrdU+ cells originally caused by cortisol (**-C**) (from −25 to +5 and +33%, respectively; Fig. 2a). Interestingly, the effect of pre- and co-treatment with EPA in the presence of cortisol (**EEC**) was more effective than EPA alone (**EE**) (+33 vs +15%). Similarly, pre-treatment with EPA followed by cortisol either alone (**EC**) or in co-treatment with EPA (**EEC**) prevented the increase in Caspase 3+ cells originally caused by cortisol (**-C**) (from+ 33 to −24 and −2%, respectively; Fig. 2a). In this case, however the effect of **EC**, but not **EEC** was as strong as EPA alone (**EE**) (−24 vs −25%).

### Pre-treatment with EPA prevents cortisol-induced decrease in neurogenesis and increase in apoptosis

After 6 days of proliferation, cells were let to differentiate for subsequent 7 days with either EPA alone (10 µM), cortisol alone (100 µM) or with cortisol and EPA in co-incubation. We found a significant reduction in the number of DCX+ cells and Map2+ cells, and an increase in Caspase 3+ cells upon treatment with cortisol alone (−**CC**) when compared with control condition (**EtOH**) (−19, −99, and +37%, respectively; Fig. 2a). In contrast, treatment with EPA alone (**EEE**) increased the number of DCX+ cells and Map2+ cells and decreased the number of Caspase 3+ cells when compared with control (+10, +51, and −29%, respectively; Fig. 2a).

Moreover, pre-treatment with EPA during the proliferation stage followed by cortisol either alone (**ECC**) or in co-incubation with EPA (**EECC**) prevented the reduction in DCX+ (from −19 to +10 and +8%, respectively; Fig. 2a), Map2+ cells (from −99 to +49 and +51%, respectively; Fig. 2a), and the increase in Caspase 3+ cells caused by cortisol (**−CC**) (from+ 37 to −25 and −13%, respectively; Fig. 2a). Similar effects were found for Map2+ and Caspase 3+ cells (from −99 to +51%; and from +37 to −13%, respectively; Fig. 2a), but not for DCX (from −19 to −16%; Fig. 2a) when cells were co-treated with EPA also during the differentiation stage (**EEECC**).

### Pre-treatment with DHA prevents cortisol-induced decrease in cell proliferation and increase in apoptosis

Similar to the experiments with EPA, cells were pre-treated with DHA (10 µM) for 3 days of proliferation followed by 3 days with either DHA alone (10 µM), cortisol alone (100 µM) or with cortisol and DHA in co-incubation. There was a significant reduction in the number of BrdU+ cells and an increase in Caspase 3+ cells upon treatment with cortisol alone (−**C**) when compared with control condition (**EtOH**) (−18 and +41%, respectively; Fig. 2b). In contrast, treatment with DHA alone (**DD**) increased the number of BrdU+ cells and decreased the number of Caspase 3+ cells when compared with control (+47 and −4%, respectively; Fig. 2b).

Moreover, pre-treatment with DHA followed by cortisol either alone (**DC**) or in co-treatment with DHA (**DDC**) prevented the reduction in BrdU+ cells originally caused by cortisol (−**C**) (from −18 to +47 and +24%, respectively; Fig. 2b). Interestingly, the effect of **DC**, but not **DDC** was equal to DHA alone (**DD**) (+47 vs +46%). Similarly, pre-treatment with DHA followed by cortisol either alone (**DC**) or in co-treatment with DHA (**DDC**) prevented the increase in Caspase 3+ cells originally caused by cortisol (−**C**) (from+ 41 to +3 and −9%, respectively; Fig. 2b). In this case, however, the effect of **DDC**, but not **DC**, was as strong as DHA alone (**DD**) (−9 vs −8%).

### Pre-treatment with DHA prevents cortisol-induced decrease in neurogenesis and increase in apoptosis

Similar to the experiments with DHA, after 6 days of proliferation cells were let to differentiate for subsequent 7 days with either DHA alone (10 µM), cortisol alone (100 µM) or with cortisol and DHA in co-incubation. We found a significant reduction in the number of DCX+ cells and Map2+ cells, and an increase in Caspase 3+ cells upon treatment with cortisol alone (−**CC**) when compared with control condition (**EtOH**) (−10, −90 and +30%, respectively; Fig. 2b). In contrast, treatment with DHA alone (**DDD**) increased the number of DCX+ cells and Map2+ cells and decreased the number of Caspase 3+ cells when compared with control (+15, +48 and −20% respectively; Fig. 2b).

Pre-treatment with DHA during the proliferation stage followed by cortisol alone (**DCC**) did not prevent the reduction in DCX+ and Map2+ cells caused by cortisol (−**CC**) (from −10 to −8%; and from −90 to −74%, respectively; Fig. 2b), but prevented the increase in Caspase 3+ cells (from+ 30 to −18%, Fig. 2b). Moreover, pre-treatment with DHA during the proliferation stage followed by cortisol in co-treatment with DHA (**DDCC**) prevented the reduction in DCX+ and Map2+ cells, and the increase in Caspase 3+ cells caused by cortisol (**−CC**) (from −10 to +5%, from −90 to −2%, and from +30 to −28%, respectively; Fig. 2b). Similar effects were found when cells were co-treated with DHA also during the differentiation stage (**DDDCC**) for DCX+ and Map2+ cells, but not for Caspase 3 (from −10 to +35%, from −90 to +10%, and from +30 to −28%, respectively; Fig. 2b).

### EPA prevents the detrimental effect of cortisol via modulating distinct signaling pathways involved in oxidative stress and inflammatory response

**Fig. 3:**
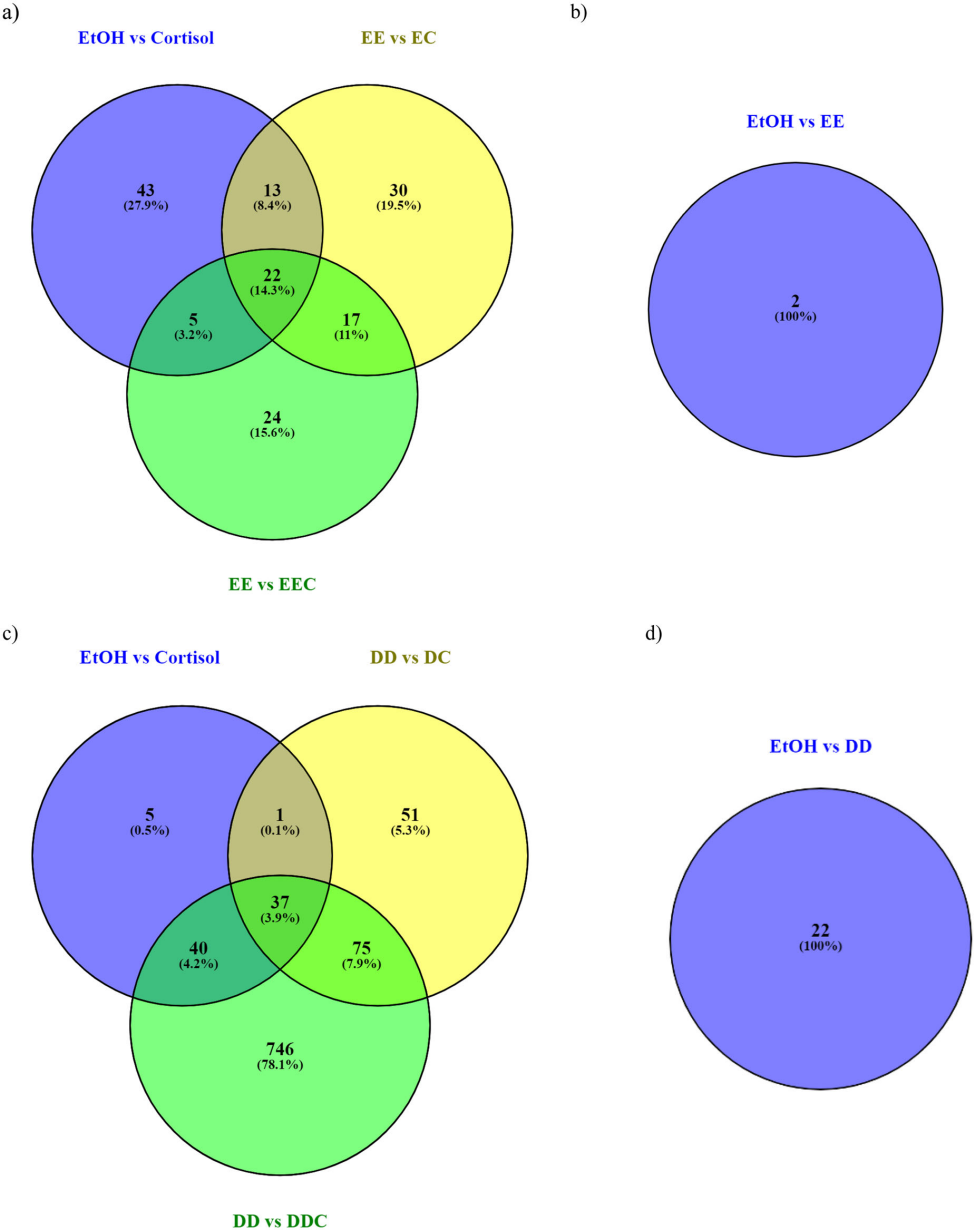
Venn diagram showing genes uniquely and commonly modulated between cortisol, EPA/DHA treatment alone, and EPA/DHA in pre-treatment, and both pre- and co-treatment with cortisol. **a** Genes modulated upon treatment with cortisol alone, EPA (**EE**) alone, or with EPA pre-treatment (**EC**) and EPA pre- and co-treatment with cortisol (**EEC**), **b** genes modulated by EPA alone (**EE**); **c** gene modulated upon treatment with cortisol alone, DHA (**DD**) alone, or with DHA pre-treatment (**DC**) and DHA pre- and co-treatment with cortisol (**DDC**), **d** genes modulated by DHA alone (**DD**).

To further identify the molecular mechanisms potentially involved in the effects of EPA in preventing cortisol-driven changes in neurogenesis and apoptosis, we decided to focus specifically on the signaling mechanisms modulated during the proliferation phase, as treatment during this phase was necessary and sufficient for cortisol to reduce neurogenesis, as described in our previous publication^7^, and for EPA and DHA to prevent such effects, as demonstrated above. Therefore, we analyzed gene expression changes by transcriptomics at the end of the 6 days of the proliferation, as our previous immunocytochemistry experiments. Overall, cortisol (−**C**) modulated 83 genes (Fig. 3a), whereas EPA (**EE**) modulated only 2 genes when compared with control (see Supplementary Materials, Fig. 3b). Interestingly, when EPA was used as pre-treatment (**EC**), or both in pre- and co-treatment with cortisol (**EEC**) up to 72 genes and 68 genes were modulated when compared with **EE** alone (Fig. 3a). The complete gene expression analysis is presented in Table S1.

Considering that **EC** and **EEC** exerted similar effects, but with different intensity, in preventing cortisol-induced reduction in proliferation, neurogenesis and apoptosis, we have primarily focused on genes uniquely modulated by **EC** versus **EEC**, with respect to cortisol (−**C**) (Fig. 3a). Indeed, genes which were commonly shared among these conditions were all regulated towards the same direction (see Supplementary Materials), therefore making their analyses less relevant if we were to depict individual effects of **EC** and **EEC**.

A total of 43 genes were uniquely regulated by treatment with cortisol alone (Table S1, and Fig. S1a). Overall these genes modulated 32 pathways (Table S2). Among them of particular interest are “Axonal Guidance Signaling”, “Production of Nitric Oxide and Reactive Oxygen Species” and “mammalian target of rapamycin (mTOR) Signaling” pathways. Genes belonging to the first pathway, including tubulin beta (TUBB) and ADAM metallopeptidase domain 19 (meltrin beta) (ADAM19), have been involved in regulation of cell development, proliferation and neurogenesis^36,37^. Whereas among the genes belonging to the other two pathways there is the Rho family GTPase 3 (RND3) gene. Activation of the anti-inflammatory mTOR pathway is responsible for the increased expression of RND3, which has protective effects against oxidative stress^38^ and enhances proliferation and neurogenesis^39^. All the above genes were significantly downregulated by cortisol (Table S1).

In contrast, 30 genes were uniquely regulated by EPA when used only in pre-treatment (**EC**) (Table S1, and Fig. S1b). Overall these genes modulated 26 pathways (Table S2). Signaling pathways include, “C–X–C Motif Chemokine Receptor 4 (CXCR4) Signaling”, “nuclear factor (erythroid-derived 2)-like 2 (Nrf2) Oxidative Response Stress” and “Glucocorticoid Receptor Signaling”. One activator of the CXCR4 is the gene engulfment and cell motility 1 (ELMO1), which has been shown to increase cell apoptosis and mitochondrial fragmentation, and to reduce cell motility^40^. Whereas, DnaJ (Hsp40) homolog, subfamily B, member 6 (DNAJB6) gene is member of the Nrf-2 oxidative response pathway, and SWI/SNF related actin-dependent regulator of chromatin, subfamily b, member 1 (SMARCB1) gene belongs to the glucocorticoid receptor-dependent signaling pathway. Both genes are known to enhance cell apoptosis^41–43^ and were indeed downregulated, together with ELMO1, by pre-treatment with EPA (Table S1).

Finally, 24 genes were uniquely regulated by EPA when used both in pre-treatment and co-treatment (**EEC**) (Table S1, and Fig. S1c). Overall these genes modulated 16 pathways (Table S2). Signaling pathways involve, “Signal transducer and activator of transcription 3 (STAT3)” and “Nuclear Factor Kappa B Subunit 1 (NF-κB) Signaling” and “Mitotic Roles of Polo-Like Kinase”. The gene platelet-derived growth factor receptor, alpha polypeptide (PDGFRA) is a regulator of distinct inflammatory molecules, including the transcription factors STAT3 and NF-κB, all of which have been shown to mediate detrimental effects on cell proliferation and neurogenesis^44,45^. Whereas, the anaphase-promoting complex subunit 1 (ANAPC1) gene is involved in the polo-like protein kinase signaling pathway and regulates cell proliferation^46^. These genes were respectively down- and upregulated by pre- and co-treatment with EPA (Tables S1).

### DHA prevents the detrimental effect of cortisol via modulating distinct signaling pathways involved in cell development, cell death, oxidative stress, and inflammatory response

We also conducted gene expression and pathway analysis in cells pre-treated with DHA. Overall, DHA (**DD**) modulated 22 genes when compared with control (see Supplementary Materials, Fig. 3d), whereas when DHA was used as pre-treatment (**DC**), or both in pre- and co-treatment with cortisol (**DDC**) up to 164 and 898 genes were modulated when compared with **DD** (Fig. 3c, Table S3). As for EPA, we have primarily focused on genes uniquely regulated by cortisol (−**C**), **DC** and **DDC**, as genes commonly shared among these conditions were all regulated towards the same direction (see Supplementary Materials), and therefore less relevant if we were to depict individual effects of **DC** and **DDC** with respect to cortisol.

A total of 5 genes were uniquely regulated by cortisol (Table S3 and Fig. S2a). Overall, these genes modulated 3 pathways (Table S4). Among them of particular interest is the “cyclin dependent kinase (CDK)5 Signaling” and its gene protease, serine, 23 (PRSS23), which is known to regulate cell proliferation and cell development^47^ and which was indeed downregulated by cortisol (Table S3). In contrast, 51 genes were uniquely regulated by DHA when used only in pre-treatment (**DC**) (Table S3 and Fig. S2b). These genes modulated 12 pathways (Table S4). Among these pathways of relevance are “Sirtuin Signaling Pathway”, “fibroblast growth factor (FGF) Signaling” and “Interferon (IFN) Signaling”. The gene 6-phosphofructo-2-kinase/fructose-2,6-biphosphatase 3 (PFKFB3) and FGF Receptor 3 (FGFR3) respectively belong to the first and second pathway, and are known to positively regulate cell generation and proliferation^48,49^. Whereas the gene interferon induced transmembrane protein 3 (1-8U) (IFITM3) belongs to the last signaling pathway and it is involved in the innate inflammatory response and known to suppress cell proliferation^50^. Accordingly, the first two genes were upregulated, whereas IFITM3 was downregulated by DHA (Table S3).

Finally, 746 genes were uniquely regulated by DHA when used both in pre- and co-treatment (**DDC**) (Table S3 and Fig. S2c). Overall, these genes modulated 122 pathways (Table S4). Among those, the most relevant are “p53 Signaling”, “cAMP-response element-binding protein (CREB) Signaling in Neurons”, “Nrf2-mediated Oxidative Stress Response” and “Interleukin (IL)-1 Signaling”. The growth arrest and DNA-damage-inducible, gamma (GADD45G) gene belongs to the first pathway and is activated in response to environmental stresses by mediating further downstream mechanisms, like p38/Janus Kinase pathway, which induces cell apoptosis^51^. Phosphoinositide-3-Kinase Regulatory Subunit 3 (PIK3R3) acts as a second messengers in regulating growth signaling pathways, like CREB, and promotes cell development and neurogenesis^52^, whereas DnaJ (Hsp40) homolog, subfamily A, member 1 (DNAJA1) is involved in the oxidative stress response and detrimentally affects neurogenesis and increases apoptosis^53^. Finally, mitogen-activated protein kinase 14 (MAP3K14) mediates IL-1 signaling pathways, including activation of NF-κB, which is known to induce detrimental effects on neurogenesis^20,28^. The genes GADD45G, DNAJA1, and MAP3K14 were downregulated, whereas PIK3R3 was upregulated by DHA (Table S3).

## Discussion

In this study, we provide the first evidence for treatment with both EPA and DHA to prevent decrease in human hippocampal cell proliferation and neurogenesis, and increase in apoptosis caused by treatment with physiological concentrations of cortisol. In particular, during proliferation, both EPA and DHA were able to prevent cortisol-induced reduction in proliferation and increase in apoptosis, when used in pre-treatment, and both pre- and co-treatment. During differentiation, EPA was able to prevent cortisol-induced reduction in neurogenesis and increase in apoptosis, when used in pre-treatment, and both pre- and co-treatment only during the proliferation stage; however, DHA required continuous treatment also during the differentiation stage to prevent cortisol-induced reduction in neurogenesis. At the molecular level, we showed that both EPA and DHA regulate pathways involved in oxidative stress and immune response (e.g., Nrf2, STAT3, IFN, and IL-1 signaling), whereas DHA also regulated pathways involved in cell development and neuronal formation (e.g., CREB signaling).

With respect to proliferation, this study confirms our previous findings showing detrimental effects of cortisol on cell proliferation^7,8,29^, and moreover it highlights differential effects between EPA and DHA in preventing-cortisol-induced decrease in cell proliferation and increase in apoptosis, when used in pre-treatment, or both pre- and co-treatment with cortisol. In particular, while the effect on proliferation was stronger with EPA used in pre-and co-treatment (**EEC**) and DHA in pre-treatment (**DC**), the effect on apoptosis was stronger with EPA in pre-treatment (**EC**) and DHA in pre- and co-treatment (**DDC**). This is in line with previous evidence showing that both EPA and DHA have proliferative and anti-apoptotic properties^54–57^. However, such evidence are primarily generated from in vitro studies where n-3 PUFAs were used only in pre-treatment, and upon exposure to apoptotic stimuli, like neurotoxins, but not to stressful stimuli, like cortisol^54–57^. Therefore, our study is the first one to demonstrate that, in the context of stress, EPA and DHA can exert either proliferative or anti-apoptotic properties depending on the type of experimental assay in which they are employed, pre-treatment alone versus both pre- and co-treatment.

With respect to differentiation, this study confirms our previous findings showing detrimental effects of cortisol on neurogenesis^7,8,29^, and moreover, it highlights differential effects between EPA and DHA in preventing-cortisol-induced decrease in neurogenesis and increase in apoptosis. In particular, EPA was able to prevent cortisol-induced reduction in neurogenesis and increase in apoptosis, when used in pre-treatment, and both pre- and co-treatment only during the proliferation stage (**ECC** and **EECC**); however, DHA required continuous treatment also during the differentiation stage to prevent cortisol-induced reduction in neurogenesis (**DDDCC**). Therefore, this seems to suggest that although both EPA and DHA exert neuroprotective properties^20^, the effect of EPA (**ECC** and **EECC**) but not of DHA (**DCC**), during the proliferation stage is sufficient to prevent cortisol-induced decrease in neurogenesis later on. However, it is interesting to note that although EPA treatment increased the proportion of differentiating cells, this effect was never beyond the level of the EPA only treated cultures (**EEE**). DHA instead, when used during both the proliferation and differentiation stage (**DDDCC**), increased the number of Dcx+ and MAP2+ cells up to 20 and 60%, respectively, when compared with treatment with DHA alone (**DDD**), therefore confirming the ability for DHA to be more neuroprotective than EPA^58^. This also seems to suggest that DHA, similarly to antidepressants^59^, may become activated only upon exposure to chronic stressors, like in our experimental assay where cells were exposed to continuous cortisol treatment for over 10 days. However, aside from its neuroprotective properties, continuous treatment with DHA during both proliferation and differentiation (**DDDCC**) did not prevent the increase in apoptosis caused by cortisol, whereas EPA, when used in the same experimental assay (**EEECC**) did exert anti-apoptotic effects. Therefore, this demonstrates that although during the proliferation stage both EPA and DHA exert proliferative and anti-apoptotic properties, which are dependent on the type of experimental paradigm used (pre-treatment, versus both pre- and co-treatment), during the differentiation stage EPA and DHA have unique properties. While EPA prevents cortisol-induced cell apoptosis, DHA regulates cell fate and cell differentiation into neuroblasts and mature neurons, which was originally disrupted by cortisol.

Subsequently, we also investigated signaling pathways underlying the effects of cortisol on cell proliferation, differentiation and apoptosis; and the differential outcomes of EPA and DHA in preventing such effects. In contrast with our previous study, where we investigated gene expression changes after 12 h of cortisol treatment^7^, in the present study 3 days of treatment with cortisol downregulated genes involved in the axonal guidance and anti-inflammatory response, like the mTOR signalling, but it upregulated genes belonging to the Nrf-2 oxidative stress response. On the other hand, EPA and DHA modulated genes involved in similar pathways but on the opposite direction. While both EPA and DHA decrease the expression of genes involved in the oxidative stress (Nrf-2) and inflammatory response (STAT3, IFN, and IL-1 signaling), DHA only, upregulated genes belonging to the neuroprotective CREB signaling pathway. Indeed, these findings are in line with our data on neurogenesis, as well as with previous studies showing a more neuroprotective and neurogenic role for DHA, when compared with EPA^58^. In contrast, EPA seems to act more as an anti-inflammatory agent, able to inhibit pathways related to the innate immune response^20,60^, and through which it might exert its anti-apoptotic properties. Therefore, this makes EPA potentially more suitable for the treatment of neuroinflammatory conditions^61–70^, whereas DHA might be more effective for the treatment of neurodegenerative conditions^71^. This hypothesis is indeed supported by several lines of evidence, from epidemiological and case-controlled studies^72,73^, to randomized-controlled trials^74,75^, and meta-analyses^76,77^.

The immortalized cell line that we used in the study, while being of invaluable importance for our understanding of molecular mechanisms occurring in the hippocampal progenitor cells, may differ from the scenario of an adult in vivo environment and the adult neurogenic niche. However, all our previous results with this in vitro model have been replicated by animal or clinical studies, including changes in neurogenesis by cortisol, cytokines and antidepressants, and changes in stress-, inflammation- and antidepressants-regulated genes^7,8,20,23,24,28,29,60^. Therefore, we are confident that our results are relevant to the human brain. Of note, we did not assess the effect of cortisol in pre- and/or co-treatment with n-3 PUFAs on astrogliogenesis and oligodendrogenesis, as we decided to primarily focus on cell proliferation, differentiation and apoptosis. In our future studies, we aim to extend these findings and explore whether n-3 PUFAs can prevent glia-related alterations, which we already know can be induced by such stressful challenge^7^.

In summary, our study reveals the ability for both EPA and DHA to prevent cortisol-induced reduction in proliferation and neurogenesis and increase in apoptosis. While during proliferation both EPA and DHA exert similar proliferative and anti-apoptotic effects, during differentiation EPA act more effectively against apoptosis whereas DHA exerts more neurogenic properties. Such effects were putatively mediated by common signaling pathways involved in oxidative stress and inflammation (both EPA and DHA), and by unique signaling pathways involved in cell development and differentiation (DHA only). Overall, the presence of a cell population in the hippocampus that is highly sensitive to EPA and DHA, responding with a significant activation of n-3 PUFAs-induced genes, highlights the potential role of the hippocampus in the beneficial effects of these nutritional compounds as potential therapeutic strategies for patients with stress-related neuropsychiatric and neuroinflammatory conditions.

## Supplementary information

Supplementary Materials

Figure S1

Figure S2

Table S1

Table S2

Table S3

Table S4
