# Supplementary material for: The role of omega-3 fatty acids in preventing glucocorticoid-induced reduction in human hippocampal neurogenesis and increase in apoptosis
Source: Transl Psychiatry. 2020 Jul 7;10:219. doi: 10.1038/s41398-020-00908-0 (PMC7341841; doi:10.1038/s41398-020-00908-0)
Supplement: Supplementary file 1 — Supplementary Materials [file 41398_2020_908_MOESM1_ESM.docx]

*For submission to Translational Psychiatry – Supplementary Materials*

**The role of omega-3 fatty acids in preventing glucocorticoid-induced reduction in human hippocampal neurogenesis and increase in apoptosis**

Alessandra Borsini, PhD^1, 2*#^, Doris Stangl, PhD^2*^, Aaron R. Jeffries PhD^3^, Carmine M. Pariante, PhD^1^, Sandrine Thuret, PhD^2, 4#^.

1 Section of Stress, Psychiatry and Immunology & Perinatal Psychiatry, King’s College London, Institute of Psychiatry, Psychology & Neuroscience, Department of Psychological Medicine, London, UK.

2 King’s College London, Institute of Psychiatry, Psychology & Neuroscience, Department of Basic and Clinical Neuroscience, London, UK.

3 Biosciences, University of Exeter, Exeter, UK.

4 Department of Neurology, University Hospital Carl Gustav Carus, Technische Universität Dresden, Germany.

**These authors contributed equally to the manuscript*

# Corresponding Authors

Alessandra Borsini, PhD

Stress, Psychiatry and Immunology Lab & Perinatal Psychiatry

Institute of Psychiatry, Psychology and Neuroscience, King’s College London

G.32.01, The Maurice Wohl Clinical Neuroscience Institute

Cutcombe Road, London, SE5 9RT

Tel: 020 7848 0726; Email: [alessandra.borsini@kcl.ac.uk](mailto:alessandra.borsini@kcl.ac.uk)

Sandrine Thuret, PhD

Head of the Neurogenesis & Mental Health Laboratory

Reader in Neuroscience & Mental Health

King's College London, Institute of Psychiatry, Psychology & Neuroscience

Basic & Clinical Neuroscience Department, Maurice Wohl Clinical Neuroscience Institute

125 Coldharbour Lane, London, SE5 9NU

Tel: +44 (0) 20 7848 5405; Email: sandrine.1.thuret@kcl.ac.uk

**SUPPLEMENTARY RESULTS**

**EPA and DHA modulate distinct signalling pathways involved in cell proliferation and cell development**

A total of 2 genes were uniquely regulated by EPA (**EE**) when compared with control (**EtOH**) (Table S1, and Figure S1d). Overall, these genes modulated 2 pathways (Table S2). Of particular interest is the “peroxisome proliferator-activated receptors (PPAR) signaling pathway” with its gene angiopoietin-like 4 (ANGPTL4), an adipokine protein playing roles in lipid metabolism and in promoting cell proliferation^1^. Whereas, a total of 22 genes were uniquely regulated by DHA (**DD**) versus control (Table S3, and Figure S2d). These genes modulated 17 pathways (Table S4). Among these pathways of relevance is the “Mitochondrial Dysfunction” pathway with its gene carnitine palmitoyltransferase 1A (liver) (CPT1A), which is a key molecule involved in lipid metabolism and required for normal neurogenesis to occur ^2^. Both ANGPTL4 and CPT1A were upregulated respectively by EPA and DHA.

**Cortisol activates distinct compensatory signalling pathways involved in cell proliferation and cell development, which are similar to those regulated by EPA when used in pre-treatment, and both pre- and co-treatment with cortisol**

A total of 13 genes were commonly regulated by cortisol (**-C**) (vs control or **EtOH**) and EPA when used in pre-treatment (**EC**) (vs EPA alone, **EE**) (Table S1, and Figure S1e). Overall, these genes modulated 12 pathways (Table S2). Among them of particular interest is the “Growth Hormone Signaling” pathway with its gene insulin-like growth factor binding protein 3 (IGFBP3), which is known to increase cell apoptosis and to decrease neurogenesis^3, 4^. Furthermore, a total of 5 genes were regulated by both cortisol (vs control) and by pre- and co-treatment with EPA (**EEC**) (vs **EE**) (Table S1, and Figure S1f). Overall, these genes modulated 5 pathways (Table S2). Of relevance is the “PXR/RXR Activation” pathway with its gene ATP-binding cassette, sub-family C member 3 (ABCC3). PXR is a transcriptional regulator of the ATP transporter gene ABCC3, which promotes cell proliferation and neurogenesis ^5^. The pro-apoptotic IGFBP3 and the neurogenic ABCC3 were respectively downregulated and upregulated not only by EPA but also by cortisol. This perhaps suggests that treatment with cortisol induces the activation of distinct neuroprotective compensatory pathways, which are similar to those activated by pre-treatment, and pre- and co-treatment with EPA.

In addition, 17 genes were commonly regulated by EPA when used in pre-treatment (**EC**) (vs **EE**), and both pre- and co-treatment (**EEC**) (vs **EE**) (Table S1, and Figure S1g). Overall, these genes modulated 13 signalling pathways (Table S2). Among them, the “Protein Ubiquitination Pathway” with its gene neural precursor cell expressed, developmentally down-regulated 4-like (NEDD4L), is of particular interest. NEDD4L is involved in lysosomal degradation and inhibition of neurogenesis ^6^. This gene was indeed downregulated by pre-treatment, and pre- and co-treatment with EPA.

Finally, 22 gene were commonly regulated by cortisol (**-C**) (vs control), and EPA used in pre-treatment (**EC**) (vs **EE**) and both pre- and co-treatment with cortisol (**EEC**) (vs **EE**) (Table S1, and Figure S1h). Overall, these genes modulated 13 pathways (Table S2). Of relevance is the “Cyclins and Cell Cycle Regulation” pathway with its gene cyclin D3 (CCND3). CCND3 promotes cell proliferation and neuronal differentiation^7^. This gene was upregulated by both cortisol and EPA, again suggesting that treatment with cortisol is able to activate distinct neuroprotective compensatory pathway similar to those activated by pre-treatment, and both pre- and co-treatment with EPA.

**Cortisol activates distinct compensatory signalling pathways involved in cell proliferation and cell death, which are similar to those regulated by DHA when used in pre-treatment, and both pre- and co-treatment with cortisol**

A total of 1 gene and 1 pathway were commonly regulated by cortisol (vs control) and DHA when used in pre-treatment (**DC**) (vs DHA alone, **DD**) (Table S3, S4). The “mTOR Complex 2 (mTORC2) Signaling” pathway with its gene chromosome 14 open reading frame 37 (C14orf37), is known to promote cell proliferation and cell development^8^. This gene was downregulated by both cortisol and DHA, therefore suggesting an inability for pre-treatment with DHA to contrast cortisol-induced reduction of the neuroprotective mTOR signalling pathway. Indeed, other pathways previously discussed in the Main Manuscript, like “Sirtuin Signaling Pathway” or “FGF Signaling” may instead mediate the neuroprotective effect of DHA against cortisol.

Furthermore, a total of 40 genes were regulated by both cortisol (**-C**) (vs control) and by pre- and co-treatment with DHA (**DDC**) (vs **DD**) (Table S3 and Figure S2e). Overall, these genes modulated 31 pathways (Table S4). Among them, of relevance is the “Cell Cycle: G1/S Checkpoint Regulation” pathway with its gene v-myc myelocytomatosis viral oncogene homolog avian (MYC), which plays a positive role in the regulation of cell cycle progression and in the inhibition of cell apoptosis^9^. In this case, MYC was upregulated not only by DHA but also by cortisol. As for EPA, this perhaps suggests that treatment with cortisol induces the activation of distinct neuroprotective compensatory pathways, similar to those activated by pre-treatment, and both pre- and co-treatment with DHA.

In addition, 75 genes were commonly regulated by DHA when used in pre-treatment (**DC**) (vs **DD**), and both pre- and co-treatment (**DDC**) (vs **DD**) (Table S3 and Figure S2f). Overall, these genes modulated 38 pathways (Table S4). Among them, the “transforming growth factor, beta (TGF-β) Signaling” pathway with its gene TGF-β2 is of particular interest. TGF-β2 is involved in maintaining cell quiescent and to inhibit cell proliferation^10^. This gene was indeed downregulated by both pre-treatment, and pre- and co-treatment with DHA.

Finally, 37 gene were commonly regulated by both by cortisol (**-C**) (vs control), and DHA used in pre-treatment (**DC**) (vs **DD**), and both pre- and co-treatment with cortisol (**DDC**) (vs **DD**) (Table S3 and Figure S2g). Overall, these genes modulated 18 pathways (Table S4). Of relevance is the “Chemokine Signaling” pathway with its gene chemokine (C-C motif) ligand 2 (CCL2). CCL2 promotes neuroinflammation, decreases proliferation and induces apoptosis^11^. This gene was upregulated not only by DHA but also by cortisol, again suggesting that treatment with cortisol is able to activate distinct neuroprotective compensatory pathways similar to those activated by pre-treatment, and both pre- and co-treatment with DHA.

**References**

1. Tsai YT *et al.* ANGPTL4 Induces TMZ Resistance of Glioblastoma by Promoting Cancer Stemness Enrichment via the EGFR/AKT/4E-BP1 Cascade. *International journal of molecular sciences* 2019; **20**(22).

2. Knobloch M *et al.* A Fatty Acid Oxidation-Dependent Metabolic Shift Regulates Adult Neural Stem Cell Activity. *Cell reports* 2017; **20**(9)**:** 2144-2155.

3. Ikonen M *et al.* Interaction between the Alzheimer's survival peptide humanin and insulin-like growth factor-binding protein 3 regulates cell survival and apoptosis. *Proceedings of the National Academy of Sciences of the United States of America* 2003; **100**(22)**:** 13042-13047.

4. Kalluri HS, Dempsey RJ. IGFBP-3 inhibits the proliferation of neural progenitor cells. *Neurochemical research* 2011; **36**(3)**:** 406-411.

5. Dazert P *et al.* Differential regulation of transport proteins in the periinfarct region following reversible middle cerebral artery occlusion in rats. *Neuroscience* 2006; **142**(4)**:** 1071-1079.

6. Broix L *et al.* Mutations in the HECT domain of NEDD4L lead to AKT-mTOR pathway deregulation and cause periventricular nodular heterotopia. *Nature genetics* 2016; **48**(11)**:** 1349-1358.

7. Ekholm SV, Reed SI. Regulation of G(1) cyclin-dependent kinases in the mammalian cell cycle. *Curr Opin Cell Biol* 2000; **12**(6)**:** 676-684.

8. LiCausi F, Hartman NW. Role of mTOR Complexes in Neurogenesis. *International journal of molecular sciences* 2018; **19**(5).

9. McMahon SB. MYC and the control of apoptosis. *Cold Spring Harb Perspect Med* 2014; **4**(7)**:** a014407.

10. Kandasamy M *et al.* TGF-beta signalling in the adult neurogenic niche promotes stem cell quiescence as well as generation of new neurons. *Journal of cellular and molecular medicine* 2014; **18**(7)**:** 1444-1459.

11. Zhang L *et al.* Effect of chemokine CC ligand 2 (CCL2) on alphasynucleininduced microglia proliferation and neuronal apoptosis. *Molecular medicine reports* 2018; **18**(5)**:** 4213-4218.

**Figure Caption**

**Figure S1. Representative interactions among gene clusters identified upon treatment with cortisol, EPA treatment alone, and EPA in pre-treatment, and both pre- and co-treatment with cortisol.** Every color corresponds to a cluster, whereas inter-cluster edges are represented by dashed-lines. Solid lines represent predicted functional edges of interaction between nodes (genes) within each cluster, where line colours represent the type of evidence and the predictive method used: green line, activation; red line, inhibition; blue line, binding; light blue line, phenotype; violet, catalyzes; pink, posttranslational mechanism; black, reaction; yellow, co-expression (<http://string-db.org>). No line means no interaction.

**Figure S2. Representative interactions among gene clusters identified upon treatment with cortisol, DHA treatment alone, and DHA in pre-treatment, and both pre- and co-treatment with cortisol.** Every color corresponds to a cluster, whereas inter-cluster edges are represented by dashed-lines. Solid lines represent predicted functional edges of interaction between nodes (genes) within each cluster, where line colours represent the type of evidence and the predictive method used: green line, activation; red line, inhibition; blue line, binding; light blue line, phenotype; violet, catalyzes; pink, posttranslational mechanism; black, reaction; yellow, co-expression (<http://string-db.org>). No line means no interaction.
