# Supplementary material for: The role of omega-3 fatty acids in preventing glucocorticoid-induced reduction in human hippocampal neurogenesis and increase in apoptosis
Source: Transl Psychiatry. 2020 Jul 7;10:219. doi: 10.1038/s41398-020-00908-0 (PMC7341841; doi:10.1038/s41398-020-00908-0)

a) EthOH vs Cortisol


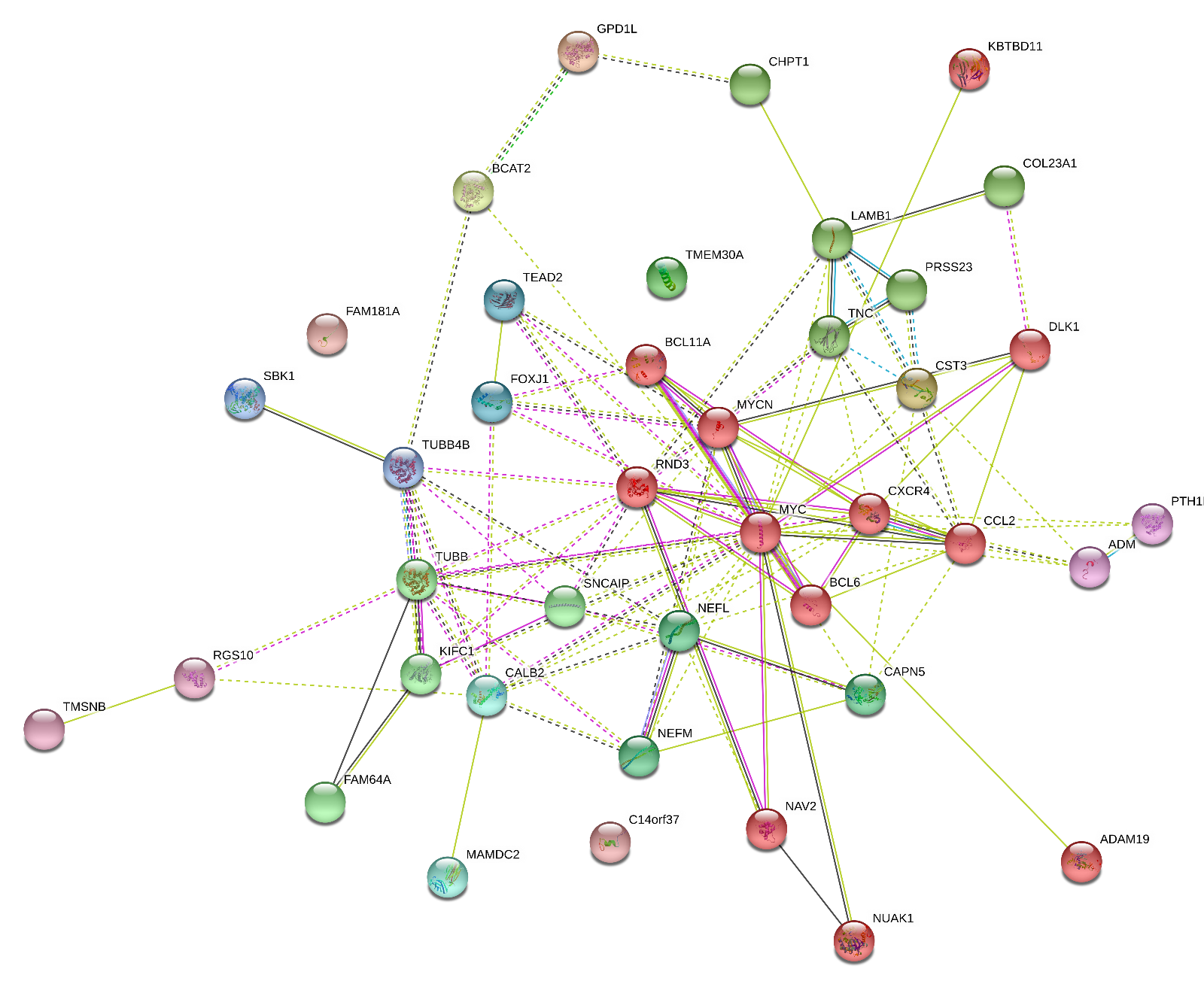


b) EE vs EC


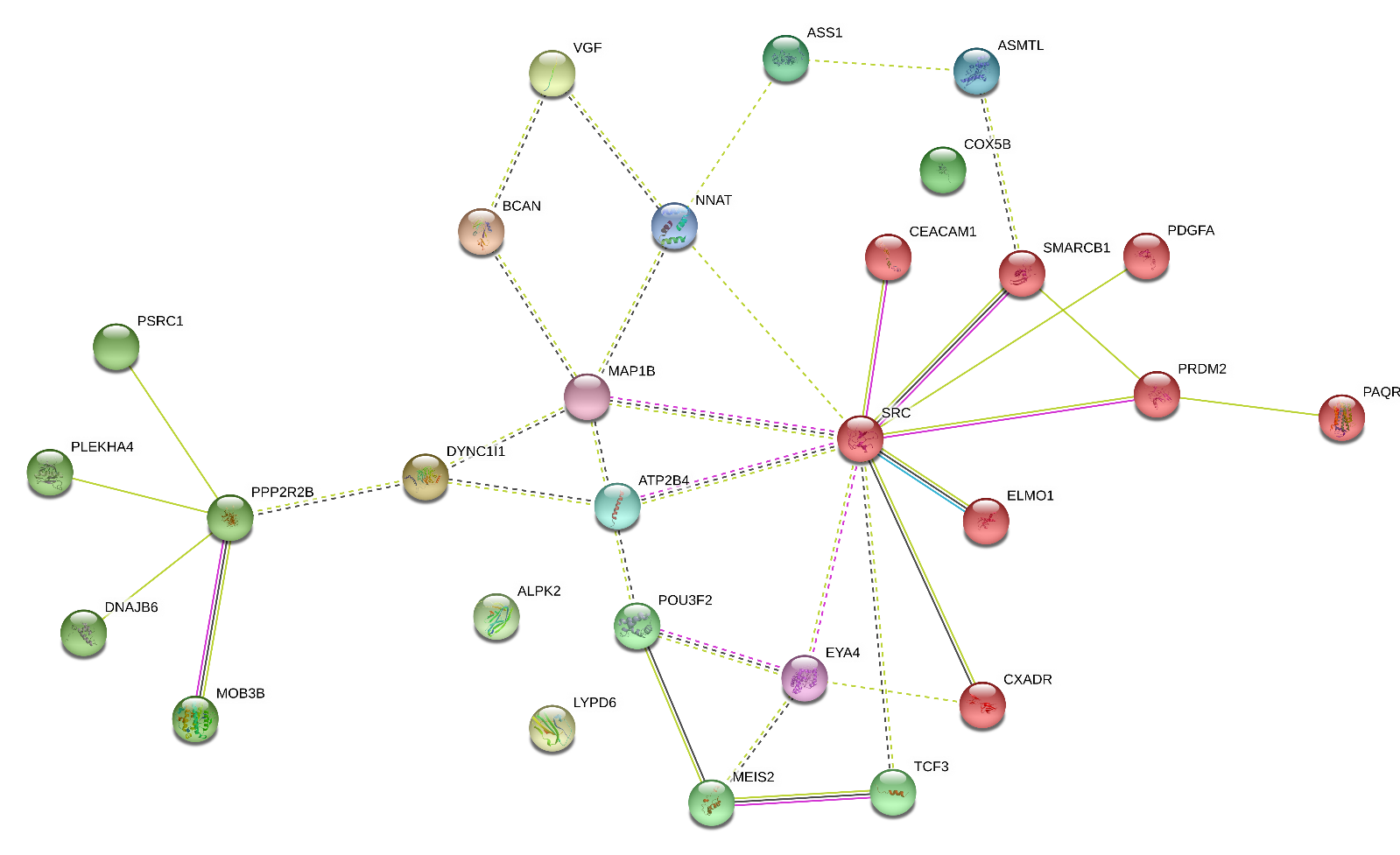


c) EE vs EEC


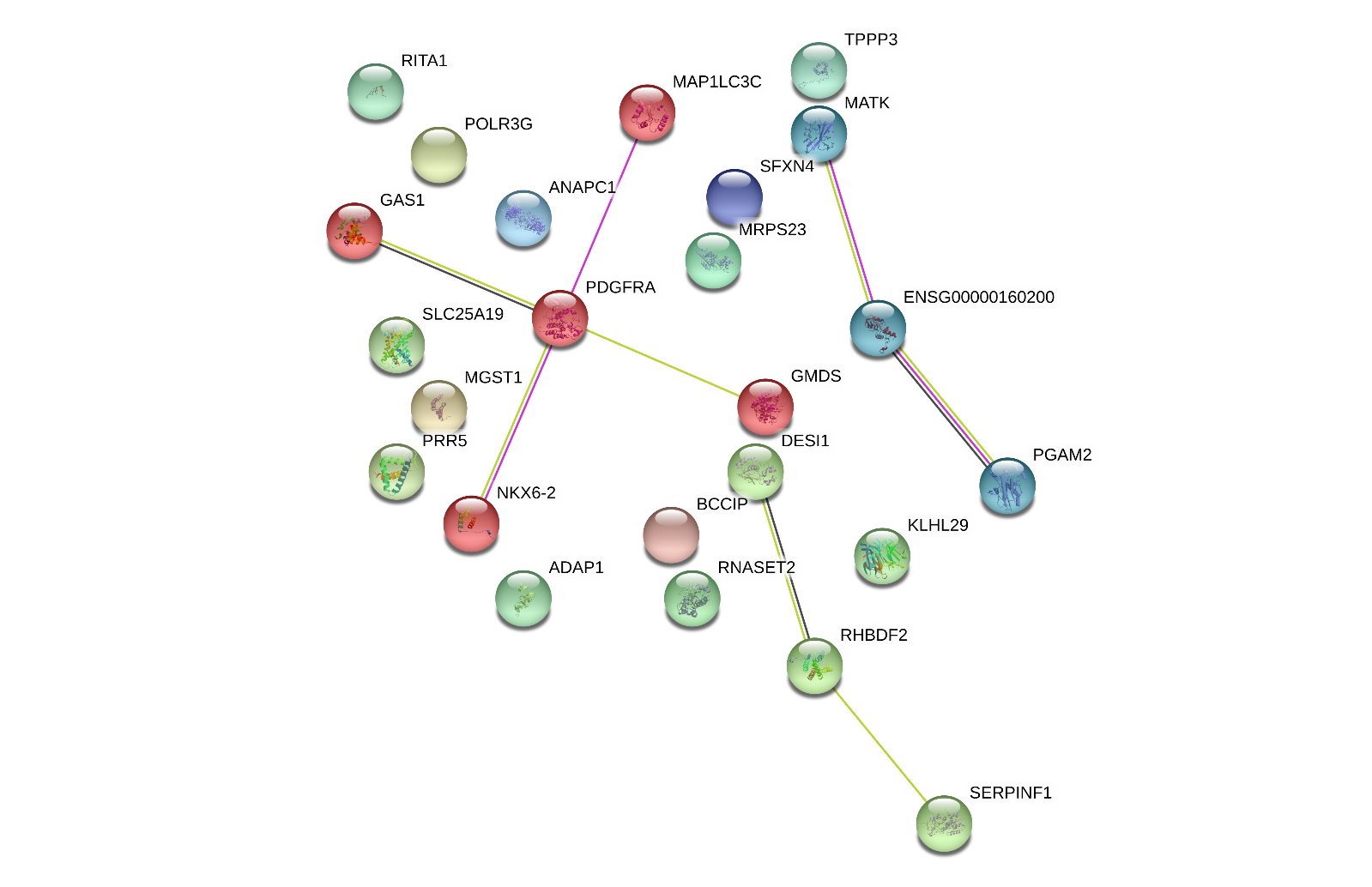


d) EtOH vs EE


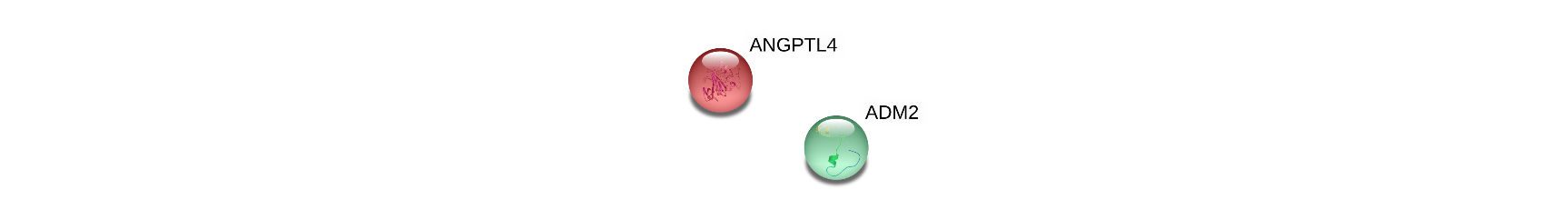


e) EthOH vs Cortisol_EE vs EC


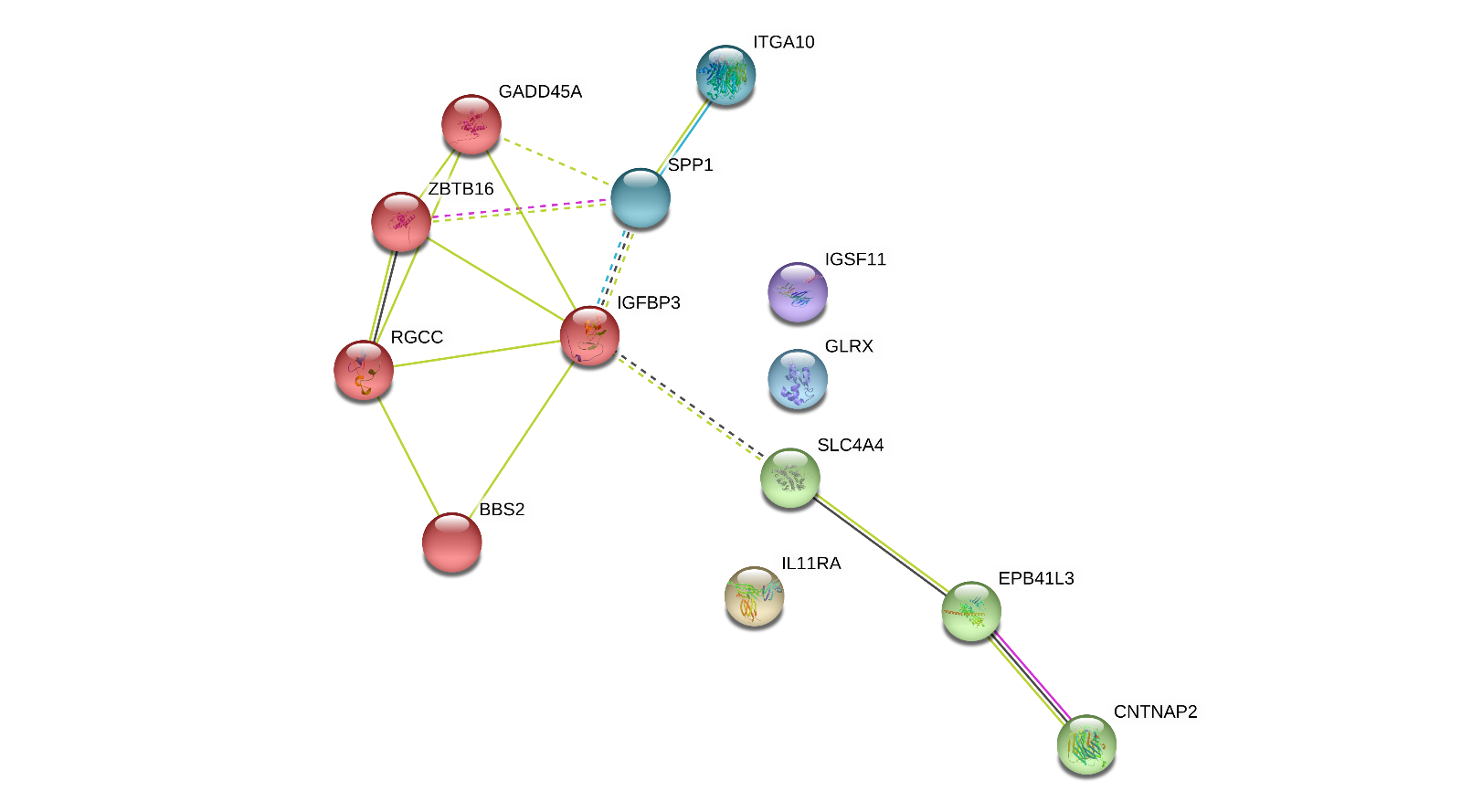


f) EthOH vs Cortisol_EE vs EEC


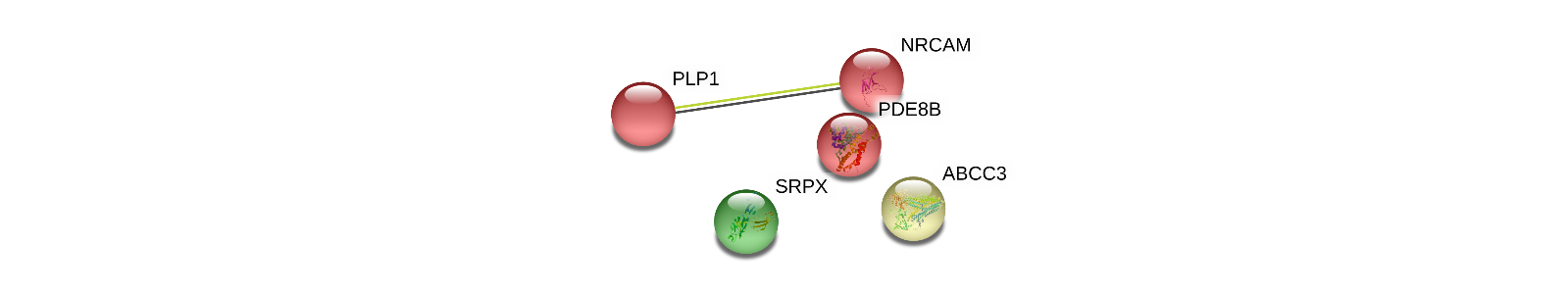


g) EE vs EC_ EE vs EEC


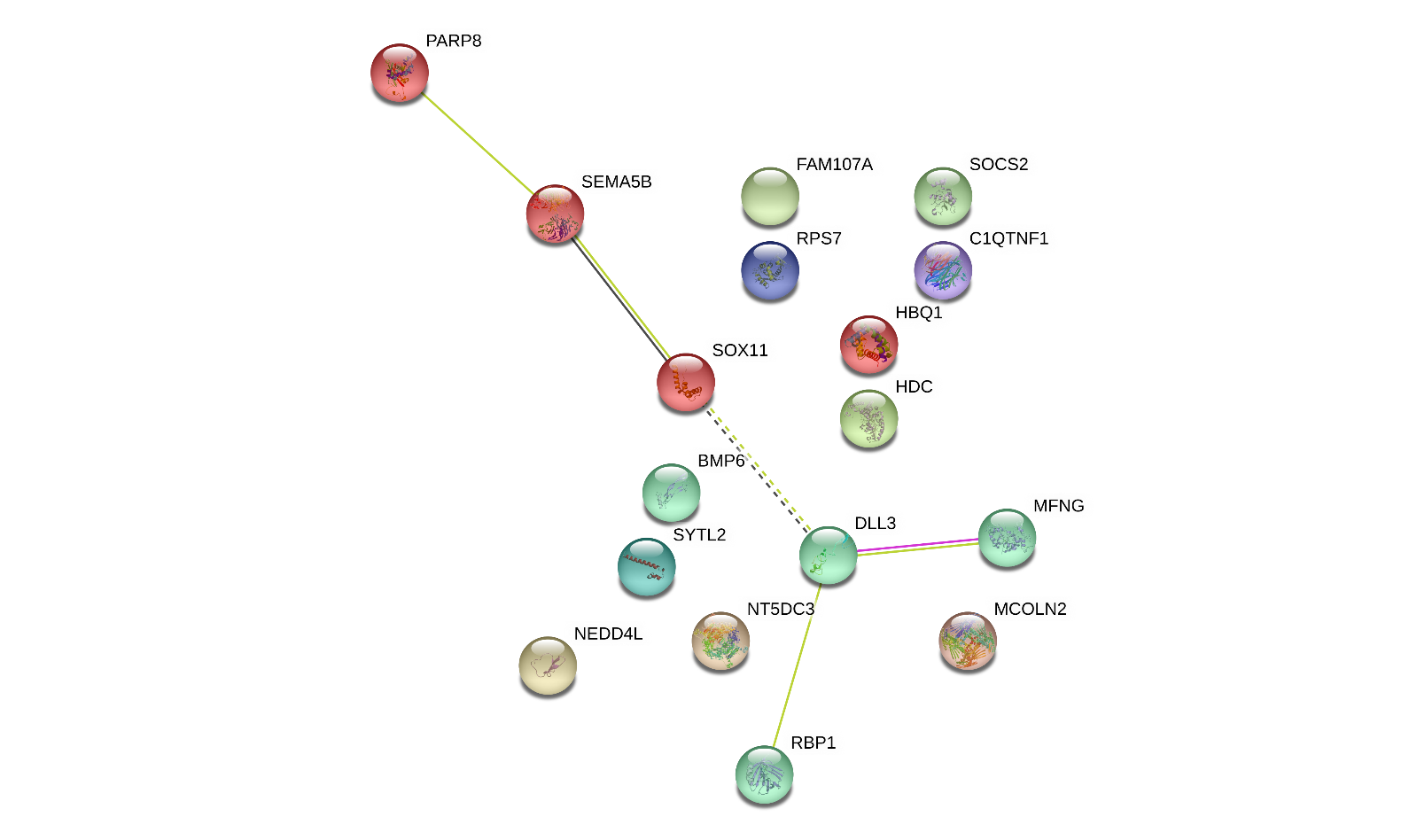


h) All conditions


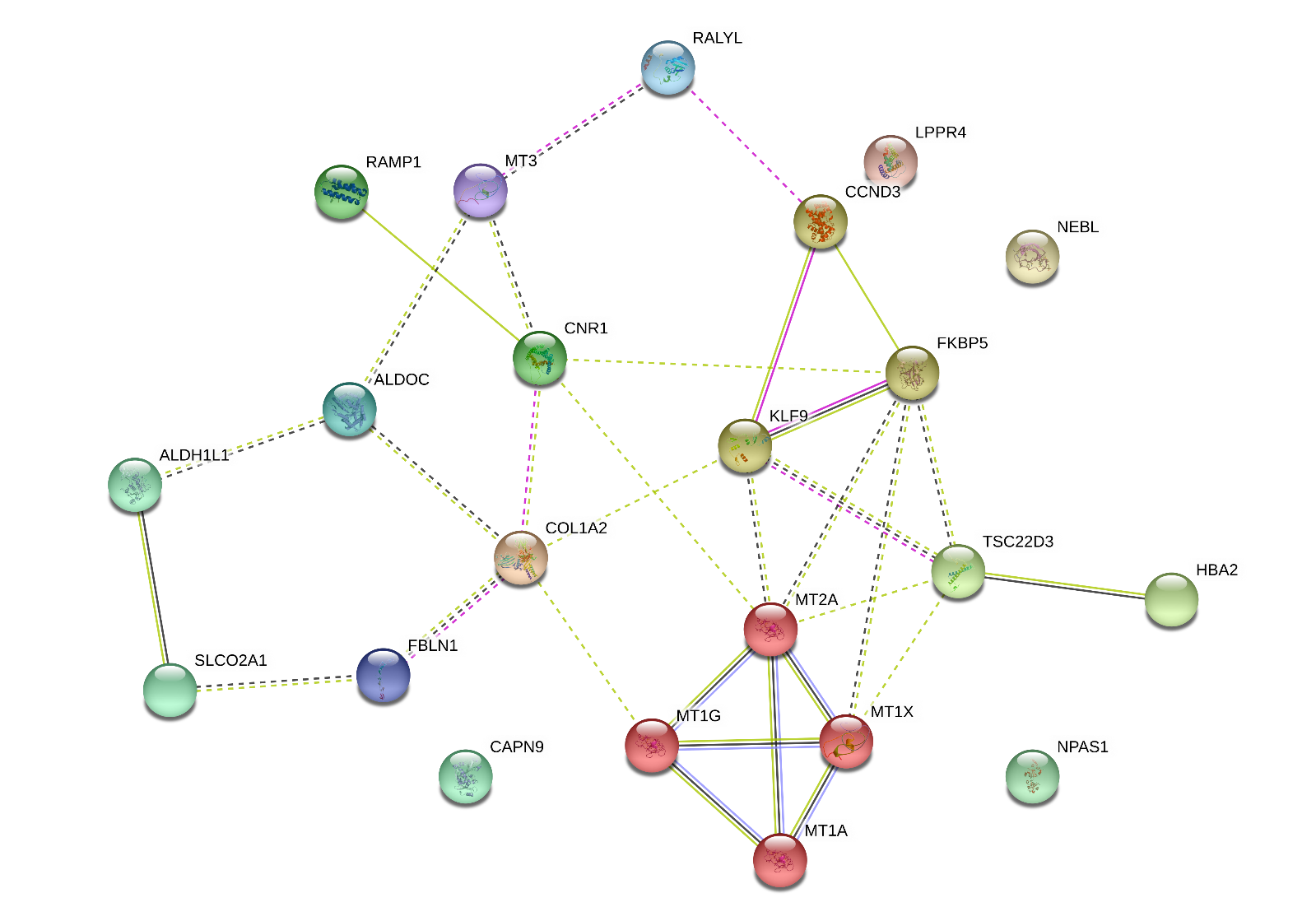

Supplement: Supplementary file 2 — Figure S1 [file 41398_2020_908_MOESM2_ESM.docx]
