# Supplementary material for: The role of omega-3 fatty acids in preventing glucocorticoid-induced reduction in human hippocampal neurogenesis and increase in apoptosis
Source: Transl Psychiatry. 2020 Jul 7;10:219. doi: 10.1038/s41398-020-00908-0 (PMC7341841; doi:10.1038/s41398-020-00908-0)

a) EthOH vs Cortisol


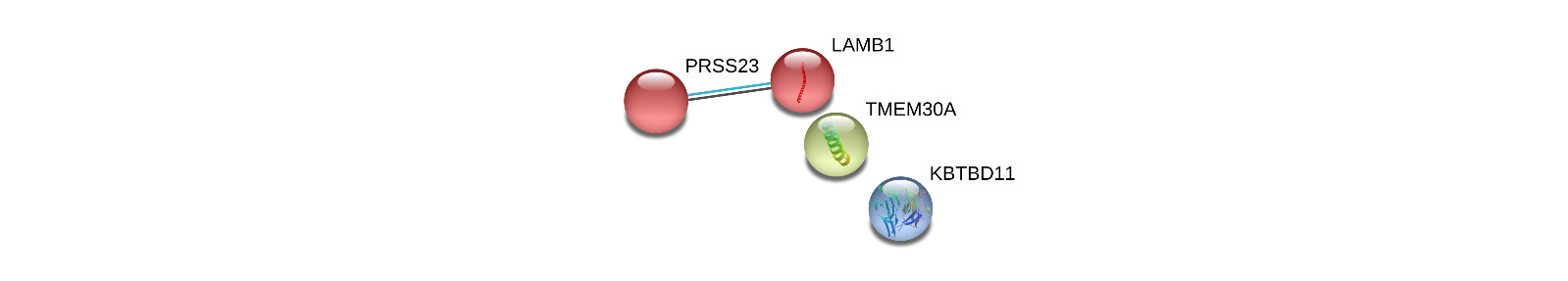


b) DD vs DC


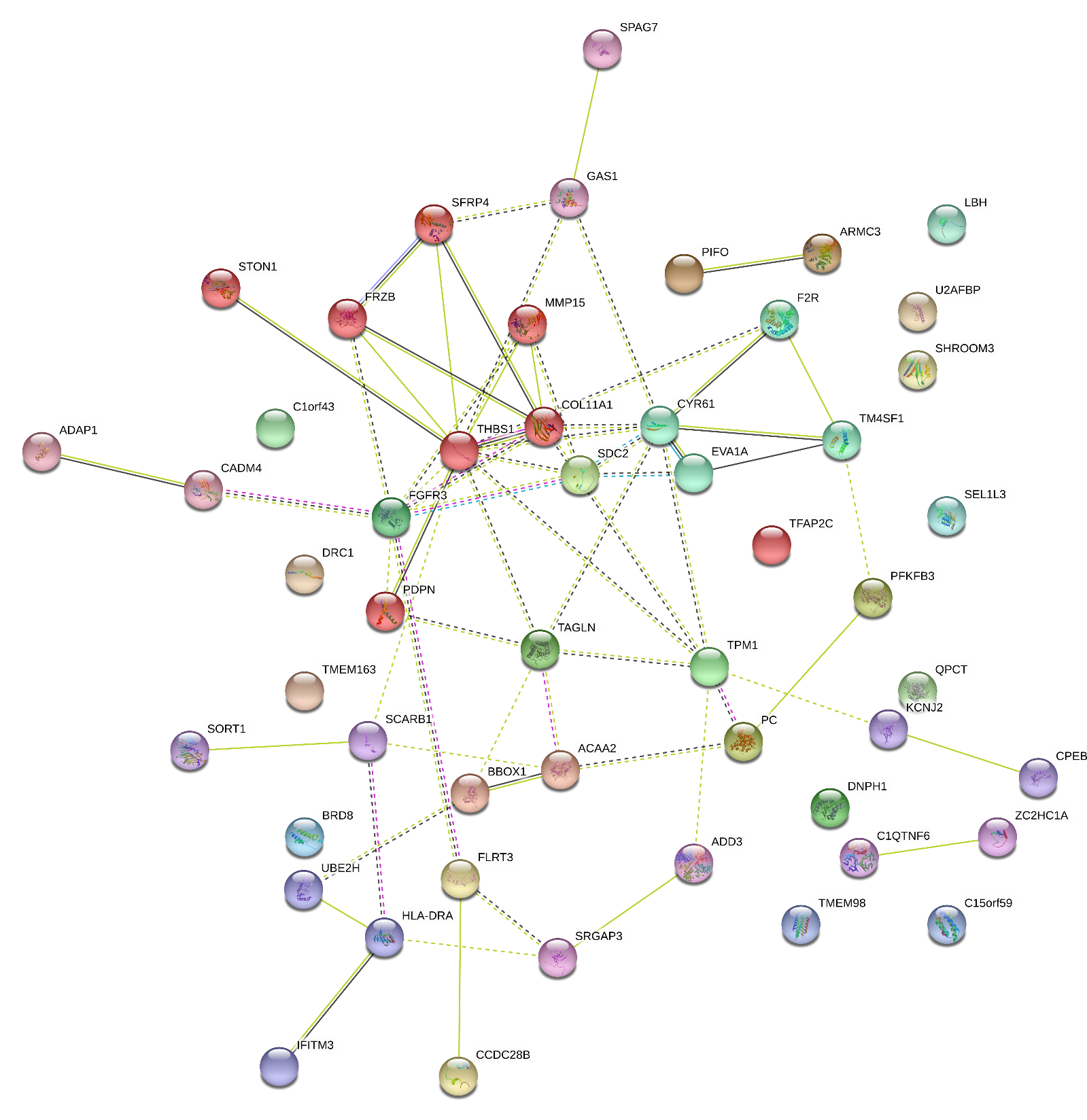


c) DD vs DDC


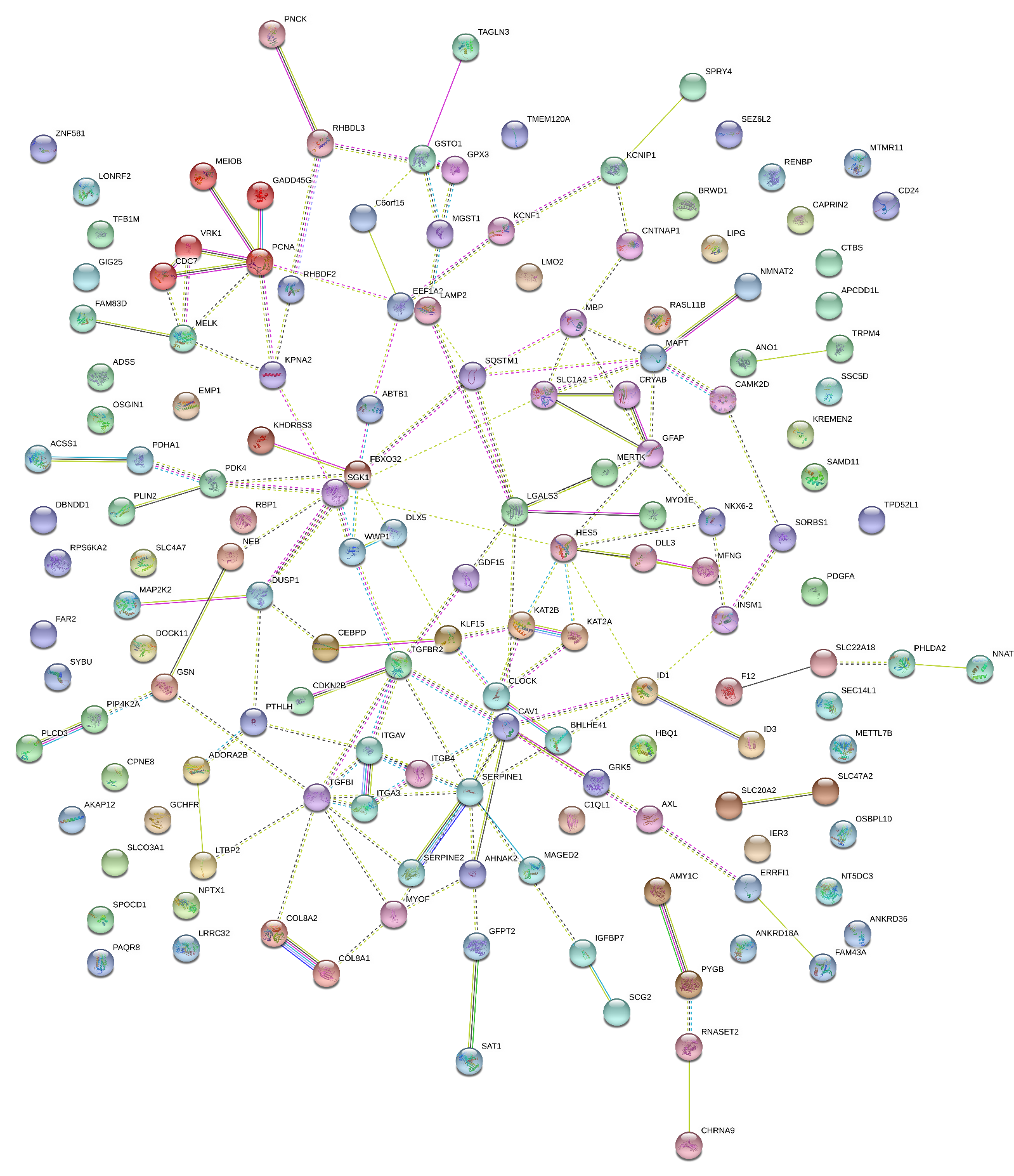


*the figure includes only genes with a fold change ≥ +1.4 and ≤ -1.4

d) EtOH vs DD


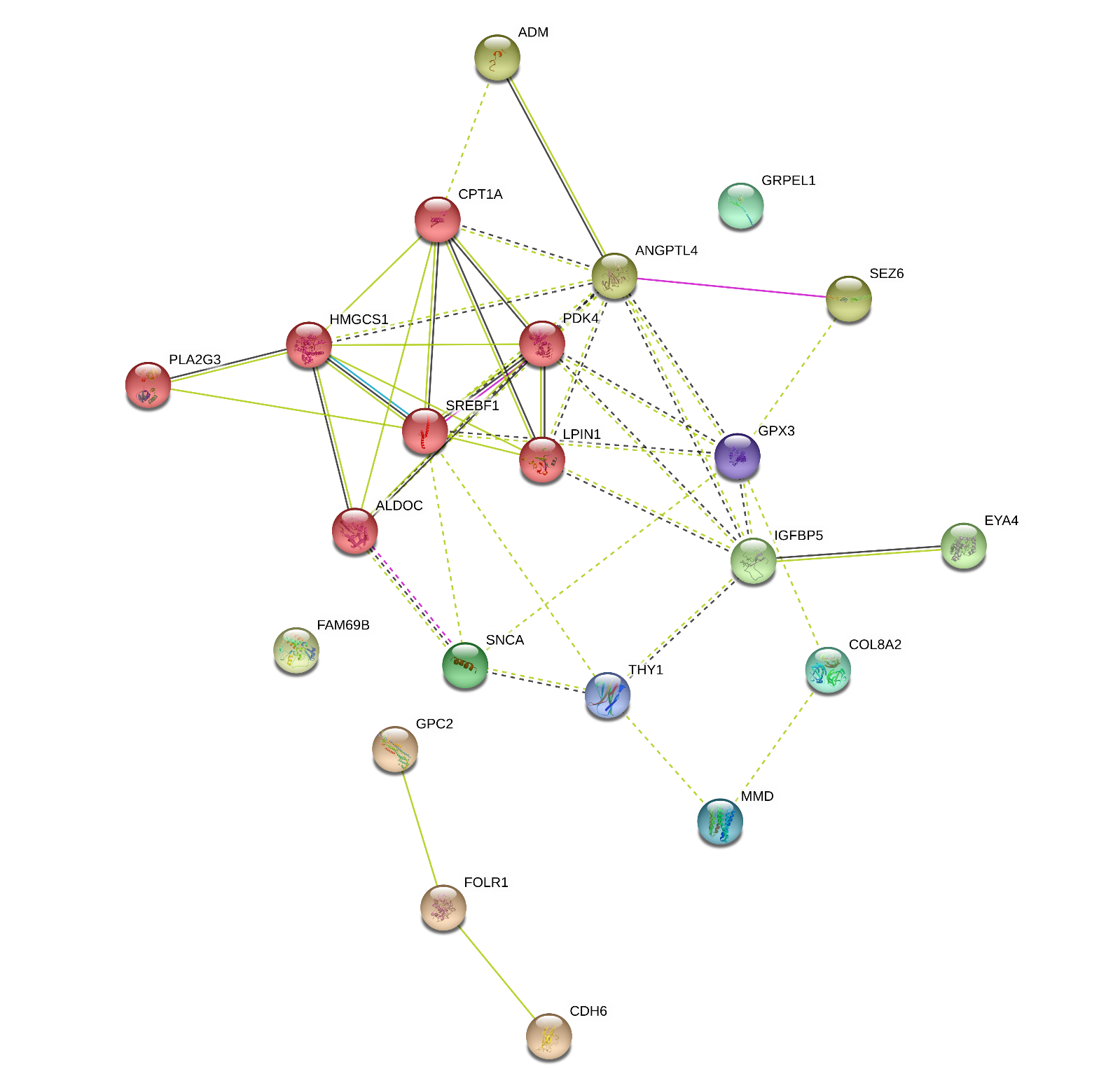


f) EthOH vs Cortisol_DD vs DDC


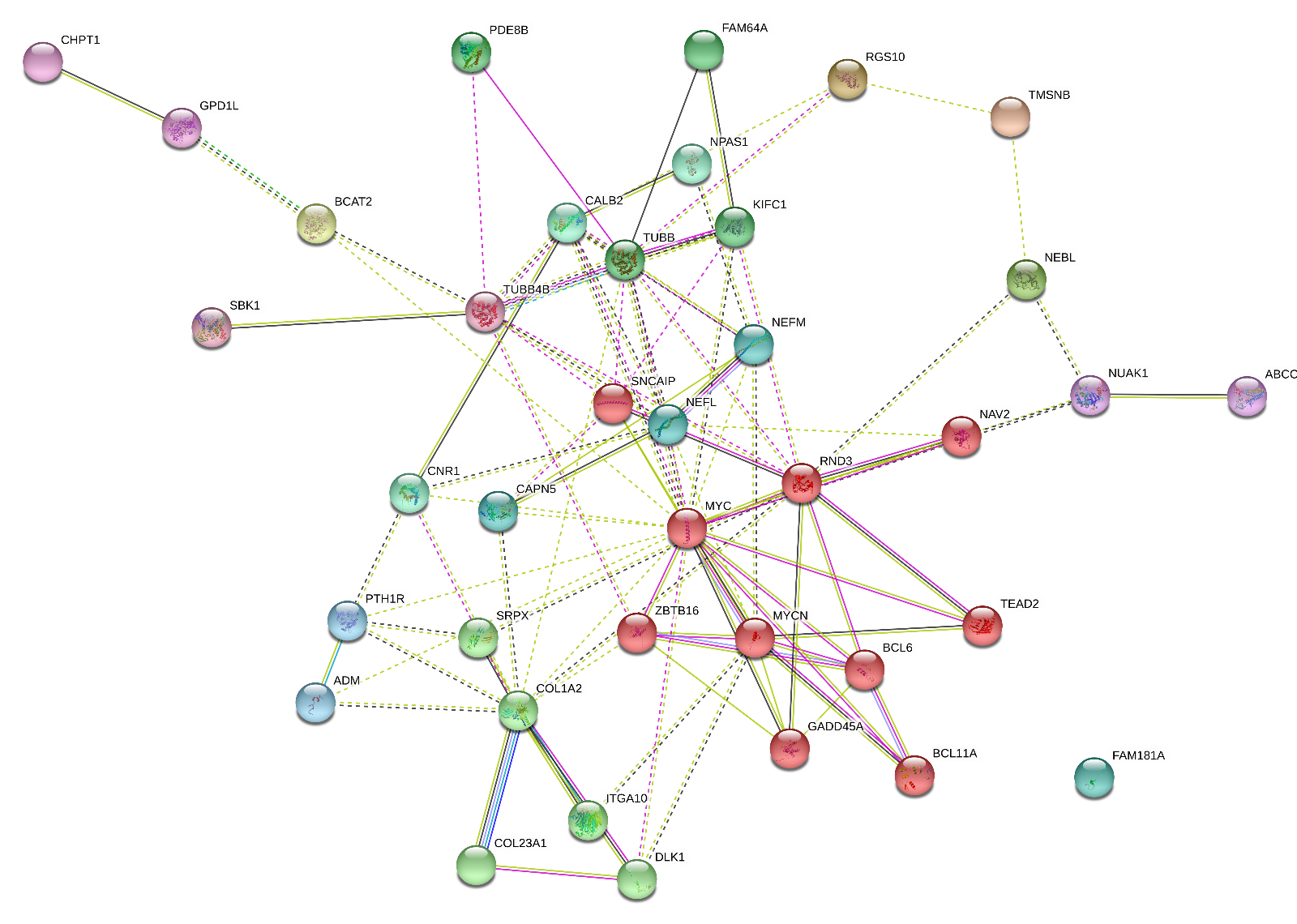


g) DD vs DC_ DD vs DDC


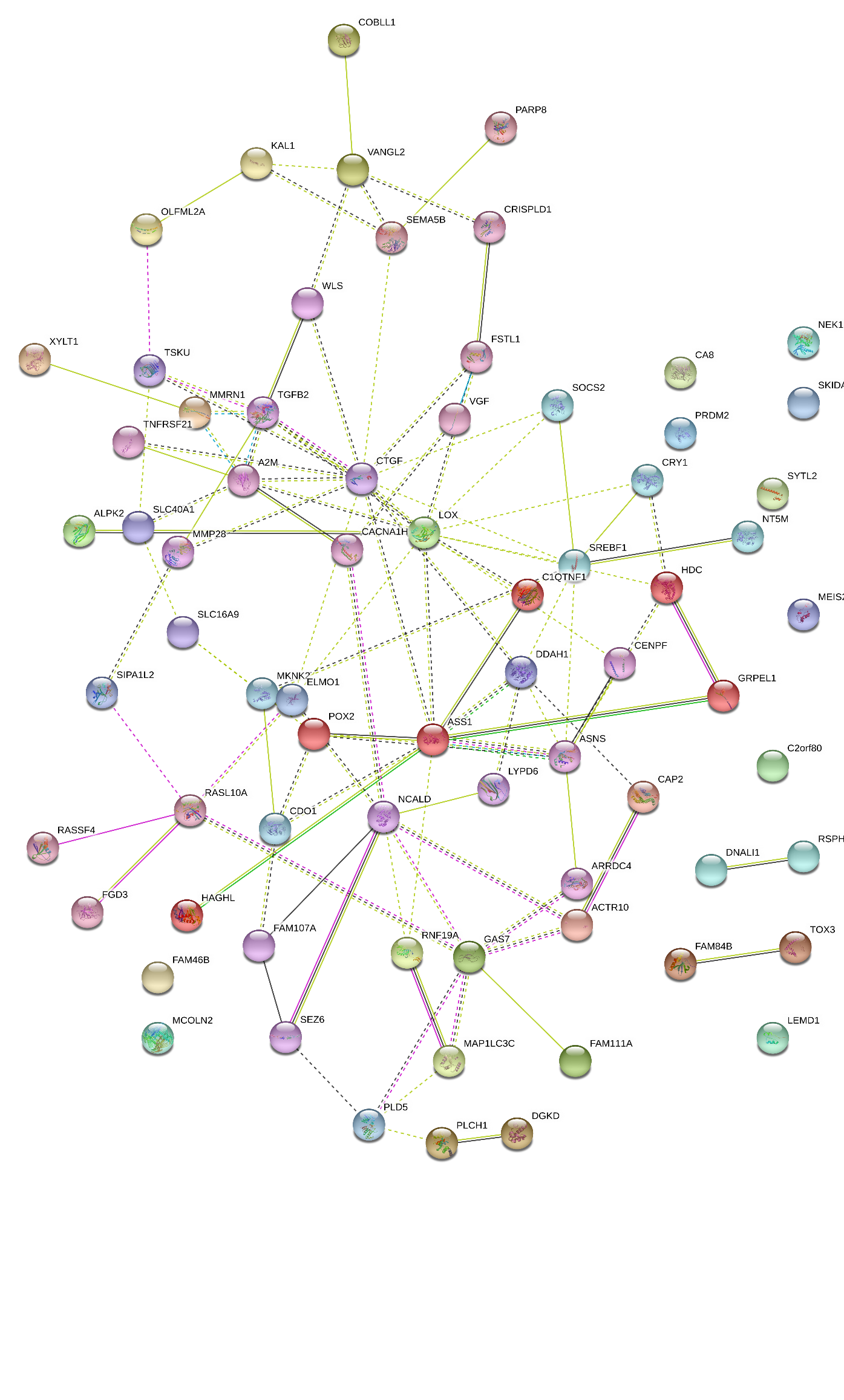


h) All conditions


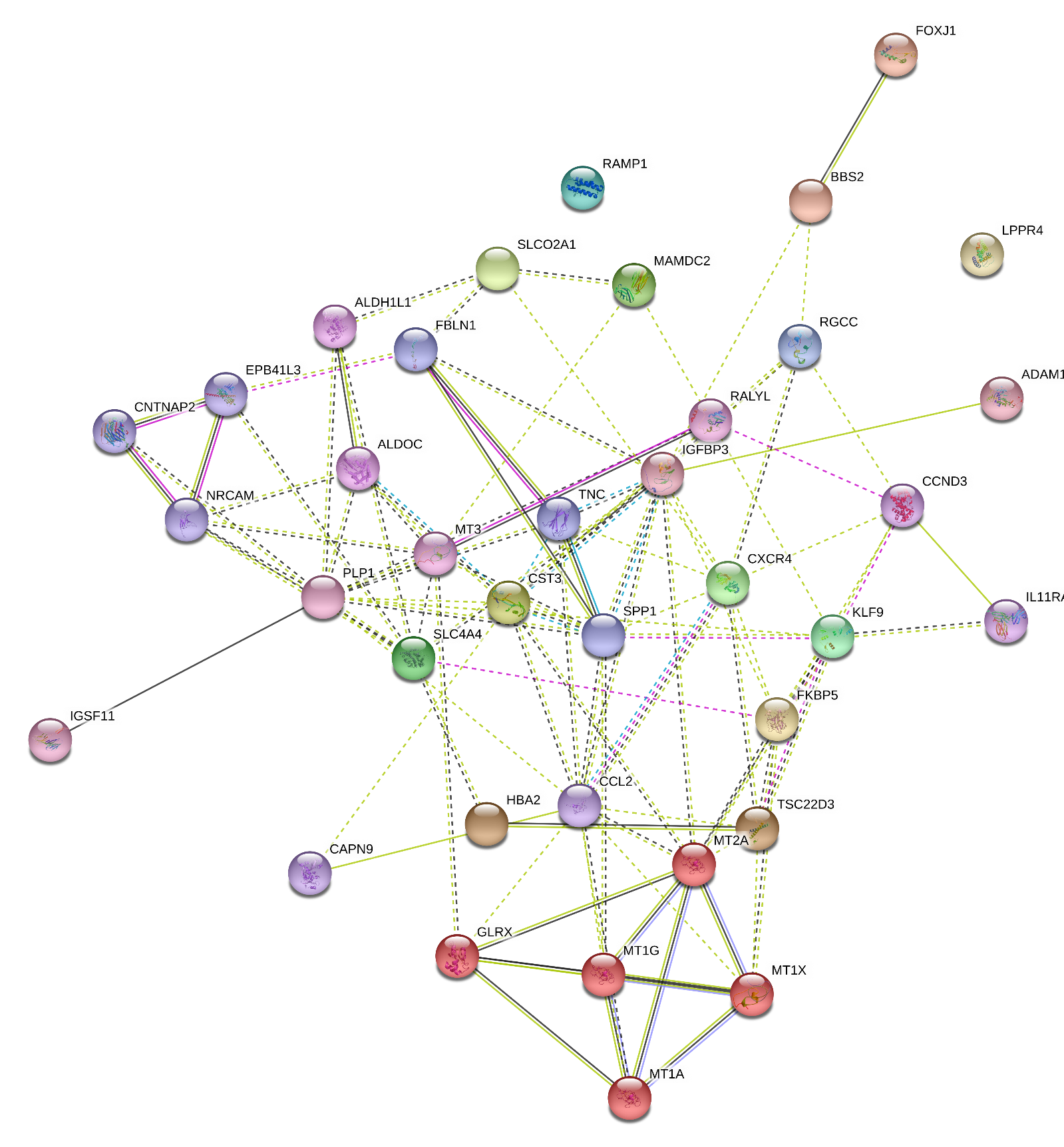

Supplement: Supplementary file 3 — Figure S2 [file 41398_2020_908_MOESM3_ESM.docx]
