# Supplementary material for: The role of omega-3 fatty acids in preventing glucocorticoid-induced reduction in human hippocampal neurogenesis and increase in apoptosis
Source: Transl Psychiatry. 2020 Jul 7;10:219. doi: 10.1038/s41398-020-00908-0 (PMC7341841; doi:10.1038/s41398-020-00908-0)
Supplement: Supplementary file 4 — Table S1 [file 41398_2020_908_MOESM4_ESM.docx]

**Table S1.** Genes regulated by cortisol, EPA alone and by EPA in pre-treatment, and both pre- and co-treatment with cortisol.

| **Gene Name** | **EtOH vs Cortisol** | | | **Fold Change** | | **q value (%)** | |
| --- | --- | --- | --- | --- | --- | --- | --- |
| Homo sapiens adrenomedullin (ADM), mRNA. | ADM | | | 1.96 | | 0 | |
| Homo sapiens small nucleolar RNA, C/D box 3D (SNORD3D), small nucleolar RNA. | SNORD3D | | | 1.88 | | 3.93 | |
| Homo sapiens delta-like 1 homolog (Drosophila) (DLK1), mRNA. | DLK1 | | | 1.82 | | 3.93 | |
| Homo sapiens B-cell CLL/lymphoma 6 (zinc finger protein 51) (BCL6), transcript variant 2, mRNA. | BCL6 | | | 1.74 | | 1.66 | |
| Homo sapiens neurofilament, light polypeptide 68kDa (NEFL), mRNA. | NEFL | | | 1.61 | | 1.66 | |
| Homo sapiens v-myc myelocytomatosis viral oncogene homolog (avian) (MYC), mRNA. | MYC | | | 1.60 | | 0 | |
| Homo sapiens neurofilament, medium polypeptide 150kDa (NEFM), mRNA. | NEFM | | | 1.60 | | 3.09 | |
| Homo sapiens small nucleolar RNA, C/D box 3C (SNORD3C), small nucleolar RNA. | SNORD3C | | | 1.60 | | 1.66 | |
| Homo sapiens choline phosphotransferase 1 (CHPT1), mRNA. | CHPT1 | | | 1.59 | | 3.09 | |
| Homo sapiens cystatin C (CST3), mRNA. | CST3 | | | 1.54 | | 3.93 | |
| Homo sapiens NUAK family, SNF1-like kinase, 1 (NUAK1), mRNA. | NUAK1 | | | 1.54 | | 0 | |
| Homo sapiens neuron navigator 2 (NAV2), transcript variant 2, mRNA. | NAV2 | | | 1.52 | | 0 | |
| Homo sapiens collagen, type XXIII, alpha 1 (COL23A1), mRNA. | COL23A1 | | | 1.51 | | 1.66 | |
| Homo sapiens regulator of G-protein signaling 10 (RGS10), transcript variant 1, mRNA. | RGS10 | | | 1.49 | | 1.66 | |
| Homo sapiens laminin, beta 1 (LAMB1), mRNA. | LAMB1 | | | 1.49 | | 3.93 | |
| Homo sapiens branched chain aminotransferase 2, mitochondrial (BCAT2), nuclear gene encoding mitochondrial protein, mRNA. | BCAT2 | | | 1.47 | | 3.93 | |
| Homo sapiens DEAD/H-Box Helicase 12, Pseudogene | DDX12 | | | 1.45 | | 3.09 | |
| Homo sapiens kelch repeat and BTB (POZ) domain containing 11 (KBTBD11), mRNA. | KBTBD11 | | | 1.42 | | 3.93 | |
| Homo sapiens transmembrane protein 30A (TMEM30A), mRNA. | TMEM30A | | | 1.40 | | 3.93 | |
| Homo sapiens glycerol-3-phosphate dehydrogenase 1-like (GPD1L), mRNA. | GPD1L | | | 1.37 | | 1.66 | |
| Homo sapiens parathyroid hormone 1 receptor (PTH1R), mRNA. | PTH1R | | | 1.33 | | 3.93 | |
| Homo sapiens chromosome 14 open reading frame 37 (C14orf37), mRNA. | C14ORF37 | | | -1.30 | | 4.66 | |
| Homo sapiens family with sequence similarity 64, member A (FAM64A), mRNA. | FAM64A | | | -1.31 | | 4.66 | |
| Homo sapiens SH3-binding domain kinase 1 (SBK1), mRNA. | SBK1 | | | -1.32 | | 4.66 | |
| Homo sapiens tubulin, beta (TUBB), mRNA. | TUBB | | | -1.32 | | 4.66 | |
| Homo sapiens Rho family GTPase 3 (RND3), mRNA. | RND3 | | | -1.34 | | 4.66 | |
| Homo sapiens B-cell CLL/lymphoma 11A (zinc finger protein) (BCL11A), transcript variant 5, mRNA. | BCL11A | | | -1.34 | | 4.66 | |
| Homo sapiens kinesin family member C1 (KIFC1), mRNA. | KIFC1 | | | -1.35 | | 4.66 | |
| Homo sapiens ADAM metallopeptidase domain 19 (meltrin beta) (ADAM19), mRNA. | ADAM19 | | | -1.35 | | 4.66 | |
| Homo sapiens MAM domain containing 2 (MAMDC2), mRNA. | MAMDC2 | | | -1.36 | | 0 | |
| Homo sapiens TEA domain family member 2 (TEAD2), mRNA. | TEAD2 | | | -1.36 | | 4.66 | |
| Homo sapiens calpain 5 (CAPN5), mRNA. | CAPN5 | | | -1.38 | | 4.66 | |
| Homo sapiens chemokine (C-X-C motif) receptor 4 (CXCR4), transcript variant 1, mRNA. | CXCR4 | | | -1.39 | | 4.66 | |
| Homo sapiens v-myc myelocytomatosis viral related oncogene, neuroblastoma derived (avian) (MYCN), mRNA. | MYCN | | | -1.39 | | 4.66 | |
| Homo sapiens family with sequence similarity 181, member A (FAM181A), mRNA. | FAM181A | | | -1.40 | | 1.97 | |
| Homo sapiens tubulin, beta 2C (TUBB2C), mRNA. | TUBB2C | | | -1.40 | | 0 | |
| Homo sapiens tenascin C (hexabrachion) (TNC), mRNA. | TNC | | | -1.40 | | 4.66 | |
| Homo sapiens protease, serine, 23 (PRSS23), mRNA. | PRSS23 | | | -1.40 | | 0 | |
| Homo sapiens synuclein, alpha interacting protein (SNCAIP), mRNA. | SNCAIP | | | -1.40 | | 4.66 | |
| Homo sapiens forkhead box J1 (FOXJ1), mRNA. | FOXJ1 | | | -1.42 | | 4.66 | |
| Homo sapiens chemokine (C-C motif) ligand 22 (CCL22), mRNA. | CCL2 | | | -1.50 | | 0 | |
| Homo sapiens thymosin beta 15a (TMSB15A), mRNA. | TMSB15A | | | -1.51 | | 0 | |
| Homo sapiens calbindin 2, 29kDa (calretinin) (CALB2), transcript variant CALB2c, mRNA. | CALB2 | | | -1.59 | | 4.66 | |
|  | | | | | | | |
| **Gene Name** | **EtOH vs EE** | | | **Fold Change** | | **q value (%)** | |
| Homo sapiens angiopoietin-like 4 (ANGPTL4), transcript variant 3, mRNA. | ANGPTL4 | | | 3.53 | | 0 | |
| Homo sapiens adrenomedullin 2 (ADM2), mRNA. | ADM2 | | | 1.60 | | 0 | |
|  | | | | | | | |
| **Gene Name** | **EE vs EC** | | | **Fold Change** | | **q value (%)** | |
| Homo sapiens VGF nerve growth factor inducible (VGF), mRNA. | VGF | | | 2.58 | | 0 | |
| Homo sapiens progestin and adipoQ receptor family member VIII (PAQR8), mRNA. | PAQR8 | | | 1.74 | | 0 | |
| Homo sapiens metallothionein E (MTE), mRNA. | MTE | | | 1.58 | | 0 | |
| Homo sapiens platelet-derived growth factor alpha polypeptide (PDGFA), transcript variant 2, mRNA. | PDGFA | | | 1.49 | | 2.83 | |
| Homo sapiens small nucleolar RNA, H/ACA box 18 (SNORA18), small nucleolar RNA. | SNORA18 | | | 1.45 | | 4.24 | |
| Homo sapiens cytochrome c oxidase subunit Vb (COX5B), mRNA. | COX5B | | | 1.42 | | 4.24 | |
| Homo sapiens acetylserotonin O-methyltransferase-like (ASMTL), mRNA. | ASMTL | | | 1.37 | | 4.24 | |
| Homo sapiens protein phosphatase 2 (formerly 2A), regulatory subunit B, beta isoform (PPP2R2B), transcript variant 1, mRNA. | PPP2R2B | | | 1.30 | | 4.24 | |
| Homo sapiens proline/serine-rich coiled-coil 1 (PSRC1), transcript variant 4, mRNA. | PSRC1 | | | -1.25 | | 3.66 | |
| Homo sapiens coxsackie virus and adenovirus receptor (CXADR), mRNA. | CXADR | | | -1.28 | | 2.05 | |
| Homo sapiens ATPase, Ca++ transporting, plasma membrane 4 (ATP2B4), transcript variant 1, mRNA. | ATP2B4 | | | -1.28 | | 2.83 | |
| Homo sapiens v-src sarcoma (Schmidt-Ruppin A-2) viral oncogene homolog (avian) (SRC), transcript variant 1, mRNA. | SRC | | | -1.29 | | 3.66 | |
| Homo sapiens dynein, cytoplasmic 1, intermediate chain 1 (DYNC1I1), mRNA. | DYNC1I1 | | | -1.29 | | 3.66 | |
| Homo sapiens DnaJ (Hsp40) homolog, subfamily B, member 6 (DNAJB6), transcript variant 2, mRNA. | DNAJB6 | | | -1.30 | | 3.66 | |
| Homo sapiens eyes absent homolog 4 (Drosophila) (EYA4), transcript variant 1, mRNA. | EYA4 | | | -1.30 | | 2.83 | |
| Homo sapiens Meis homeobox 2 (MEIS2), transcript variant a, mRNA. | MEIS2 | | | -1.31 | | 4.24 | |
| Homo sapiens microtubule-associated protein 1B (MAP1B), transcript variant 2, mRNA. | MAP1B | | | -1.31 | | 2.05 | |
| Homo sapiens LY6/PLAUR domain containing 6 (LYPD6), mRNA. | LYPD6 | | | -1.31 | | 2.05 | |
| Homo sapiens transcription factor 3 (E2A immunoglobulin enhancer binding factors E12/E47) (TCF3), mRNA. | TCF3 | | | -1.32 | | 2.83 | |
| Homo sapiens carcinoembryonic antigen-related cell adhesion molecule 1 (biliary glycoprotein) (CEACAM1), transcript variant 1, mRNA. | CEACAM1 | | | -1.32 | | 3.66 | |
| Homo sapiens SWI/SNF related, matrix associated, actin dependent regulator of chromatin, subfamily b, member 1 (SMARCB1), transcript variant 2, mRNA. | SMARCB1 | | | -1.33 | | 2.83 | |
| Homo sapiens POU class 3 homeobox 2 (POU3F2), mRNA. | POU3F2 | | | -1.33 | | 4.24 | |
| Homo sapiens MOB1, Mps One Binder kinase activator-like 2B (yeast) (MOBKL2B), mRNA. | MOBKL2B | | | -1.35 | | 2.05 | |
| Homo sapiens pleckstrin homology domain containing, family A (phosphoinositide binding specific) member 4 (PLEKHA4), mRNA. | PLEKHA4 | | | -1.35 | | 3.66 | |
| Homo sapiens brevican (BCAN), transcript variant 1, mRNA. | BCAN | | | -1.36 | | 2.05 | |
| Homo sapiens Myocardial Infarction Associated Transcript | MIAT | | | -1.38 | | 4.24 | |
| Homo sapiens argininosuccinate synthetase 1 (ASS1), transcript variant 1, mRNA. | ASS1 | | | -1.39 | | 0 | |
| Homo sapiens alpha-kinase 2 (ALPK2), mRNA. | ALPK2 | | | -1.41 | | 0 | |
| Homo sapiens engulfment and cell motility 1 (ELMO1), transcript variant 1, mRNA. | ELMO1 | | | -1.42 | | 0 | |
| Homo sapiens neuronatin (NNAT), transcript variant 1, mRNA. | NNAT | | | -1.43 | | 0 | |
|  | | | | | | | |
| **Gene Name** | **EE vs EEC** | | | **Fold Change** | | **q value (%)** | |
| Homo sapiens NK6 homeobox 2 (NKX6-2), mRNA. | NKX6-2 | | | 1.90 | | 3.20 | |
| Homo sapiens solute carrier family 25 (mitochondrial thiamine pyrophosphate carrier), member 19 (SLC25A19), nuclear gene encoding mitochondrial protein, mRNA. | SLC25A19 | | | 1.60 | | 2.27 | |
| Homo sapiens ribonuclease T2 (RNASET2), mRNA. | RNASET2 | | | 1.58 | | 3.20 | |
| Homo sapiens BRCA2 and CDKN1A interacting protein (BCCIP), transcript variant B, mRNA. | BCCIP | | | 1.57 | | 3.20 | |
| Homo sapiens GDP-mannose 4,6-dehydratase (GMDS), mRNA. | GMDS | | | 1.55 | | 2.27 | |
| Homo sapiens microsomal glutathione S-transferase 1 (MGST1), transcript variant 1d, mRNA. | MGST1 | | | 1.49 | | 0 | |
| Homo sapiens centaurin, alpha 1 (CENTA1), mRNA. | CENTA1 | | | 1.49 | | 2.27 | |
| Homo sapiens polymerase (RNA) III (DNA directed) polypeptide G (32kD)-like (POLR3GL), mRNA. | POLR3G | | | 1.48 | | 3.20 | |
| Homo sapiens rhomboid 5 homolog 2 (Drosophila) (RHBDF2), transcript variant 1, mRNA. | RHBDF2 | | | 1.48 | | 0 | |
| Homo sapiens mitochondrial ribosomal protein S23 (MRPS23), nuclear gene encoding mitochondrial protein, mRNA. | MRPS23 | | | 1.45 | | 4.02 | |
| Homo sapiens anaphase promoting complex subunit 1 (ANAPC1), transcript variant 1, mRNA. | ANAPC1 | | | 1.39 | | 3.20 | |
| Homo sapiens chromosome 12 open reading frame 52 (C12orf52), mRNA. | C12ORF52 | | | 1.38 | | 4.02 | |
| Homo sapiens sideroflexin 4 (SFXN4), mRNA. | SFXN4 | | | 1.37 | | 3.20 | |
| Homo sapiens megakaryocyte-associated tyrosine kinase (MATK), transcript variant 3, mRNA. | MATK | | | 1.36 | | 2.27 | |
| Homo sapiens PPPDE peptidase domain containing 2 (PPPDE2), mRNA. | PPPDE2 | | | 1.36 | | 2.27 | |
| Homo sapiens proline rich 5 (renal) (PRR5), transcript variant 4, mRNA. | PRR5 | | | 1.33 | | 3.20 | |
| Homo sapiens cystathionine-beta-synthase (CBS), mRNA. | CBS | | | 1.32 | | 0 | |
| Homo sapiens Kelch Like Family Member 29 | KLHL29 | | | 1.31 | | 2.27 | |
| Homo sapiens platelet-derived growth factor receptor, alpha polypeptide (PDGFRA), mRNA. | PDGFRA | | | -1.27 | | 3.20 | |
| Homo sapiens serpin peptidase inhibitor, clade F (alpha-2 antiplasmin, pigment epithelium derived factor), member 1 (SERPINF1), mRNA. | SERPINF1 | | | -1.29 | | 3.20 | |
| Homo sapiens phosphoglycerate mutase 2 (muscle) (PGAM2), mRNA. | PGAM2 | | | -1.32 | | 2.44 | |
| Homo sapiens tubulin polymerization-promoting protein family member 3 (TPPP3), mRNA. | TPPP3 | | | -1.36 | | 4.02 | |
| Homo sapiens growth arrest-specific 1 (GAS1), mRNA. | GAS1 | | | -1.38 | | 2.44 | |
| Homo sapiens microtubule-associated protein 1 light chain 3 gamma (MAP1LC3C), mRNA. | MAP1LC3C | | | -1.38 | | 3.20 | |
|  | | | | | | | |
| **Gene Name** | **EtOH vs Cortisol__EE vs EC** | | **Fold Change**  **(EtOH vs Cortisol)** | **q value**  **(EtOH vs Cortisol) (%)** | **Fold Change**  **(EE vs EC)** | **q value**  **(EE vs EC) (%)** | |
| Homo sapiens secreted phosphoprotein 1 (SPP1), transcript variant 2, mRNA. | SPP1 | | 4.16 | 0 | 2.89 | 0 | |
| Homo sapiens chromosome 13 open reading frame 15 (C13orf15), mRNA. | C13ORF15 | | 2.20 | 0 | 1.65 | 0 | |
| Homo sapiens growth arrest and DNA-damage-inducible, alpha (GADD45A), mRNA. | GADD45A | | 1.94 | 0 | 1.75 | 2.83 | |
| Homo sapiens Bardet-Biedl syndrome 2 (BBS2), mRNA. | BBS2 | | 1.87 | 0 | 1.80 | 0 | |
| Homo sapiens zinc finger and BTB domain containing 16 (ZBTB16), transcript variant 2, mRNA. | ZBTB16 | | 1.79 | 0 | 1.60 | 0 | |
| Homo sapiens integrin, alpha 10 (ITGA10), mRNA. | ITGA10 | | 1.78 | 0 | 1.38 | 2.83 | |
| Homo sapiens solute carrier family 4, sodium bicarbonate cotransporter, member 4 (SLC4A4), transcript variant 2, mRNA. | SLC4A4 | | 1.73 | 0 | 1.63 | 2.83 | |
| Homo sapiens immunoglobulin superfamily, member 11 (IGSF11), transcript variant 1, mRNA. | IGSF11 | | 1.67 | 3.93 | 1.43 | 2.83 | |
| Homo sapiens interleukin 11 receptor, alpha (IL11RA), transcript variant 1, mRNA. | IL11RA | | 1.54 | 3.09 | 1.40 | 0 | |
| Homo sapiens glutaredoxin (thioltransferase) (GLRX), mRNA. | GLRX | | -1.32 | 4.66 | -1.34 | 2.05 | |
| Homo sapiens erythrocyte membrane protein band 4.1-like 3 (EPB41L3), mRNA. | EPB41L3 | | -1.32 | 4.66 | -1.37 | 2.05 | |
| Homo sapiens contactin associated protein-like 2 (CNTNAP2), mRNA. | CNTNAP2 | | -1.49 | 0 | -1.38 | 0 | |
| Homo sapiens insulin-like growth factor binding protein 3 (IGFBP3), transcript variant 1, mRNA. | IGFBP3 | | -1.56 | 4.66 | -1.44 | 2.83 | |
|  | | | | | | | |
| **Gene Name** | **EE vs EC__EE vs EEC** | | **Fold Change**  **(EE vs EC)** | **q value**  **(EE vs EC) (%)** | **Fold Change**  **(EE vs EEC)** | **q value**  **(EE vs EEC) (%)** | |
| Homo sapiens family with sequence similarity 107, member A (FAM107A), transcript variant 2, mRNA. | FAM107A | | 1.96 | 0 | 1.57 | 0 | |
| Homo sapiens poly (ADP-ribose) polymerase family, member 8 (PARP8), mRNA. | PARP8 | | 1.66 | 0 | 1.48 | 0 | |
| Homo sapiens 5'-nucleotidase domain containing 3 (NT5DC3), transcript variant 2, mRNA. | NT5DC3 | | 1.53 | 0 | 1.59 | 0 | |
| Homo sapiens bone morphogenetic protein 6 (BMP6), mRNA. | BMP6 | | 1.49 | 0 | 1.80 | 0 | |
| Homo sapiens mucolipin 2 (MCOLN2), mRNA. | MCOLN2 | | 1.44 | 2.83 | 1.57 | 0 | |
| Homo sapiens hemoglobin, theta 1 (HBQ1), mRNA. | HBQ1 | | 1.44 | 2.83 | 1.69 | 0 | |
| Homo sapiens ribosomal protein S7 (RPS7), mRNA. | RPS7 | | 1.40 | 3.66 | 1.37 | 0 | |
| Homo sapiens neural precursor cell expressed, developmentally down-regulated 4-like (NEDD4L), mRNA. | NEDD4L | | -1.25 | 3.66 | -1.30 | 2.44 | |
| Homo sapiens histidine decarboxylase (HDC), mRNA. | HDC | | -1.28 | 4.24 | -1.35 | 3.20 | |
| Homo sapiens SRY (sex determining region Y)-box 11 (SOX11), mRNA. | SOX11 | | -1.29 | 2.05 | -1.34 | 3.20 | |
| Homo sapiens suppressor of cytokine signaling 2 (SOCS2), mRNA. | SOCS2 | | -1.31 | 3.66 | -1.28 | 3.20 | |
| Homo sapiens C1q and tumor necrosis factor related protein 1 (C1QTNF1), mRNA. | C1QTNF1 | | -1.33 | 2.05 | -1.37 | 3.20 | |
| Homo sapiens retinol binding protein 1, cellular (RBP1), mRNA. | RBP1 | | -1.34 | 0 | -1.43 | 0 | |
| Homo sapiens MFNG O-fucosylpeptide 3-beta-N-acetylglucosaminyltransferase (MFNG), mRNA. | MFNG | | -1.35 | 2.05 | -1.42 | 3.20 | |
| Homo sapiens sema domain, seven thrombospondin repeats (type 1 and type 1-like), transmembrane domain (TM) and short cytoplasmic domain, (semaphorin) 5B (SEMA5B), transcript variant 1, mRNA. | SEMA5B | | -1.37 | 2.05 | -1.38 | 2.44 | |
| Homo sapiens delta-like 3 (Drosophila) (DLL3), transcript variant 1, mRNA. | DLL3 | | -1.41 | 2.83 | -1.48 | 3.20 | |
| Homo sapiens synaptotagmin-like 2 (SYTL2), transcript variant b, mRNA. | SYTL2 | | -1.43 | 0 | -1.42 | 3.20 | |
|  | | | | | | | |
| **Gene Name** | **EtOH vs Cortisol__EE vs EEC** | | **Fold Change**  **(EtOH vs Cortisol)** | **q value**  **(EtOH vs Cortisol) (%)** | **Fold Change**  **(EE vs EEC)** | **q value**  **(EE vs EEC) (%)** | |
| Homo sapiens ATP-binding cassette, sub-family C (CFTR/MRP), member 3 (ABCC3), mRNA. | ABCC3 | | 2.11 | 1.66 | 2.36 | 0 | |
| Homo sapiens neuronal cell adhesion molecule (NRCAM), transcript variant 2, mRNA. | NRCAM | | 1.98 | 0 | 1.81 | 3.20 | |
| Homo sapiens sushi-repeat-containing protein, X-linked (SRPX), mRNA. | SRPX | | 1.86 | 1.66 | 1.94 | 3.20 | |
| Homo sapiens phosphodiesterase 8B (PDE8B), transcript variant 3, mRNA. | PDE8B | | 1.62 | 0 | 1.49 | 2.27 | |
| Homo sapiens ribosomal protein, large, P1 (RPLP1), transcript variant 1, mRNA. | PLP1 | | -1.39 | 4.66 | -1.35 | 4.02 | |
|  | | | | | | | |
| **Gene Name** | **All conditions** | **Fold Change (EtOH vs Cortisol)** | **q value (EtOH vs Cortisol) (%)** | **Fold Change**  **(EE vs EC)** | **q value**  **(EE vs EC) (%)** | **Fold Change**  **(EE vs EEC)** | **q value**  **(EE vs EEC) (%)** |
| Homo sapiens DNA (cytosine-5-)-methyltransferase 3 alpha (DNMT3A), transcript variant 2, mRNA. | MT3 | 8.77 | 0 | 9.34 | 0 | 5.90 | 0 |
| Homo sapiens TSC22 domain family, member 3 (TSC22D3), transcript variant 3, mRNA. | TSC22D3 | 5.24 | 0 | 3.72 | 0 | 3.70 | 0 |
| Homo sapiens solute carrier organic anion transporter family, member 2A1 (SLCO2A1), mRNA. | SLCO2A1 | 4.71 | 0 | 4.57 | 0 | 4.14 | 0 |
| Homo sapiens receptor (G protein-coupled) activity modifying protein 1 (RAMP1), mRNA. | RAMP1 | 3.83 | 0 | 4.14 | 0 | 3.78 | 0 |
| Homo sapiens metallothionein 2A (MT2A), mRNA. | MT2A | 3.77 | 0 | 3.75 | 0 | 3.10 | 0 |
| Homo sapiens Kruppel-like factor 9 (KLF9), mRNA. | KLF9 | 3.20 | 0 | 3.02 | 0 | 2.44 | 0 |
| Homo sapiens metallothionein 1A (MT1A), mRNA. | MT1A | 3.10 | 0 | 2.80 | 0 | 2.64 | 0 |
| Homo sapiens aldehyde dehydrogenase 1 family, member L1 (ALDH1L1), mRNA. | ALDH1L1 | 2.85 | 0 | 2.95 | 0 | 2.81 | 0 |
| Homo sapiens neuronal PAS domain protein 1 (NPAS1), mRNA. | NPAS1 | 2.03 | 0 | 1.59 | 4.24 | 2.17 | 0 |
| Homo sapiens fibulin 1 (FBLN1), transcript variant D, mRNA. | FBLN1 | 2.01 | 0 | 1.95 | 0 | 1.70 | 0 |
| Homo sapiens metallothionein 1X (MT1X), mRNA. | MT1X | 1.81 | 0 | 1.95 | 0 | 1.80 | 0 |
| Homo sapiens nebulette (NEBL), transcript variant 2, mRNA. | NEBL | 1.78 | 0 | 1.78 | 0 | 1.75 | 0 |
| Homo sapiens cyclin D3 (CCND3), mRNA. | CCND3 | 1.70 | 0 | 1.73 | 0 | 1.83 | 0 |
| Homo sapiens cannabinoid receptor 1 (brain) (CNR1), transcript variant 1, mRNA. | CNR1 | 1.68 | 0 | 1.54 | 2.83 | 1.76 | 0 |
| Homo sapiens metallothionein 1G (MT1G), mRNA. | MT1G | 1.67 | 0 | 1.66 | 0 | 1.72 | 0 |
| Homo sapiens aldolase C, fructose-bisphosphate (ALDOC), mRNA. | ALDOC | 1.67 | 1.66 | 1.91 | 0 | 1.50 | 3.20 |
| Homo sapiens FK506 binding protein 5 (FKBP5), mRNA. | FKBP5 | 1.65 | 1.66 | 1.99 | 0 | 1.85 | 0 |
| Homo sapiens hemoglobin, alpha 2 (HBA2), mRNA. | HBA2 | 1.59 | 0 | 1.52 | 0 | 2.09 | 0 |
| Homo sapiens plasticity related gene 1 (LPPR4), mRNA. | LPPR4 | -1.40 | 4.66 | -1.42 | 0 | -1.43 | 0 |
| Homo sapiens RALY RNA binding protein-like (RALYL), transcript variant 3, mRNA. | RALYL | -1.44 | 1.97 | -1.40 | 2.83 | -1.45 | 3.20 |
| Homo sapiens collagen, type I, alpha 2 (COL1A2), mRNA. | COL1A2 | -1.44 | 4.66 | -1.30 | 2.05 | -1.44 | 0 |
| Homo sapiens calpain 9 (CAPN9), transcript variant 2, mRNA. | CAPN9 | -1.45 | 4.66 | -1.39 | 0 | -1.42 | 0 |
