# Supplementary material for: The role of omega-3 fatty acids in preventing glucocorticoid-induced reduction in human hippocampal neurogenesis and increase in apoptosis
Source: Transl Psychiatry. 2020 Jul 7;10:219. doi: 10.1038/s41398-020-00908-0 (PMC7341841; doi:10.1038/s41398-020-00908-0)
Supplement: Supplementary file 5 — Table S2 [file 41398_2020_908_MOESM5_ESM.pdf]

Table S2. Signalling pathways modulated by cortisol, EPA alone and by EPA used in pre-treatment, and both pre- and co-treatment with cortisol.

**EtOH vs Cortisol**	
**Pathways**	**Molecules**
Amyotrophic Lateral Sclerosis Signaling	CAPN5,NEFL,NEFM
Germ Cell-Sertoli Cell Junction Signaling	RND3,TUBB,TUBB4B
Remodeling of Epithelial Adherens Junctions	TUBB,TUBB4B
IL-7 Signaling Pathway	BCL6,MYC
Chemokine Signaling	CCL2,CXCR4
Axonal Guidance Signaling	ADAM19,CXCR4,TUBB,TUBB4B
Production of Nitric Oxide and Reactive Oxygen Species in Macrophages	RND3
mTOR Signaling	RND3
Phosphatidylcholine Biosynthesis I	CHPT1
Leucine Degradation I	BCAT2
GP6 Signaling Pathway	COL23A1,LAMB1
Atherosclerosis Signaling	CCL2,CXCR4
14-3-3-mediated Signaling	TUBB,TUBB4B
Isoleucine Degradation I	BCAT2
Choline Biosynthesis III	CHPT1
Phagosome Maturation	TUBB,TUBB4B
Parkinson's Signaling	SNCAIP
Epithelial Adherens Junction Signaling	TUBB,TUBB4B
Differential Regulation of Cytokine Production in Macrophages and T Helper Cells by IL-17A and IL-17F	CCL2
Valine Degradation I	BCAT2
HMGB1 Signaling	CCL2,RND3
Glioblastoma Multiforme Signaling	MYC,RND3
CXCR4 Signaling	CXCR4,RND3
Granulocyte Adhesion and Diapedesis	CCL2,CXCR4
Polyamine Regulation in Colon Cancer	MYC
Differential Regulation of Cytokine Production in Intestinal Epithelial Cells by IL-17A and IL-17F	CCL2
Agranulocyte Adhesion and Diapedesis	CCL2,CXCR4
Sertoli Cell-Sertoli Cell Junction Signaling	TUBB,TUBB4B
Hepatic Fibrosis / Hepatic Stellate Cell Activation	CCL2,COL23A1

Estrogen-mediated S-phase Entry	MYC
ILK Signaling	MYC,RND3
ERK/MAPK Signaling	MYC,MYCN
**EtOH vs EE**	
**Pathways**	**Molecules**
Adrenomedullin signaling pathway	ADM
PPAR signaling pathway	ANGPTL4
**EE vs EC**	
**Pathways**	**Molecules**
Glioblastoma Multiforme Signaling	PDGFA,SRC,TCF3
AMPK Signaling	PPP2R2B,SMARCB1,SRC
Macropinocytosis Signaling	PDGFA,SRC
CXCR4 Signaling	ELMO1,SRC
PDGF Signaling	PDGFA,SRC
NRF2-mediated Oxidative Stress Response	DNAJB6
Glucocorticoid Receptor Signaling	SMARCB1
Citrulline-Nitric Oxide Cycle	ASS1
Role of Macrophages, Fibroblasts and Endothelial Cells in Rheumatoid Arthritis	PDGFA,SRC,TCF3
Arginine Biosynthesis IV	ASS1
Urea Cycle	ASS1
Virus Entry via Endocytic Pathways	CXADR,SRC
p70S6K Signaling	PPP2R2B,SRC
Calcium Transport I	ATP2B4
Human Embryonic Stem Cell Pluripotency	PDGFA,TCF3
Ovarian Cancer Signaling	SRC,TCF3
Endocannabinoid Cancer Inhibition Pathway	SRC,TCF3
Epithelial Adherens Junction Signaling	SRC,TCF3
Superpathway of Citrulline Metabolism	ASS1
Ephrin Receptor Signaling	PDGFA,SRC
RAR Activation	SMARCB1,SRC
Clathrin-mediated Endocytosis Signaling	PDGFA,SRC
ERK/MAPK Signaling	PPP2R2B,SRC

Role of Osteoblasts, Osteoclasts and Chondrocytes in Rheumatoid Arthritis	SRC,TCF3
Colorectal Cancer Metastasis Signaling	SRC,TCF3
Cell Cycle Regulation by BTG Family Proteins	PPP2R2B
**EE vs EEC**	
**Pathways**	**Molecules**
Mitotic Roles of Polo-Like Kinase	ANAPC1
STAT3 Pathway	PDGFRA
NF-κB Signaling	PDGFRA
Cysteine Biosynthesis/Homocysteine Degradation	CBS/CBSL
GDP-L-fucose Biosynthesis I (from GDP-D-mannose)	GMDS
Rapoport-Luebering Glycolytic Shunt	PGAM2
IL-15 Production	MATK,PDGFRA
Colanic Acid Building Blocks Biosynthesis	GMDS
Glutathione Redox Reactions I	MGST1
Cysteine Biosynthesis III (mammalia)	CBS/CBSL
Glycolysis I	PGAM2
Glutathione-mediated Detoxification	MGST1
Gluconeogenesis I	PGAM2
Superpathway of Methionine Degradation	CBS/CBSL
Sirtuin Signaling Pathway	MAP1LC3C,PGAM2
Docosahexaenoic Acid (DHA) Signaling	SERPINF1
**EtOH vs Cortisol_EE vs EC**	
**Pathways**	**Molecules**
VDR/RXR Activation	GADD45A,IGFBP3,SPP1
Ascorbate Recycling (Cytosolic)	GLRX
Glutathione Redox Reactions II	GLRX
RAR Activation	IGFBP3,ZBTB16
Vitamin-C Transport	GLRX
GADD45 Signaling	GADD45A
Role of Oct4 in Mammalian Embryonic Stem Cell Pluripotency	SPP1
Cell Cycle: G2/M DNA Damage Checkpoint Regulation	GADD45A
Growth Hormone Signaling	IGFBP3

Caveolar-mediated Endocytosis Signaling	ITGA10
Role of BRCA1 in DNA Damage Response	GADD45A
Altered T Cell and B Cell Signaling in Rheumatoid Arthritis	SPP1
**EtOH vs Cortisol__EE vs EEC**	
**Pathways**	**Molecules**
tRNA Splicing	PDE8B
PXR/RXR Activation	ABCC3
Cardiac β-adrenergic Signaling	PDE8B
Relaxin Signaling	PDE8B
Gustation Pathway	PDE8B
**EE vs EC__EE vs EEC**	
**Pathways**	**Molecules**
Histamine Biosynthesis	HDC
Protein Ubiquitination Pathway	NEDD4L
Retinoate Biosynthesis II	RBP1
Iron homeostasis signaling pathway	BMP6,HBQ1
STAT3 Pathway	BMP6,SOCS2
Acute Phase Response Signaling	RBP1,SOCS2
The Visual Cycle	RBP1
Retinoate Biosynthesis I	RBP1
Role of JAK2 in Hormone-like Cytokine Signaling	SOCS2
IL-9 Signaling	SOCS2
Notch Signaling	DLL3,MFNG
Retinol Biosynthesis	RBP1
Retinoic acid Mediated Apoptosis Signaling	PARP8
**All conditions**	
**Pathways**	**Molecules**
Cyclins and Cell Cycle Regulation	CCND3
SPINK1 General Cancer Pathway	MT1A,MT1G,MT1X,MT2A,MT3
Sucrose Degradation V (Mammalian)	ALDOC
Aryl Hydrocarbon Receptor Signaling	ALDH1L1,CCND3

Endocannabinoid Cancer Inhibition Pathway	CCND3,CNR1
GADD45 Signaling	CCND3
Glycolysis I	ALDOC
Gluconeogenesis I	ALDOC
Apelin Liver Signaling Pathway	COL1A2
Intrinsic Prothrombin Activation Pathway	COL1A2
Oncostatin M Signaling	MT2A
Glucocorticoid Receptor Signaling	FKBP5,TSC22D3
nNOS Signaling in Neurons	CAPN9
