# Supplementary material for: The role of omega-3 fatty acids in preventing glucocorticoid-induced reduction in human hippocampal neurogenesis and increase in apoptosis
Source: Transl Psychiatry. 2020 Jul 7;10:219. doi: 10.1038/s41398-020-00908-0 (PMC7341841; doi:10.1038/s41398-020-00908-0)
Supplement: Supplementary file 6 — Table S3 [file 41398_2020_908_MOESM6_ESM.docx]

**Table S3.** Genes regulated by cortisol, DHA alone and by DHA in pre-treatment, and both pre- and co-treatment with cortisol.

| **Gene Name** | | | **EtOH vs Cortisol** | | | **Fold Change** | | | | | | | **q value (%)** | |
| --- | --- | --- | --- | --- | --- | --- | --- | --- | --- | --- | --- | --- | --- | --- |
| Homo sapiens small nucleolar RNA, C/D box 3C (SNORD3C), small nucleolar RNA. | | | SNORD3C | | | 1.60 | | | | | | | 1.66 | |
| Homo sapiens laminin, beta 1 (LAMB1), mRNA. | | | LAMB1 | | | 1.49 | | | | | | | 3.93 | |
| Homo sapiens kelch repeat and BTB (POZ) domain containing 11 (KBTBD11), mRNA. | | | KBTBD11 | | | 1.42 | | | | | | | 3.93 | |
| Homo sapiens transmembrane protein 30A (TMEM30A), mRNA. | | | TMEM30A | | | 1.40 | | | | | | | 3.93 | |
| Homo sapiens protease, serine, 23 (PRSS23), mRNA. | | | PRSS23 | | | -1.40 | | | | | | | 0 | |
|  | | | | | | | | | | | | | | |
| **Gene Name** | | | **EtOH vs DD** | | | **Fold Change** | | | | | | | **q value (%)** | |
| Homo sapiens angiopoietin-like 4 (ANGPTL4), transcript variant 1, mRNA. | | | ANGPTL4 | | | 4.54 | | | | | | | 0 | |
| Homo sapiens pyruvate dehydrogenase kinase, isozyme 4 (PDK4), mRNA. | | | PDK4 | | | 2.26 | | | | | | | 0 | |
| Homo sapiens glutathione peroxidase 3 (plasma) (GPX3), mRNA. | | | GPX3 | | | 2.08 | | | | | | | 0 | |
| Homo sapiens seizure related 6 homolog (mouse) (SEZ6), transcript variant 2, mRNA. | | | SEZ6 | | | 1.83 | | | | | | | 3.46 | |
| Homo sapiens carnitine palmitoyltransferase 1A (liver) (CPT1A), nuclear gene encoding mitochondrial protein, transcript variant 1, mRNA. | | | CPT1A | | | 1.66 | | | | | | | 3.46 | |
| Homo sapiens adrenomedullin (ADM), mRNA. | | | ADM | | | 1.59 | | | | | | | 0 | |
| Homo sapiens monocyte to macrophage differentiation-associated (MMD), mRNA. | | | MMD | | | 1.51 | | | | | | | 0 | |
| Homo sapiens cadherin 6, type 2, K-cadherin (fetal kidney) (CDH6), mRNA. | | | CDH6 | | | 1.44 | | | | | | | 3.46 | |
| Homo sapiens synuclein, alpha (non A4 component of amyloid precursor) (SNCA), transcript variant NACP140, mRNA. | | | SNCA | | | -1.27 | | | | | | | 4.21 | |
| Homo sapiens family with sequence similarity 69, member B (FAM69B), mRNA. | | | FAM69B | | | -1.28 | | | | | | | 4.21 | |
| Homo sapiens GrpE-like 1, mitochondrial (E. coli) (GRPEL1), nuclear gene encoding mitochondrial protein, mRNA. | | | GRPEL1 | | | -1.29 | | | | | | | 4.21 | |
| Homo sapiens Thy-1 cell surface antigen (THY1), mRNA. | | | THY1 | | | -1.34 | | | | | | | 4.21 | |
| Homo sapiens 3-hydroxy-3-methylglutaryl-Coenzyme A synthase 1 (soluble) (HMGCS1), transcript variant 2, mRNA. | | | HMGCS1 | | | -1.34 | | | | | | | 0 | |
| Homo sapiens lipin 1 (LPIN1), mRNA. | | | LPIN1 | | | -1.35 | | | | | | | 0 | |
| Homo sapiens aldolase C, fructose-bisphosphate (ALDOC), mRNA. | | | ALDOC | | | -1.35 | | | | | | | 0 | |
| Homo sapiens eyes absent homolog 4 (Drosophila) (EYA4), transcript variant 4, mRNA. | | | EYA4 | | | -1.37 | | | | | | | 0 | |
| Homo sapiens glypican 2 (GPC2), mRNA. | | | GPC2 | | | -1.37 | | | | | | | 0 | |
| Homo sapiens folate receptor 1 (adult) (FOLR1), transcript variant 1, mRNA. | | | FOLR1 | | | -1.43 | | | | | | | 0 | |
| Homo sapiens collagen, type VIII, alpha 2 (COL8A2), mRNA. | | | COL8A2 | | | -1.47 | | | | | | | 0 | |
| Homo sapiens phospholipase A2, group III (PLA2G3), mRNA. | | | PLA2G3 | | | -1.49 | | | | | | | 0 | |
| Homo sapiens sterol regulatory element binding transcription factor 1 (SREBF1), transcript variant 1, mRNA. | | | SREBF1 | | | -1.63 | | | | | | | 0 | |
| Homo sapiens insulin-like growth factor binding protein 5 (IGFBP5), mRNA. | | | IGFBP5 | | | -1.63 | | | | | | | 0 | |
|  | | | | | | | | | | | | | | |
| **Gene Name** | | | **DD vs DC** | | | **Fold Change** | | | | | | | **q value (%)** | |
| Homo sapiens chromosome 15 open reading frame 59 (C15orf59), mRNA. | | | C15ORF59 | | | 1.62 | | | | | | | 0 | |
| Homo sapiens centaurin, alpha 1 (CENTA1), mRNA. | | | CENTA1 | | | 1.45 | | | | | | | 1.27 | |
| Homo sapiens pyruvate carboxylase (PC), nuclear gene encoding mitochondrial protein, transcript variant A, mRNA. | | | PC | | | 1.42 | | | | | | | 0 | |
| Homo sapiens scavenger receptor class B, member 1 (SCARB1), mRNA. | | | SCARB1 | | | 1.42 | | | | | | | 0.74 | |
| Homo sapiens coiled-coil domain containing 28B (CCDC28B), mRNA. | | | CCDC28B | | | 1.37 | | | | | | | 2.46 | |
| Homo sapiens U2 small nuclear RNA auxiliary factor 1 (U2AF1), transcript variant b, mRNA. | | | U2AF1 | | | 1.37 | | | | | | | 0.74 | |
| Homo sapiens chromosome 1 open reading frame 43 (C1orf43), transcript variant 1, mRNA. | | | C1ORF43 | | | 1.37 | | | | | | | 0.74 | |
| Homo sapiens sperm associated antigen 7 (SPAG7), mRNA. | | | SPAG7 | | | 1.35 | | | | | | | 2.46 | |
| Homo sapiens chromosome 6 open reading frame 108 (C6orf108), transcript variant 2, mRNA. | | | C6ORF108 | | | 1.30 | | | | | | | 1.27 | |
| Homo sapiens fibroblast growth factor receptor 3 (achondroplasia, thanatophoric dwarfism) (FGFR3), transcript variant 1, mRNA. | | | FGFR3 | | | 1.22 | | | | | | | 2.36 | |
| Homo sapiens 6-phosphofructo-2-kinase/fructose-2,6-biphosphatase 3 (PFKFB3), mRNA. | | | PFKFB3 | | | 1.21 | | | | | | | 1.69 | |
| Homo sapiens armadillo repeat containing 3 (ARMC3), mRNA. | | | ARMC3 | | | -1.23 | | | | | | | 2.46 | |
| Homo sapiens family with sequence similarity 176, member A (FAM176A), transcript variant 1, mRNA. | | | FAM176A | | | -1.24 | | | | | | | 0.74 | |
| Homo sapiens C1q and tumor necrosis factor related protein 6 (C1QTNF6), transcript variant 2, mRNA. | | | C1QTNF6 | | | -1.24 | | | | | | | 1.27 | |
| Homo sapiens chromosome 2 open reading frame 39 (C2orf39), mRNA. | | | C2ORF39 | | | -1.26 | | | | | | | 1.69 | |
| Homo sapiens shroom (SHRM), mRNA. | | | SHRM | | | -1.26 | | | | | | | 2.46 | |
| Homo sapiens transmembrane protein 163 (TMEM163), mRNA. | | | TMEM163 | | | -1.26 | | | | | | | 2.46 | |
| Homo sapiens collagen, type XI, alpha 1 (COL11A1), transcript variant A, mRNA. | | | COL11A1 | | | -1.27 | | | | | | | 0.74 | |
| Homo sapiens coagulation factor II (thrombin) receptor (F2R), mRNA. | | | F2R | | | -1.27 | | | | | | | 1.27 | |
| Homo sapiens SLIT-ROBO Rho GTPase activating protein 3 (SRGAP3), transcript variant 1, mRNA. | | | SRGAP3 | | | -1.28 | | | | | | | 1.69 | |
| Homo sapiens matrix metallopeptidase 15 (membrane-inserted) (MMP15), mRNA. | | | MMP15 | | | -1.28 | | | | | | | 1.69 | |
| Homo sapiens transcription factor AP-2 gamma (activating enhancer binding protein 2 gamma) (TFAP2C), mRNA. | | | TFAP2C | | | -1.29 | | | | | | | 1.27 | |
| Homo sapiens glutaminyl-peptide cyclotransferase (QPCT), mRNA. | | | QPCT | | | -1.29 | | | | | | | 0 | |
| Homo sapiens chromosome 1 open reading frame 88 (C1orf88), mRNA. | | | C1ORF88 | | | -1.29 | | | | | | | 0 | |
| Homo sapiens transmembrane protein 98 (TMEM98), transcript variant 2, mRNA. | | | TMEM98 | | | -1.29 | | | | | | | 1.27 | |
| Homo sapiens sel-1 suppressor of lin-12-like 3 (C. elegans) (SEL1L3), mRNA. | | | SEL1L3 | | | -1.29 | | | | | | | 0 | |
| Homo sapiens adducin 3 (gamma) (ADD3), transcript variant 1, mRNA. | | | ADD3 | | | -1.29 | | | | | | | 1.69 | |
| Homo sapiens frizzled-related protein (FRZB), mRNA. | | | FRZB | | | -1.30 | | | | | | | 1.27 | |
| Homo sapiens syndecan 2 (SDC2), mRNA. | | | SDC2 | | | -1.30 | | | | | | | 0 | |
| Homo sapiens family with sequence similarity 164, member A (FAM164A), mRNA. | | | FAM164A | | | -1.31 | | | | | | | 1.27 | |
| Homo sapiens growth arrest-specific 1 (GAS1), mRNA. | | | GAS1 | | | -1.31 | | | | | | | 0 | |
| Homo sapiens cell adhesion molecule 4 (CADM4), mRNA. | | | CADM4 | | | -1.31 | | | | | | | 0 | |
| Homo sapiens ubiquitin-conjugating enzyme E2H (UBC8 homolog, yeast) (UBE2H), transcript variant 1, mRNA. | | | UBE2H | | | -1.31 | | | | | | | 1.27 | |
| Homo sapiens interferon induced transmembrane protein 3 (1-8U) (IFITM3), mRNA. | | | IFITM3 | | | -1.31 | | | | | | | 1.69 | |
| Homo sapiens acetyl-Coenzyme A acyltransferase 2 (mitochondrial 3-oxoacyl-Coenzyme A thiolase) (ACAA2), nuclear gene encoding mitochondrial protein, mRNA. | | | ACAA2 | | | -1.31 | | | | | | | 2.46 | |
| Homo sapiens bromodomain containing 8 (BRD8), transcript variant 2, mRNA. | | | BRD8 | | | -1.32 | | | | | | | 0 | |
| Homo sapiens sortilin 1 (SORT1), mRNA. | | | SORT1 | | | -1.32 | | | | | | | 0.74 | |
| Homo sapiens stonin 1 (STON1), mRNA. | | | STON1 | | | -1.32 | | | | | | | 0.74 | |
| Homo sapiens potassium inwardly-rectifying channel, subfamily J, member 2 (KCNJ2), mRNA. | | | KCNJ2 | | | -1.32 | | | | | | | 2.46 | |
| Homo sapiens fibronectin leucine rich transmembrane protein 3 (FLRT3), transcript variant 2, mRNA. | | | FLRT3 | | | -1.34 | | | | | | | 0 | |
| Homo sapiens butyrobetaine (gamma), 2-oxoglutarate dioxygenase (gamma-butyrobetaine hydroxylase) 1 (BBOX1), mRNA. | | | BBOX1 | | | -1.35 | | | | | | | 0 | |
| Homo sapiens thrombospondin 1 (THBS1), mRNA. | | | THBS1 | | | -1.35 | | | | | | | 0 | |
| Homo sapiens major histocompatibility complex, class II, DR alpha (HLA-DRA), mRNA. | | | HLA-DRA | | | -1.36 | | | | | | | 0 | |
| Homo sapiens cytoplasmic polyadenylation element binding protein 4 (CPEB4), mRNA. | | | CPEB4 | | | -1.36 | | | | | | | 0 | |
| Homo sapiens podoplanin (PDPN), transcript variant 2, mRNA. | | | PDPN | | | -1.37 | | | | | | | 0 | |
| Homo sapiens limb bud and heart development homolog (mouse) (LBH), mRNA. | | | LBH | | | -1.40 | | | | | | | 0 | |
| Homo sapiens tropomyosin 1 (alpha) (TPM1), transcript variant 6, mRNA. | | | TPM1 | | | -1.41 | | | | | | | 0 | |
| Homo sapiens transmembrane 4 L six family member 1 (TM4SF1), mRNA. | | | TM4SF1 | | | -1.50 | | | | | | | 0 | |
| Homo sapiens secreted frizzled-related protein 4 (SFRP4), mRNA. | | | SFRP4 | | | -1.50 | | | | | | | 0 | |
| Homo sapiens transgelin (TAGLN), transcript variant 2, mRNA. | | | TAGLN | | | -1.54 | | | | | | | 0 | |
| Homo sapiens cysteine-rich, angiogenic inducer, 61 (CYR61), mRNA. | | | CYR61 | | | -1.56 | | | | | | | 0 | |
|  | | | | | | | | | | | | | | |
| **Gene Name** | | | **DD vs DDC** | | | **Fold Change** | | | | | | | **q value (%)** | |
| Homo sapiens epithelial membrane protein 1 (EMP1), mRNA. | | | EMP1 | | | 3.30 | | | | | | | 0 | |
| Homo sapiens serpin peptidase inhibitor, clade E (nexin, plasminogen activator inhibitor type 1), member 1 (SERPINE1), mRNA. | | | SERPINE1 | | | 2.76 | | | | | | | 0 | |
| Homo sapiens glutathione peroxidase 3 (plasma) (GPX3), mRNA. | | | GPX3 | | | 2.73 | | | | | | | 0 | |
| Homo sapiens growth differentiation factor 15 (GDF15), mRNA. | | | GDF15 | | | 2.46 | | | | | | | 0 | |
| Homo sapiens potassium voltage-gated channel, subfamily F, member 1 (KCNF1), mRNA. | | | KCNF1 | | | 2.41 | | | | | | | 0 | |
| Homo sapiens AHNAK Nucleoprotein 2 | | | C14ORF78 | | | 2.37 | | | | | | | 0 | |
| Homo sapiens AHNAK nucleoprotein 2 (AHNAK2), mRNA. | | | AHNAK2 | | | 2.20 | | | | | | | 0 | |
| Homo sapiens serpin peptidase inhibitor, clade A (alpha-1 antiproteinase, antitrypsin), member 3 (SERPINA3), mRNA. | | | SERPINA3 | | | 2.19 | | | | | | | 0 | |
| Homo sapiens serum/glucocorticoid regulated kinase (SGK), mRNA. | | | SGK | | | 2.16 | | | | | | | 0 | |
| Homo sapiens crystallin, alpha B (CRYAB), mRNA. | | | CRYAB | | | 2.15 | | | | | | | 0 | |
| Homo sapiens insulin-like growth factor binding protein 7 (IGFBP7), mRNA. | | | IGFBP7 | | | 2.05 | | | | | | | 0 | |
| Homo sapiens adenomatosis polyposis coli down-regulated 1-like (APCDD1L), mRNA. | | | APCDD1L | | | 1.97 | | | | | | | 0 | |
| Homo sapiens integrin, beta 4 (ITGB4), transcript variant 3, mRNA. | | | ITGB4 | | | 1.94 | | | | | | | 0 | |
| Homo sapiens fer-1-like 3, myoferlin (C. elegans) (FER1L3), transcript variant 1, mRNA. | | | FER1L3 | | | 1.94 | | | | | | | 0 | |
| Homo sapiens secretogranin II (chromogranin C) (SCG2), mRNA. | | | SCG2 | | | 1.94 | | | | | | | 0 | |
| Homo sapiens immediate early response 3 (IER3), mRNA. | | | IER3 | | | 1.89 | | | | | | | 0 | |
| Homo sapiens pyruvate dehydrogenase kinase, isozyme 4 (PDK4), mRNA. | | | PDK4 | | | 1.88 | | | | | | | 0 | |
| Homo sapiens serum/glucocorticoid regulated kinase 1 (SGK1), transcript variant 1, mRNA. | | | SGK1 | | | 1.88 | | | | | | | 0 | |
| Homo sapiens maternally expressed 3 (non-protein coding) (MEG3), transcript variant 1, non-coding RNA. XR_001346-XR_001372 | | | MEG3 | | | 1.85 | | | | | | | 0 | |
| Homo sapiens ferritin, heavy polypeptide-like 8 (FTHL8) on chromosome X. | | | FTHL8 | | | 1.82 | | | | | | | 0 | |
| Homo sapiens LIM domain only 2 (rhombotin-like 1) (LMO2), mRNA. | | | LMO2 | | | 1.78 | | | | | | | 0 | |
| Homo sapiens caveolin 1, caveolae protein, 22kDa (CAV1), mRNA. | | | CAV1 | | | 1.77 | | | | | | | 0 | |
| Homo sapiens transforming growth factor, beta-induced, 68kDa (TGFBI), mRNA. | | | TGFBI | | | 1.75 | | | | | | | 0 | |
| Homo sapiens inhibitor of DNA binding 1, dominant negative helix-loop-helix protein (ID1), transcript variant 2, mRNA. | | | ID1 | | | 1.75 | | | | | | | 0 | |
| Homo sapiens melanoma antigen family D, 2 (MAGED2), transcript variant 1, mRNA. | | | MAGED2 | | | 1.74 | | | | | | | 0 | |
| Homo sapiens myelin basic protein (MBP), transcript variant 7, mRNA. | | | MBP | | | 1.72 | | | | | | | 0 | |
| Homo sapiens c-mer proto-oncogene tyrosine kinase (MERTK), mRNA. | | | MERTK | | | 1.70 | | | | | | | 0 | |
| Homo sapiens A kinase (PRKA) anchor protein (gravin) 12 (AKAP12), transcript variant 2, mRNA. | | | AKAP12 | | | 1.69 | | | | | | | 0 | |
| Homo sapiens cholinergic receptor, nicotinic, alpha 9 (CHRNA9), mRNA. | | | CHRNA9 | | | 1.69 | | | | | | | 0 | |
| Homo sapiens myoferlin (MYOF), transcript variant 1, mRNA. | | | MYOF | | | 1.67 | | | | | | | 0 | |
| Homo sapiens GTP cyclohydrolase I feedback regulator (GCHFR), mRNA. | | | GCHFR | | | 1.67 | | | | | | | 0 | |
| Homo sapiens latent transforming growth factor beta binding protein 2 (LTBP2), mRNA. | | | LTBP2 | | | 1.66 | | | | | | | 0.34 | |
| Homo sapiens LON peptidase N-terminal domain and ring finger 2 (LONRF2), mRNA. | | | LONRF2 | | | 1.65 | | | | | | | 0.34 | |
| Homo sapiens CCAAT/enhancer binding protein (C/EBP), delta (CEBPD), mRNA. | | | CEBPD | | | 1.64 | | | | | | | 0 | |
| Homo sapiens sterile alpha motif domain containing 11 (SAMD11), mRNA. | | | SAMD11 | | | 1.64 | | | | | | | 0.34 | |
| Homo sapiens NK6 homeobox 2 (NKX6-2), mRNA. | | | NKX6-2 | | | 1.64 | | | | | | | 0.34 | |
| Homo sapiens SPOC domain containing 1 (SPOCD1), mRNA. | | | SPOCD1 | | | 1.64 | | | | | | | 0.34 | |
| Homo sapiens Ankyrin Repeat Domain 36B | | | KIAA1641 | | | 1.63 | | | | | | | 0 | |
| Homo sapiens calcium/calmodulin-dependent protein kinase (CaM kinase) II delta (CAMK2D), transcript variant 4, mRNA. | | | CAMK2D | | | 1.63 | | | | | | | 0.34 | |
| Homo sapiens transforming growth factor, beta receptor II (70/80kDa) (TGFBR2), transcript variant 1, mRNA. | | | TGFBR2 | | | 1.62 | | | | | | | 0 | |
| Homo sapiens serpin peptidase inhibitor, clade E (nexin, plasminogen activator inhibitor type 1), member 2 (SERPINE2), mRNA. | | | SERPINE2 | | | 1.61 | | | | | | | 0.34 | |
| Homo sapiens gelsolin (amyloidosis, Finnish type) (GSN), transcript variant 2, mRNA. | | | GSN | | | 1.61 | | | | | | | 0.34 | |
| Homo sapiens microtubule-associated protein tau (MAPT), transcript variant 1, mRNA. | | | MAPT | | | 1.60 | | | | | | | 0 | |
| Homo sapiens progestin and adipoQ receptor family member VIII (PAQR8), mRNA. | | | PAQR8 | | | 1.60 | | | | | | | 0 | |
| Homo sapiens chromosome 16 open reading frame 73 (C16orf73), mRNA. | | | C16ORF73 | | | 1.59 | | | | | | | 0.34 | |
| Homo sapiens solute carrier family 47, member 2 (SLC47A2), transcript variant 1, mRNA. | | | SLC47A2 | | | 1.58 | | | | | | | 0.70 | |
| Homo sapiens platelet-derived growth factor alpha polypeptide (PDGFA), transcript variant 2, mRNA. | | | PDGFA | | | 1.58 | | | | | | | 0.53 | |
| Homo sapiens collagen, type VIII, alpha 2 (COL8A2), mRNA. | | | COL8A2 | | | 1.58 | | | | | | | 0.34 | |
| Homo sapiens perilipin 2 (PLIN2), mRNA. | | | PLIN2 | | | 1.58 | | | | | | | 0 | |
| Homo sapiens RAS-like, family 11, member B (RASL11B), mRNA. | | | RASL11B | | | 1.58 | | | | | | | 0.34 | |
| Homo sapiens acyl-CoA synthetase short-chain family member 1 (ACSS1), nuclear gene encoding mitochondrial protein, mRNA. | | | ACSS1 | | | 1.57 | | | | | | | 0.34 | |
| Homo sapiens neuronal pentraxin I (NPTX1), mRNA. | | | NPTX1 | | | 1.57 | | | | | | | 0 | |
| Homo sapiens phosphatidylinositol-5-phosphate 4-kinase, type II, alpha (PIP4K2A), mRNA. | | | PIP4K2A | | | 1.57 | | | | | | | 0 | |
| Homo sapiens pleckstrin homology-like domain, family A, member 2 (PHLDA2), mRNA. | | | PHLDA2 | | | 1.56 | | | | | | | 0.34 | |
| Homo sapiens adenylosuccinate synthase (ADSS), mRNA. | | | ADSS | | | 1.56 | | | | | | | 0 | |
| Homo sapiens coagulation factor XII (Hageman factor) (F12), mRNA. | | | F12 | | | 1.56 | | | | | | | 0.34 | |
| Homo sapiens tumor protein D52-like 1 (TPD52L1), transcript variant 3, mRNA. | | | TPD52L1 | | | 1.55 | | | | | | | 0 | |
| Homo sapiens lectin, galactoside-binding, soluble, 3 (galectin 3) (LGALS3), mRNA. | | | LGALS3 | | | 1.55 | | | | | | | 0.53 | |
| Homo sapiens renin binding protein (RENBP), mRNA. | | | RENBP | | | 1.55 | | | | | | | 0.34 | |
| Homo sapiens small nucleolar RNA, C/D box 114-3 (SNORD114-3), small nucleolar RNA. | | | SNORD114-3 | | | 1.55 | | | | | | | 0.34 | |
| Homo sapiens caprin family member 2 (CAPRIN2), transcript variant 1, mRNA. | | | CAPRIN2 | | | 1.54 | | | | | | | 0.34 | |
| Homo sapiens ribosomal protein S6 kinase, 90kDa, polypeptide 2 (RPS6KA2), transcript variant 1, mRNA. | | | RPS6KA2 | | | 1.54 | | | | | | | 0.34 | |
| Homo sapiens integrin, alpha 3 (antigen CD49C, alpha 3 subunit of VLA-3 receptor) (ITGA3), transcript variant a, mRNA. | | | ITGA3 | | | 1.54 | | | | | | | 0.70 | |
| Homo sapiens complement component 1, q subcomponent-like 1 (C1QL1), mRNA. | | | C1QL1 | | | 1.54 | | | | | | | 0.34 | |
| Homo sapiens bromodomain and WD repeat domain containing 1 (BRWD1), transcript variant 3, mRNA. | | | BRWD1 | | | 1.52 | | | | | | | 0.34 | |
| Homo sapiens phosphorylase, glycogen; brain (PYGB), mRNA. | | | PYGB | | | 1.51 | | | | | | | 0.34 | |
| Homo sapiens glial fibrillary acidic protein (GFAP), mRNA. | | | GFAP | | | 1.51 | | | | | | | 0.53 | |
| Homo sapiens pregnancy upregulated non-ubiquitously expressed CaM kinase (PNCK), mRNA. | | | PNCK | | | 1.51 | | | | | | | 0 | |
| Homo sapiens family with sequence similarity 43, member A (FAM43A), mRNA. | | | FAM43A | | | 1.51 | | | | | | | 0.34 | |
| Homo sapiens dual specificity phosphatase 1 (DUSP1), mRNA. | | | DUSP1 | | | 1.51 | | | | | | | 0.34 | |
| Homo sapiens collagen, type VIII, alpha 1 (COL8A1), transcript variant 1, mRNA. | | | COL8A1 | | | 1.51 | | | | | | | 0.34 | |
| Homo sapiens F-box protein 32 (FBXO32), transcript variant 1, mRNA. | | | FBXO32 | | | 1.51 | | | | | | | 0.53 | |
| Homo sapiens eukaryotic translation elongation factor 1 alpha 2 (EEF1A2), mRNA. | | | EEF1A2 | | | 1.50 | | | | | | | 0.34 | |
| Homo sapiens WW domain containing E3 ubiquitin protein ligase 1 (WWP1), mRNA. | | | WWP1 | | | 1.50 | | | | | | | 0.34 | |
| Homo sapiens ferritin, heavy polypeptide-like 2 (FTHL2) on chromosome 1. | | | FTHL2 | | | 1.49 | | | | | | | 0.53 | |
| Homo sapiens Kv channel interacting protein 1 (KCNIP1), transcript variant 3, mRNA. | | | KCNIP1 | | | 1.49 | | | | | | | 0.34 | |
| Homo sapiens AXL receptor tyrosine kinase (AXL), transcript variant 1, mRNA. | | | AXL | | | 1.49 | | | | | | | 0.34 | |
| Homo sapiens transcription factor B1, mitochondrial (TFB1M), mRNA. | | | TFB1M | | | 1.49 | | | | | | | 0.53 | |
| Homo sapiens SEC14-like 1 (S. cerevisiae) (SEC14L1), transcript variant 1, mRNA. | | | SEC14L1 | | | 1.49 | | | | | | | 0.34 | |
| Homo sapiens transmembrane protein 120A (TMEM120A), mRNA. | | | TMEM120A | | | 1.49 | | | | | | | 0.34 | |
| Homo sapiens transmembrane protein 16A (TMEM16A), mRNA. | | | TMEM16A | | | 1.49 | | | | | | | 0.34 | |
| Homo sapiens myotubularin related protein 11 (MTMR11), mRNA. | | | MTMR11 | | | 1.49 | | | | | | | 0.34 | |
| Homo sapiens myosin IE (MYO1E), mRNA. | | | MYO1E | | | 1.49 | | | | | | | 0.34 | |
| Homo sapiens chromosome 6 open reading frame 15 (C6orf15), mRNA. | | | C6ORF15 | | | 1.49 | | | | | | | 0.70 | |
| Homo sapiens spermidine/spermine N1-acetyltransferase 1 (SAT1), mRNA. | | | SAT1 | | | 1.48 | | | | | | | 0.34 | |
| Homo sapiens CD24 molecule (CD24), mRNA. | | | CD24 | | | 1.48 | | | | | | | 0.99 | |
| Homo sapiens pyruvate dehydrogenase (lipoamide) alpha 1 (PDHA1), mRNA. | | | PDHA1 | | | 1.48 | | | | | | | 0.53 | |
| Homo sapiens solute carrier organic anion transporter family, member 3A1 (SLCO3A1), mRNA. | | | SLCO3A1 | | | 1.48 | | | | | | | 0.34 | |
| Homo sapiens methyltransferase like 7B (METTL7B), mRNA. | | | METTL7B | | | 1.47 | | | | | | | 0.53 | |
| Homo sapiens solute carrier family 4, sodium bicarbonate cotransporter, member 7 (SLC4A7), mRNA. | | | SLC4A7 | | | 1.47 | | | | | | | 0.34 | |
| Homo sapiens hemoglobin, theta 1 (HBQ1), mRNA. | | | HBQ1 | | | 1.47 | | | | | | | 1.05 | |
| Homo sapiens leucine rich repeat containing 32 (LRRC32), mRNA. | | | LRRC32 | | | 1.46 | | | | | | | 0.53 | |
| Homo sapiens zinc finger protein 581 (ZNF581), mRNA. | | | ZNF581 | | | 1.46 | | | | | | | 0.34 | |
| Homo sapiens KH domain containing, RNA binding, signal transduction associated 3 (KHDRBS3), mRNA. | | | KHDRBS3 | | | 1.46 | | | | | | | 0.53 | |
| Homo sapiens sequestosome 1 (SQSTM1), mRNA. | | | SQSTM1 | | | 1.46 | | | | | | | 0.53 | |
| Homo sapiens phospholipase C, delta 3 (PLCD3), mRNA. | | | PLCD3 | | | 1.46 | | | | | | | 0.53 | |
| Homo sapiens Kruppel-like factor 15 (KLF15), mRNA. | | | KLF15 | | | 1.46 | | | | | | | 0.34 | |
| Homo sapiens basic helix-loop-helix domain containing, class B, 3 (BHLHB3), mRNA. | | | BHLHB3 | | | 1.46 | | | | | | | 0.34 | |
| Homo sapiens 1,4-Alpha-D-Glucan Glucanohydrolase 1 | | | AMY1C | | | 1.45 | | | | | | | 0.70 | |
| Homo sapiens ferritin, heavy polypeptide-like 12 (FTHL12) on chromosome 9. | | | FTHL12 | | | 1.45 | | | | | | | 1.40 | |
| Homo sapiens copine VIII (CPNE8), mRNA. | | | CPNE8 | | | 1.45 | | | | | | | 0.99 | |
| Homo sapiens ankyrin repeat and BTB (POZ) domain containing 1 (ABTB1), transcript variant 1, mRNA. | | | ABTB1 | | | 1.45 | | | | | | | 0.70 | |
| Homo sapiens adenosine A2b receptor (ADORA2B), mRNA. | | | ADORA2B | | | 1.45 | | | | | | | 0.53 | |
| Homo sapiens fatty acyl CoA reductase 2 (FAR2), mRNA. | | | FAR2 | | | 1.45 | | | | | | | 0.53 | |
| Homo sapiens lysosomal-associated membrane protein 2 (LAMP2), transcript variant LAMP2B, mRNA. | | | LAMP2 | | | 1.45 | | | | | | | 0.99 | |
| Homo sapiens kringle containing transmembrane protein 2 (KREMEN2), transcript variant 4, mRNA. | | | KREMEN2 | | | 1.45 | | | | | | | 0.99 | |
| Homo sapiens oxysterol binding protein-like 10 (OSBPL10), mRNA. | | | OSBPL10 | | | 1.44 | | | | | | | 0.53 | |
| Homo sapiens small nucleolar RNA host gene (non-protein coding) 8 (SNHG8) on chromosome 3. | | | SNHG8 | | | 1.44 | | | | | | | 1.05 | |
| Homo sapiens G protein-coupled receptor kinase 5 (GRK5), mRNA. | | | GRK5 | | | 1.44 | | | | | | | 0.34 | |
| Homo sapiens solute carrier family 22 (organic cation transporter), member 18 (SLC22A18), transcript variant 2, mRNA. | | | SLC22A18 | | | 1.44 | | | | | | | 0.34 | |
| Homo sapiens glutathione S-transferase omega 1 (GSTO1), mRNA. | | | GSTO1 | | | 1.44 | | | | | | | 0.53 | |
| Homo sapiens chromosome 12 open reading frame 47 (C12orf47), misc RNA. | | | C12ORF47 | | | 1.44 | | | | | | | 0.70 | |
| Homo sapiens dysbindin (dystrobrevin binding protein 1) domain containing 1 (DBNDD1), transcript variant 1, mRNA. | | | DBNDD1 | | | 1.43 | | | | | | | 0.70 | |
| Homo sapiens 5'-nucleotidase domain containing 3 (NT5DC3), transcript variant 1, mRNA. | | | NT5DC3 | | | 1.43 | | | | | | | 0.53 | |
| Homo sapiens cyclin-dependent kinase inhibitor 2B (p15, inhibits CDK4) (CDKN2B), transcript variant 2, mRNA. | | | CDKN2B | | | 1.43 | | | | | | | 0.34 | |
| Homo sapiens rhomboid 5 homolog 2 (Drosophila) (RHBDF2), transcript variant 2, mRNA. | | | RHBDF2 | | | 1.43 | | | | | | | 0.70 | |
| Homo sapiens sorbin and SH3 domain containing 1 (SORBS1), transcript variant 3, mRNA. | | | SORBS1 | | | 1.43 | | | | | | | 0.70 | |
| Homo sapiens clock homolog (mouse) (CLOCK), mRNA. | | | CLOCK | | | 1.43 | | | | | | | 0.99 | |
| Homo sapiens nebulin (NEB), mRNA. | | | NEB | | | 1.43 | | | | | | | 0.34 | |
| Homo sapiens nicotinamide nucleotide adenylyltransferase 2 (NMNAT2), transcript variant 1, mRNA. | | | NMNAT2 | | | 1.43 | | | | | | | 1.05 | |
| Homo sapiens sprouty homolog 4 (Drosophila) (SPRY4), mRNA. | | | SPRY4 | | | 1.43 | | | | | | | 0.53 | |
| Homo sapiens pregnancy-induced growth inhibitor (OKL38), transcript variant 1, mRNA. | | | OKL38 | | | 1.43 | | | | | | | 0.34 | |
| Homo sapiens K(lysine) acetyltransferase 2B (KAT2B), mRNA. | | | KAT2B | | | 1.42 | | | | | | | 0.70 | |
| Homo sapiens hypothetical protein 284297 (FLJ35258), mRNA. | | | FLJ35258 | | | 1.42 | | | | | | | 0.99 | |
| Homo sapiens ribonuclease T2 (RNASET2), mRNA. | | | RNASET2 | | | 1.42 | | | | | | | 0.70 | |
| Homo sapiens glutamine-fructose-6-phosphate transaminase 2 (GFPT2), mRNA. | | | GFPT2 | | | 1.42 | | | | | | | 0.70 | |
| Homo sapiens ERBB receptor feedback inhibitor 1 (ERRFI1), mRNA. | | | ERRFI1 | | | 1.42 | | | | | | | 0.99 | |
| Homo sapiens RNA, U1G2 small nuclear (RNU1G2), small nuclear RNA. | | | RNU1G2 | | | 1.41 | | | | | | | 0.99 | |
| Homo sapiens contactin associated protein 1 (CNTNAP1), mRNA. | | | CNTNAP1 | | | 1.41 | | | | | | | 0.99 | |
| Homo sapiens parathyroid hormone-like hormone (PTHLH), transcript variant 1, mRNA. | | | PTHLH | | | 1.41 | | | | | | | 1.05 | |
| Homo sapiens transient receptor potential cation channel, subfamily M, member 4 (TRPM4), mRNA. | | | TRPM4 | | | 1.41 | | | | | | | 0.99 | |
| Homo sapiens solute carrier family 1 (glial high affinity glutamate transporter), member 2 (SLC1A2), mRNA. | | | SLC1A2 | | | 1.41 | | | | | | | 1.05 | |
| Homo sapiens solute carrier family 20 (phosphate transporter), member 2 (SLC20A2), mRNA. | | | SLC20A2 | | | 1.41 | | | | | | | 0.99 | |
| Homo sapiens Mitogen-Activated Protein Kinase Kinase 2 | | | MAP2K2 | | | 1.41 | | | | | | | 2.53 | |
| Homo sapiens microsomal glutathione S-transferase 1 (MGST1), transcript variant 1c, mRNA. | | | MGST1 | | | 1.41 | | | | | | | 0.99 | |
| Homo sapiens chitobiase, di-N-acetyl- (CTBS), mRNA. | | | CTBS | | | 1.41 | | | | | | | 0.70 | |
| Homo sapiens inhibitor of DNA binding 3, dominant negative helix-loop-helix protein (ID3), mRNA. | | | ID3 | | | 1.41 | | | | | | | 0.99 | |
| Homo sapiens seizure related 6 homolog (mouse)-like 2 (SEZ6L2), mRNA. | | | SEZ6L2 | | | 1.41 | | | | | | | 0.53 | |
| Homo sapiens K(lysine) acetyltransferase 2A (KAT2A), mRNA. | | | KAT2A | | | 1.41 | | | | | | | 0.70 | |
| Homo sapiens FLJ46906 | | | FLJ46906 | | | 1.41 | | | | | | | 0.99 | |
| Homo sapiens galectin-3 internal gene (GALIG), mRNA. | | | GALIG | | | 1.40 | | | | | | | 0.34 | |
| Homo sapiens Ankyrin Repeat Domain 18A | | | ANKRD18A | | | 1.40 | | | | | | | 4.69 | |
| Homo sapiens integrin, alpha V (vitronectin receptor, alpha polypeptide, antigen CD51) (ITGAV), mRNA. | | | ITGAV | | | 1.40 | | | | | | | 0.70 | |
| Homo sapiens dedicator of cytokinesis 11 (DOCK11), mRNA. | | | DOCK11 | | | 1.40 | | | | | | | 1.05 | |
| Homo sapiens acyl-Coenzyme A dehydrogenase, very long chain (ACADVL), nuclear gene encoding mitochondrial protein, transcript variant 1, mRNA. | | | ACADVL | | | 1.39 | | | | | | | 3.61 | |
| Homo sapiens matrix metallopeptidase 7 (matrilysin, uterine) (MMP7), mRNA. | | | MMP7 | | | 1.39 | | | | | | | 1.05 | |
| Homo sapiens S100 calcium binding protein A16 (S100A16), mRNA. | | | S100A16 | | | 1.39 | | | | | | | 1.40 | |
| Homo sapiens insulin receptor substrate 2 (IRS2), mRNA. | | | IRS2 | | | 1.39 | | | | | | | 1.05 | |
| Homo sapiens GDP-mannose 4,6-dehydratase (GMDS), mRNA. | | | GMDS | | | 1.39 | | | | | | | 0.99 | |
| Homo sapiens lipid phosphate phosphatase-related protein type 5 (LPPR5), transcript variant 1, mRNA. | | | LPPR5 | | | 1.39 | | | | | | | 0.99 | |
| Homo sapiens ephrin-A1 (EFNA1), transcript variant 1, mRNA. | | | EFNA1 | | | 1.39 | | | | | | | 0.70 | |
| Homo sapiens stomatin (STOM), transcript variant 1, mRNA. | | | STOM | | | 1.39 | | | | | | | 1.40 | |
| Homo sapiens PHD finger protein 15 (PHF15), mRNA. | | | PHF15 | | | 1.39 | | | | | | | 0.53 | |
| Homo sapiens Ras and Rab interactor 2 (RIN2), mRNA. | | | RIN2 | | | 1.39 | | | | | | | 1.85 | |
| Homo sapiens regulator of calcineurin 1 (RCAN1), transcript variant 1, mRNA. | | | RCAN1 | | | 1.39 | | | | | | | 1.85 | |
| Homo sapiens synaptojanin 2 (SYNJ2), mRNA. | | | SYNJ2 | | | 1.39 | | | | | | | 0.99 | |
| Homo sapiens calcitonin receptor-like (CALCRL), mRNA. | | | CALCRL | | | 1.39 | | | | | | | 1.05 | |
| Homo sapiens chaperone, ABC1 activity of bc1 complex homolog (S. pombe) (CABC1), nuclear gene encoding mitochondrial protein, mRNA. | | | CABC1 | | | 1.38 | | | | | | | 1.85 | |
| Homo sapiens elongation protein 2 homolog (S. cerevisiae) (ELP2), mRNA. | | | ELP2 | | | 1.38 | | | | | | | 1.05 | |
| Homo sapiens copine IV (CPNE4), mRNA. | | | CPNE4 | | | 1.38 | | | | | | | 1.40 | |
| Homo sapiens chromosome 1 open reading frame 187 (C1orf187), mRNA. | | | C1ORF187 | | | 1.38 | | | | | | | 0.99 | |
| Homo sapiens family with sequence similarity 69, member B (FAM69B), mRNA. | | | FAM69B | | | 1.38 | | | | | | | 0.70 | |
| Homo sapiens poly(rC) binding protein 2 (PCBP2), transcript variant 3, mRNA. | | | PCBP2 | | | 1.38 | | | | | | | 1.85 | |
| Homo sapiens phosphofructokinase, muscle (PFKM), mRNA. | | | PFKM | | | 1.38 | | | | | | | 1.05 | |
| Homo sapiens solute carrier family 25 (mitochondrial thiamine pyrophosphate carrier), member 19 (SLC25A19), nuclear gene encoding mitochondrial protein, mRNA. | | | SLC25A19 | | | 1.38 | | | | | | | 0.99 | |
| Homo sapiens ankyrin repeat domain 36 (ANKRD36), mRNA. | | | ANKRD36 | | | 1.38 | | | | | | | 2.53 | |
| Homo sapiens N-acetylglucosaminidase, alpha- (NAGLU), mRNA. | | | NAGLU | | | 1.38 | | | | | | | 0.70 | |
| Homo sapiens cytochrome c oxidase subunit Vb (COX5B), mRNA. | | | COX5B | | | 1.38 | | | | | | | 1.40 | |
| Homo sapiens phosphodiesterase 4C, cAMP-specific (phosphodiesterase E1 dunce homolog, Drosophila) (PDE4C), mRNA. | | | PDE4C | | | 1.38 | | | | | | | 1.40 | |
| Homo sapiens Family With Sequence Similarity 215 Member A | | | C17ORF88 | | | 1.38 | | | | | | | 3.61 | |
| Homo sapiens BCS1-like (yeast) (BCS1L), nuclear gene encoding mitochondrial protein, transcript variant 2, mRNA. | | | BCS1L | | | 1.38 | | | | | | | 0.99 | |
| Homo sapiens endothelin 1 (EDN1), mRNA. | | | EDN1 | | | 1.38 | | | | | | | 1.05 | |
| Homo sapiens nuclear factor of kappa light polypeptide gene enhancer in B-cells inhibitor, alpha (NFKBIA), mRNA. | | | NFKBIA | | | 1.38 | | | | | | | 1.85 | |
| Homo sapiens ADAM metallopeptidase domain 15 (ADAM15), transcript variant 2, mRNA. | | | ADAM15 | | | 1.37 | | | | | | | 1.40 | |
| Homo sapiens Rho GTPase activating protein 10 (ARHGAP10), mRNA. | | | ARHGAP10 | | | 1.37 | | | | | | | 1.85 | |
| Homo sapiens acetylserotonin O-methyltransferase-like (ASMTL), mRNA. | | | ASMTL | | | 1.37 | | | | | | | 0.99 | |
| Homo sapiens carbohydrate (N-acetylglucosamine 6-O) sulfotransferase 7 (CHST7), mRNA. | | | CHST7 | | | 1.37 | | | | | | | 0.70 | |
| Homo sapiens zinc finger protein 436 (ZNF436), transcript variant 2, mRNA. | | | ZNF436 | | | 1.37 | | | | | | | 0.99 | |
| Homo sapiens chromosome 2 open reading frame 82 (C2orf82), mRNA. | | | C2ORF82 | | | 1.37 | | | | | | | 1.40 | |
| Homo sapiens family with sequence similarity 134, member C (FAM134C), mRNA. | | | FAM134C | | | 1.37 | | | | | | | 1.40 | |
| Homo sapiens major vault protein (MVP), transcript variant 2, mRNA. | | | MVP | | | 1.37 | | | | | | | 1.85 | |
| Homo sapiens similar to 2010300C02Rik protein (MGC42367), mRNA. | | | MGC42367 | | | 1.36 | | | | | | | 1.85 | |
| Homo sapiens chromosome 5 open reading frame 32 (C5orf32), mRNA. | | | C5ORF32 | | | 1.36 | | | | | | | 2.53 | |
| Homo sapiens integrin, alpha 5 (fibronectin receptor, alpha polypeptide) (ITGA5), mRNA. | | | ITGA5 | | | 1.36 | | | | | | | 1.05 | |
| Homo sapiens TAR (HIV-1) RNA binding protein 1 (TARBP1), mRNA. | | | TARBP1 | | | 1.36 | | | | | | | 0.99 | |
| Homo sapiens CD44 molecule (Indian blood group) (CD44), transcript variant 1, mRNA. | | | CD44 | | | 1.36 | | | | | | | 1.40 | |
| Homo sapiens toll-like receptor adaptor molecule 1 (TICAM1), transcript variant 2, mRNA. | | | TICAM1 | | | 1.36 | | | | | | | 1.40 | |
| Homo sapiens Macrophage Stimulating 1 | | | MST1 | | | 1.36 | | | | | | | 1.40 | |
| Homo sapiens AT rich interactive domain 5B (MRF1-like) (ARID5B), mRNA. | | | ARID5B | | | 1.36 | | | | | | | 1.85 | |
| Homo sapiens C2 calcium-dependent domain containing 2 (C2CD2), transcript variant 2, mRNA. | | | C2CD2 | | | 1.36 | | | | | | | 0.99 | |
| Homo sapiens ankyrin repeat domain 24 (ANKRD24), mRNA. | | | ANKRD24 | | | 1.36 | | | | | | | 1.85 | |
| Homo sapiens 1-acylglycerol-3-phosphate O-acyltransferase 2 (lysophosphatidic acid acyltransferase, beta) (AGPAT2), transcript variant 1, mRNA. | | | AGPAT2 | | | 1.36 | | | | | | | 1.40 | |
| Homo sapiens ST6 (alpha-N-acetyl-neuraminyl-2,3-beta-galactosyl-1, 3)-N-acetylgalactosaminide alpha-2,6-sialyltransferase 5 (ST6GALNAC5), mRNA. | | | ST6GALNAC5 | | | 1.36 | | | | | | | 2.53 | |
| Homo sapiens TATA box binding protein (TBP)-associated factor, RNA polymerase I, C, 110kDa (TAF1C), transcript variant 1, mRNA. | | | TAF1C | | | 1.36 | | | | | | | 1.40 | |
| Homo sapiens phosphoinositide-3-kinase interacting protein 1 (PIK3IP1), mRNA. | | | PIK3IP1 | | | 1.36 | | | | | | | 1.40 | |
| Homo sapiens zinc finger protein 365 (ZNF365), transcript variant C, mRNA. | | | ZNF365 | | | 1.36 | | | | | | | 1.05 | |
| Homo sapiens transcription factor CP2-like 1 (TFCP2L1), mRNA. | | | TFCP2L1 | | | 1.36 | | | | | | | 1.40 | |
| Homo sapiens GRB2-related adaptor protein (GRAP), mRNA. | | | GRAP | | | 1.36 | | | | | | | 4.69 | |
| Homo sapiens paired related homeobox 2 (PRRX2), mRNA. | | | PRRX2 | | | 1.36 | | | | | | | 1.85 | |
| Homo sapiens harakiri, BCL2 interacting protein (contains only BH3 domain) (HRK), mRNA. | | | HRK | | | 1.36 | | | | | | | 1.05 | |
| Homo sapiens dendrin (DDN), mRNA. | | | DDN | | | 1.36 | | | | | | | 0.99 | |
| Homo sapiens microRNA 21 (MIR21), microRNA. | | | MIR21 | | | 1.36 | | | | | | | 1.85 | |
| Homo sapiens SHC (Src homology 2 domain containing) transforming protein 1 (SHC1), transcript variant 2, mRNA. | | | SHC1 | | | 1.36 | | | | | | | 0.70 | |
| Homo sapiens peptidyl-tRNA hydrolase 1 homolog (S. cerevisiae) (PTRH1), mRNA. | | | PTRH1 | | | 1.36 | | | | | | | 1.40 | |
| Homo sapiens protein kinase, cAMP-dependent, regulatory, type II, alpha (PRKAR2A), mRNA. | | | PRKAR2A | | | 1.36 | | | | | | | 2.53 | |
| Homo sapiens SPARC-like 1 (mast9, hevin) (SPARCL1), mRNA. | | | SPARCL1 | | | 1.35 | | | | | | | 1.40 | |
| Homo sapiens solute carrier family 38, member 1 (SLC38A1), transcript variant 1, mRNA. | | | SLC38A1 | | | 1.35 | | | | | | | 1.05 | |
| Homo sapiens SCO cytochrome oxidase deficient homolog 2 (yeast) (SCO2), nuclear gene encoding mitochondrial protein, mRNA. | | | SCO2 | | | 1.35 | | | | | | | 1.05 | |
| Homo sapiens radical S-adenosyl methionine domain containing 1 (RSAD1), mRNA. | | | RSAD1 | | | 1.35 | | | | | | | 1.85 | |
| Homo sapiens WEE1 homolog (S. pombe) (WEE1), mRNA. | | | WEE1 | | | 1.35 | | | | | | | 1.40 | |
| Homo sapiens death associated protein-like 1 (DAPL1), mRNA. | | | DAPL1 | | | 1.35 | | | | | | | 1.40 | |
| Homo sapiens cadherin 5, type 2, VE-cadherin (vascular epithelium) (CDH5), mRNA. | | | CDH5 | | | 1.35 | | | | | | | 1.85 | |
| Homo sapiens exosome component 5 (EXOSC5), mRNA. | | | EXOSC5 | | | 1.35 | | | | | | | 1.85 | |
| Homo sapiens carboxypeptidase Z (CPZ), transcript variant 3, mRNA. | | | CPZ | | | 1.35 | | | | | | | 2.53 | |
| Homo sapiens WD Repeat Domain 43 | | | WDR43 | | | 1.35 | | | | | | | 1.85 | |
| Homo sapiens solute carrier family 3 (activators of dibasic and neutral amino acid transport), member 2 (SLC3A2), transcript variant 6, mRNA. | | | SLC3A2 | | | 1.35 | | | | | | | 1.85 | |
| Homo sapiens synemin, intermediate filament protein (SYNM), transcript variant A, mRNA. | | | SYNM | | | 1.35 | | | | | | | 2.53 | |
| Homo sapiens S-adenosylhomocysteine hydrolase-like 2 (AHCYL2), mRNA. | | | AHCYL2 | | | 1.35 | | | | | | | 1.40 | |
| Homo sapiens BRCA2 and CDKN1A interacting protein (BCCIP), transcript variant A, mRNA. | | | BCCIP | | | 1.34 | | | | | | | 3.61 | |
| Homo sapiens fibroblast growth factor receptor-like 1 (FGFRL1), transcript variant 2, mRNA. | | | FGFRL1 | | | 1.34 | | | | | | | 1.40 | |
| Homo sapiens ankyrin repeat, family A (RFXANK-like), 2 (ANKRA2), mRNA. | | | ANKRA2 | | | 1.34 | | | | | | | 1.40 | |
| Homo sapiens ATPase, H+ transporting, lysosomal 31kDa, V1 subunit E1 (ATP6V1E1), transcript variant 3, mRNA. | | | ATP6V1E1 | | | 1.34 | | | | | | | 1.40 | |
| Homo sapiens WD repeat domain 21A (WDR21A), transcript variant 1, mRNA. | | | WDR21A | | | 1.34 | | | | | | | 1.85 | |
| Homo sapiens RNA pseudouridylate synthase domain containing 3 (RPUSD3), mRNA. | | | RPUSD3 | | | 1.34 | | | | | | | 2.53 | |
| Homo sapiens chromosome 21 open reading frame 70 (C21orf70), mRNA. | | | C21ORF70 | | | 1.34 | | | | | | | 3.61 | |
| Homo sapiens fibrinogen C domain containing 1 (FIBCD1), mRNA. | | | FIBCD1 | | | 1.34 | | | | | | | 3.61 | |
| Homo sapiens transketolase (Wernicke-Korsakoff syndrome) (TKT), mRNA. | | | TKT | | | 1.34 | | | | | | | 2.53 | |
| Homo sapiens galactokinase 1 (GALK1), mRNA. | | | GALK1 | | | 1.34 | | | | | | | 1.85 | |
| Homo sapiens chromosome 12 open reading frame 24 (C12orf24), mRNA. | | | C12ORF24 | | | 1.34 | | | | | | | 1.85 | |
| Homo sapiens prolyl 4-hydroxylase, alpha polypeptide II (P4HA2), transcript variant 2, mRNA. | | | P4HA2 | | | 1.34 | | | | | | | 1.85 | |
| Homo sapiens protein tyrosine phosphatase, receptor type, E (PTPRE), transcript variant 1, mRNA. | | | PTPRE | | | 1.34 | | | | | | | 3.61 | |
| Homo sapiens calcium channel, voltage-dependent, beta 2 subunit (CACNB2), transcript variant 4, mRNA. | | | CACNB2 | | | 1.34 | | | | | | | 2.53 | |
| Homo sapiens FERM domain containing 8 (FRMD8), mRNA. | | | FRMD8 | | | 1.34 | | | | | | | 2.53 | |
| Homo sapiens protein tyrosine phosphatase, receptor type, G (PTPRG), mRNA. | | | PTPRG | | | 1.33 | | | | | | | 4.69 | |
| Homo sapiens OAF homolog (Drosophila) (OAF), mRNA. | | | OAF | | | 1.33 | | | | | | | 2.53 | |
| Homo sapiens claudin domain containing 2 (CLDND2), mRNA. | | | CLDND2 | | | 1.33 | | | | | | | 3.61 | |
| Homo sapiens BCL2-like 13 (apoptosis facilitator) (BCL2L13), nuclear gene encoding mitochondrial protein, mRNA. | | | BCL2L13 | | | 1.33 | | | | | | | 1.40 | |
| Homo sapiens thrombospondin 3 (THBS3), mRNA. | | | THBS3 | | | 1.33 | | | | | | | 1.85 | |
| Homo sapiens NBPF Member 8 | | | NBPF8 | | | 1.33 | | | | | | | 3.61 | |
| Homo sapiens SH3 domain binding glutamic acid-rich protein (SH3BGR), transcript variant 2, mRNA. | | | SH3BGR | | | 1.33 | | | | | | | 3.61 | |
| Homo sapiens neurofilament, heavy polypeptide 200kDa (NEFH), mRNA. | | | NEFH | | | 1.33 | | | | | | | 2.53 | |
| Homo sapiens lemur tyrosine kinase 3 (LMTK3), mRNA. | | | LMTK3 | | | 1.33 | | | | | | | 2.53 | |
| Homo sapiens YdjC homolog (bacterial) (YDJC), mRNA. | | | YDJC | | | 1.33 | | | | | | | 1.85 | |
| Homo sapiens ferritin, heavy polypeptide 1 (FTH1), mRNA. | | | FTH1 | | | 1.33 | | | | | | | 1.85 | |
| Homo sapiens echinoderm microtubule associated protein like 2 (EML2), mRNA. | | | EML2 | | | 1.33 | | | | | | | 2.53 | |
| Homo sapiens porcupine homolog (Drosophila) (PORCN), transcript variant C, mRNA. | | | PORCN | | | 1.33 | | | | | | | 1.40 | |
| Homo sapiens LIM domain containing preferred translocation partner in lipoma (LPP), mRNA. | | | LPP | | | 1.33 | | | | | | | 1.40 | |
| Homo sapiens 3-hydroxymethyl-3-methylglutaryl-Coenzyme A lyase (HMGCL), nuclear gene encoding mitochondrial protein, transcript variant 1, mRNA. | | | HMGCL | | | 1.33 | | | | | | | 1.85 | |
| Homo sapiens dipeptidyl-peptidase 7 (DPP7), mRNA. | | | DPP7 | | | 1.33 | | | | | | | 3.61 | |
| Homo sapiens forkhead box O1 (FOXO1), mRNA. | | | FOXO1 | | | 1.33 | | | | | | | 4.69 | |
| Homo sapiens HMG Domain-Containing Protein 3 | | | KIAA0194 | | | 1.33 | | | | | | | 1.85 | |
| Homo sapiens chromosome 14 open reading frame 133 (C14orf133), mRNA. | | | C14ORF133 | | | 1.33 | | | | | | | 1.85 | |
| Homo sapiens microfibrillar-associated protein 4 (MFAP4), mRNA. | | | MFAP4 | | | 1.32 | | | | | | | 1.85 | |
| Homo sapiens related RAS viral (r-ras) oncogene homolog (RRAS), mRNA. | | | RRAS | | | 1.32 | | | | | | | 2.53 | |
| Homo sapiens adenylate kinase 3 (AK3), nuclear gene encoding mitochondrial protein, mRNA. | | | AK3 | | | 1.32 | | | | | | | 1.85 | |
| Homo sapiens phosphoglucomutase 2-like 1 (PGM2L1), mRNA. | | | PGM2L1 | | | 1.32 | | | | | | | 3.61 | |
| Homo sapiens nicotinate phosphoribosyltransferase domain containing 1 (NAPRT1), mRNA. | | | NAPRT1 | | | 1.32 | | | | | | | 4.69 | |
| Homo sapiens oligonucleotide/oligosaccharide-binding fold containing 1 (OBFC1), mRNA. | | | OBFC1 | | | 1.32 | | | | | | | 3.61 | |
| Homo sapiens hairy/enhancer-of-split related with YRPW motif 2 (HEY2), mRNA. | | | HEY2 | | | 1.32 | | | | | | | 3.61 | |
| Homo sapiens bone morphogenetic protein 6 (BMP6), mRNA. | | | BMP6 | | | 1.32 | | | | | | | 3.61 | |
| Homo sapiens stanniocalcin 2 (STC2), mRNA. | | | STC2 | | | 1.32 | | | | | | | 3.61 | |
| Homo sapiens aspartate beta-hydroxylase (ASPH), transcript variant 1, mRNA. | | | ASPH | | | 1.32 | | | | | | | 4.69 | |
| Homo sapiens chromosome 1 open reading frame 152 (C1orf152), non-coding RNA. | | | C1ORF152 | | | 1.32 | | | | | | | 3.61 | |
| Homo sapiens KISS1 receptor (KISS1R), mRNA. | | | KISS1R | | | 1.32 | | | | | | | 3.61 | |
| Homo sapiens chromosome 1 open reading frame 52 (C1orf52), mRNA. | | | C1ORF52 | | | 1.32 | | | | | | | 1.85 | |
| Homo sapiens family with sequence similarity 62 (C2 domain containing) member B (FAM62B), mRNA. | | | FAM62B | | | 1.32 | | | | | | | 1.85 | |
| Homo sapiens estrogen-related receptor alpha pseudogene 2 (ESRRAP2) on chromosome 13. | | | ESRRAP2 | | | 1.32 | | | | | | | 2.53 | |
| Homo sapiens protein O-linked mannose beta1,2-N-acetylglucosaminyltransferase (POMGNT1), mRNA. | | | POMGNT1 | | | 1.32 | | | | | | | 2.53 | |
| Homo sapiens DEP domain containing 6 (DEPDC6), mRNA. | | | DEPDC6 | | | 1.32 | | | | | | | 2.53 | |
| Homo sapiens ring finger protein 149 (RNF149), mRNA. | | | RNF149 | | | 1.32 | | | | | | | 3.61 | |
| Homo sapiens zinc finger protein 277 (ZNF277), mRNA. | | | ZNF277 | | | 1.32 | | | | | | | 1.85 | |
| Homo sapiens zinc finger protein 692 (ZNF692), transcript variant 2, mRNA. | | | ZNF692 | | | 1.32 | | | | | | | 2.53 | |
| Homo sapiens follicular lymphoma variant translocation 1 (FVT1), mRNA. | | | FVT1 | | | 1.32 | | | | | | | 2.53 | |
| Homo sapiens sema domain, transmembrane domain (TM), and cytoplasmic domain, (semaphorin) 6A (SEMA6A), mRNA. | | | SEMA6A | | | 1.32 | | | | | | | 2.53 | |
| Homo sapiens glutaminase (GLS), mRNA. | | | GLS | | | 1.32 | | | | | | | 3.61 | |
| Homo sapiens homer homolog 2 (Drosophila) (HOMER2), transcript variant 4, mRNA. | | | HOMER2 | | | 1.32 | | | | | | | 2.53 | |
| Homo sapiens chromobox homolog 7 (CBX7), mRNA. | | | CBX7 | | | 1.32 | | | | | | | 4.69 | |
| Homo sapiens small nucleolar RNA, C/D box 3A (SNORD3A), small nucleolar RNA. | | | SNORD3A | | | 1.31 | | | | | | | 3.61 | |
| Homo sapiens anoctamin 1, calcium activated chloride channel (ANO1), transcript variant 1, mRNA. | | | ANO1 | | | 1.31 | | | | | | | 3.61 | |
| Homo sapiens mitochondrial tumor suppressor 1 (MTUS1), nuclear gene encoding mitochondrial protein, transcript variant 3, mRNA. | | | MTUS1 | | | 1.31 | | | | | | | 4.69 | |
| Homo sapiens mannosidase, beta A, lysosomal (MANBA), mRNA. | | | MANBA | | | 1.31 | | | | | | | 3.61 | |
| Homo sapiens polymerase (RNA) III (DNA directed) polypeptide G (32kD) (POLR3G), mRNA. | | | POLR3G | | | 1.31 | | | | | | | 2.53 | |
| Homo sapiens malic enzyme 1, NADP(+)-dependent, cytosolic (ME1), mRNA. | | | ME1 | | | 1.31 | | | | | | | 4.69 | |
| Homo sapiens small nucleolar RNA, H/ACA box 8 (SNORA8), small nucleolar RNA. | | | SNORA8 | | | 1.31 | | | | | | | 2.53 | |
| Homo sapiens amyloid beta (A4) precursor protein-binding, family B, member 3 (APBB3), transcript variant 2, mRNA. | | | APBB3 | | | 1.31 | | | | | | | 4.69 | |
| Homo sapiens sprouty-related, EVH1 domain containing 1 (SPRED1), mRNA. | | | SPRED1 | | | 1.31 | | | | | | | 1.40 | |
| Homo sapiens transmembrane protein 150A (TMEM150A), transcript variant 1, mRNA. | | | TMEM150A | | | 1.31 | | | | | | | 2.53 | |
| Homo sapiens N-myc downstream regulated gene 1 (NDRG1), mRNA. | | | NDRG1 | | | 1.31 | | | | | | | 3.61 | |
| Homo sapiens transmembrane protein 42 (TMEM42), mRNA. | | | TMEM42 | | | 1.31 | | | | | | | 4.69 | |
| Homo sapiens solute carrier family 6 (neurotransmitter transporter, GABA), member 1 (SLC6A1), mRNA. | | | SLC6A1 | | | 1.31 | | | | | | | 2.53 | |
| Homo sapiens biliverdin reductase B (flavin reductase (NADPH)) (BLVRB), mRNA. | | | BLVRB | | | 1.31 | | | | | | | 2.53 | |
| Homo sapiens PRA1 domain family, member 2 (PRAF2), mRNA. | | | PRAF2 | | | 1.31 | | | | | | | 4.69 | |
| Homo sapiens zinc fingers and homeoboxes 3 (ZHX3), mRNA. | | | ZHX3 | | | 1.31 | | | | | | | 3.61 | |
| Homo sapiens corticotropin releasing hormone (CRH), mRNA. | | | CRH | | | 1.31 | | | | | | | 4.69 | |
| Homo sapiens prion protein (PRNP), transcript variant 2, mRNA. | | | PRNP | | | 1.31 | | | | | | | 2.53 | |
| Homo sapiens plasminogen activator, tissue (PLAT), transcript variant 1, mRNA. | | | PLAT | | | 1.31 | | | | | | | 3.61 | |
| Homo sapiens spire homolog 1 (Drosophila) (SPIRE1), transcript variant 2, mRNA. | | | SPIRE1 | | | 1.31 | | | | | | | 3.61 | |
| Homo sapiens solute carrier family 39 (zinc transporter), member 14 (SLC39A14), mRNA. | | | SLC39A14 | | | 1.30 | | | | | | | 2.53 | |
| Homo sapiens prostate transmembrane protein, androgen induced 1 (PMEPA1), transcript variant 2, mRNA. | | | PMEPA1 | | | 1.30 | | | | | | | 4.69 | |
| Homo sapiens tetratricopeptide repeat domain 17 (TTC17), mRNA. | | | TTC17 | | | 1.30 | | | | | | | 1.85 | |
| Homo sapiens carbonic anhydrase XII (CA12), transcript variant 1, mRNA. | | | CA12 | | | 1.30 | | | | | | | 4.69 | |
| Homo sapiens early growth response 2 (Krox-20 homolog, Drosophila) (EGR2), mRNA. | | | EGR2 | | | 1.30 | | | | | | | 3.61 | |
| Homo sapiens transmembrane protein 59-like (TMEM59L), mRNA. | | | TMEM59L | | | 1.30 | | | | | | | 4.69 | |
| Homo sapiens amylase, alpha 1A (salivary) (AMY1A), transcript variant 1, mRNA. | | | AMY1A | | | 1.30 | | | | | | | 4.69 | |
| Homo sapiens leucine-rich PPR-motif containing (LRPPRC), mRNA. | | | LRPPRC | | | 1.30 | | | | | | | 4.69 | |
| Homo sapiens coiled-coil-helix-coiled-coil-helix domain containing 10 (CHCHD10), mRNA. | | | CHCHD10 | | | 1.30 | | | | | | | 3.61 | |
| Homo sapiens Poly(A) Binding Protein Cytoplasmic 1 Like | | | PABPC1L | | | 1.30 | | | | | | | 2.53 | |
| Homo sapiens spermine oxidase (SMOX), transcript variant 4, mRNA. | | | SMOX | | | 1.30 | | | | | | | 4.69 | |
| Homo sapiens Inositol 1,4,5-Trisphosphate Receptor-Interacting Protein-Like 2 | | | ITPRIPL2 | | | 1.30 | | | | | | | 4.69 | |
| Homo sapiens chromosome 5 open reading frame 4 (C5orf4), mRNA. | | | C5ORF4 | | | 1.30 | | | | | | | 4.69 | |
| Homo sapiens syntaxin 3 (STX3), mRNA. | | | STX3 | | | 1.30 | | | | | | | 4.69 | |
| Homo sapiens family with sequence similarity 134, member A (FAM134A), mRNA. | | | FAM134A | | | 1.30 | | | | | | | 4.69 | |
| Homo sapiens Fc fragment of IgG binding protein (FCGBP), mRNA. | | | FCGBP | | | 1.30 | | | | | | | 3.61 | |
| Homo sapiens forkhead box C1 (FOXC1), mRNA. | | | FOXC1 | | | 1.30 | | | | | | | 3.61 | |
| Homo sapiens acyl-Coenzyme A dehydrogenase family, member 11 (ACAD11), mRNA. | | | ACAD11 | | | 1.30 | | | | | | | 2.53 | |
| Homo sapiens interleukin-1 receptor-associated kinase 2 (IRAK2), mRNA. | | | IRAK2 | | | 1.30 | | | | | | | 4.69 | |
| Homo sapiens patatin-like phospholipase domain containing 7 (PNPLA7), mRNA. | | | PNPLA7 | | | 1.30 | | | | | | | 2.53 | |
| Homo sapiens glutathione S-transferase omega 2 (GSTO2), mRNA. | | | GSTO2 | | | 1.30 | | | | | | | 2.53 | |
| Homo sapiens neuro-oncological ventral antigen 1 (NOVA1), transcript variant 3, mRNA. | | | NOVA1 | | | 1.30 | | | | | | | 2.53 | |
| Homo sapiens chromosome 5 open reading frame 28 (C5orf28), mRNA. | | | C5ORF28 | | | 1.29 | | | | | | | 4.69 | |
| Homo sapiens solute carrier family 25 (mitochondrial carrier: glutamate), member 22 (SLC25A22), nuclear gene encoding mitochondrial protein, mRNA. | | | SLC25A22 | | | 1.29 | | | | | | | 4.69 | |
| Homo sapiens ATP-binding cassette, sub-family B (MDR/TAP), member 1 (ABCB1), mRNA. | | | ABCB1 | | | 1.29 | | | | | | | 4.69 | |
| Homo sapiens zinc finger, AN1-type domain 1 (ZFAND1), mRNA. | | | ZFAND1 | | | 1.29 | | | | | | | 3.61 | |
| Homo sapiens S100 calcium binding protein A10 (S100A10), mRNA. | | | S100A10 | | | 1.29 | | | | | | | 3.61 | |
| Homo sapiens branched chain keto acid dehydrogenase E1, alpha polypeptide (BCKDHA), mRNA. | | | BCKDHA | | | 1.29 | | | | | | | 4.69 | |
| Homo sapiens tropomyosin 2 (beta) (TPM2), transcript variant 1, mRNA. | | | TPM2 | | | 1.29 | | | | | | | 4.69 | |
| Homo sapiens Shwachman-Bodian-Diamond syndrome (SBDS), mRNA. | | | SBDS | | | 1.29 | | | | | | | 4.69 | |
| Homo sapiens DENN/MADD domain containing 4C (DENND4C), mRNA. | | | DENND4C | | | 1.29 | | | | | | | 4.69 | |
| Homo sapiens pecanex-like 2 (Drosophila) (PCNXL2), transcript variant 1, mRNA. | | | PCNXL2 | | | 1.29 | | | | | | | 4.69 | |
| Homo sapiens F-box protein 6 (FBXO6), mRNA. | | | FBXO6 | | | 1.29 | | | | | | | 2.53 | |
| Homo sapiens ATG7 autophagy related 7 homolog (S. cerevisiae) (ATG7), mRNA. | | | ATG7 | | | 1.29 | | | | | | | 4.69 | |
| Homo sapiens echinoderm microtubule associated protein like 3 (EML3), mRNA. | | | EML3 | | | 1.29 | | | | | | | 3.61 | |
| Homo sapiens cold shock domain containing C2, RNA binding (CSDC2), mRNA. | | | CSDC2 | | | 1.29 | | | | | | | 4.69 | |
| Homo sapiens adenylate cyclase 9 (ADCY9), mRNA. | | | ADCY9 | | | 1.28 | | | | | | | 4.69 | |
| Homo sapiens protein arginine methyltransferase 3 (PRMT3), mRNA. | | | PRMT3 | | | 1.28 | | | | | | | 3.61 | |
| Homo sapiens opiate receptor-like 1 (OPRL1), transcript variant 1, mRNA. | | | OPRL1 | | | 1.28 | | | | | | | 4.69 | |
| Homo sapiens succinate-CoA ligase, GDP-forming, beta subunit (SUCLG2), mRNA. | | | SUCLG2 | | | 1.28 | | | | | | | 4.69 | |
| Homo sapiens solute carrier family 41, member 3 (SLC41A3), transcript variant 4, mRNA. | | | SLC41A3 | | | 1.28 | | | | | | | 4.69 | |
| Homo sapiens solute carrier family 35, member F2 (SLC35F2), mRNA. | | | SLC35F2 | | | 1.28 | | | | | | | 3.61 | |
| Homo sapiens citrate lyase beta like (CLYBL), mRNA. | | | CLYBL | | | 1.28 | | | | | | | 4.69 | |
| Homo sapiens NOP2/Sun domain family, member 5C (NSUN5C), transcript variant 1, mRNA. | | | NSUN5C | | | 1.28 | | | | | | | 4.69 | |
| Homo sapiens golgi autoantigen, golgin subfamily a, 8B (GOLGA8B), mRNA. | | | GOLGA8B | | | 1.28 | | | | | | | 4.69 | |
| Homo sapiens transcobalamin II; macrocytic anemia (TCN2), mRNA. | | | TCN2 | | | 1.28 | | | | | | | 4.69 | |
| Homo sapiens chromosome 4 open reading frame 14 (C4orf14), mRNA. | | | C4ORF14 | | | 1.28 | | | | | | | 4.69 | |
| Homo sapiens scleraxis homolog A (mouse) (SCXA), mRNA. | | | SCXA | | | 1.28 | | | | | | | 4.69 | |
| Homo sapiens NIPA-like domain containing 3 (NPAL3), mRNA. | | | NPAL3 | | | 1.27 | | | | | | | 4.69 | |
| Homo sapiens pseudouridylate synthase 1 (PUS1), transcript variant 1, mRNA. | | | PUS1 | | | 1.27 | | | | | | | 4.69 | |
| Homo sapiens zinc finger, MYND-type containing 8 (ZMYND8), transcript variant 3, mRNA. | | | ZMYND8 | | | 1.27 | | | | | | | 4.69 | |
| Homo sapiens neuroblastoma breakpoint family, member 14 (NBPF14), mRNA. | | | NBPF14 | | | 1.27 | | | | | | | 4.69 | |
| Homo sapiens phosphoinositide-3-kinase, regulatory subunit 3 (p55, gamma) (PIK3R3), mRNA. | | | PIK3R3 | | | 1.17 | | | | | | | 1.75 | |
| Homo sapiens RAS, dexamethasone-induced 1 (RASD1), mRNA. | | | RASD1 | | | -1.12 | | | | | | | 4.69 | |
| Homo sapiens cathepsin C (CTSC), transcript variant 2, mRNA. | | | CTSC | | | -1.20 | | | | | | | 4.69 | |
| Homo sapiens dolichyl pyrophosphate phosphatase 1 (DOLPP1), mRNA. | | | DOLPP1 | | | -1.20 | | | | | | | 4.69 | |
| Homo sapiens calcyclin binding protein (CACYBP), transcript variant 1, mRNA. | | | CACYBP | | | -1.21 | | | | | | | 4.69 | |
| Homo sapiens mex-3 homolog D (C. elegans) (MEX3D), transcript variant 2, mRNA. | | | MEX3D | | | -1.21 | | | | | | | 4.69 | |
| Homo sapiens polymerase (RNA) III (DNA directed) polypeptide B (POLR3B), mRNA. | | | POLR3B | | | -1.21 | | | | | | | 3.61 | |
| Homo sapiens sal-like 3 (Drosophila) (SALL3), mRNA. | | | SALL3 | | | -1.21 | | | | | | | 4.69 | |
| Homo sapiens RAN binding protein 1 (RANBP1), mRNA. | | | RANBP1 | | | -1.21 | | | | | | | 3.61 | |
| Homo sapiens protein phosphatase 1D magnesium-dependent, delta isoform (PPM1D), mRNA. | | | PPM1D | | | -1.21 | | | | | | | 4.69 | |
| Homo sapiens helicase, lymphoid-specific (HELLS), mRNA. | | | HELLS | | | -1.21 | | | | | | | 4.69 | |
| Homo sapiens protein (peptidylprolyl cis/trans isomerase) NIMA-interacting, 4 (parvulin) (PIN4), mRNA. | | | PIN4 | | | -1.21 | | | | | | | 4.69 | |
| Homo sapiens leucine rich repeat containing 20 (LRRC20), transcript variant 2, mRNA. | | | LRRC20 | | | -1.21 | | | | | | | 4.69 | |
| Homo sapiens ADP-ribosylation factor-like 3 (ARL3), mRNA. | | | ARL3 | | | -1.22 | | | | | | | 4.69 | |
| Homo sapiens mitochondrial ribosomal protein L52 (MRPL52), nuclear gene encoding mitochondrial protein, transcript variant 2, mRNA. | | | MRPL52 | | | -1.22 | | | | | | | 4.69 | |
| Homo sapiens profilin 2 (PFN2), transcript variant 1, mRNA. | | | PFN2 | | | -1.22 | | | | | | | 4.69 | |
| Homo sapiens dapper, antagonist of beta-catenin, homolog 2 (Xenopus laevis) (DACT2), mRNA. | | | DACT2 | | | -1.22 | | | | | | | 4.69 | |
| Homo sapiens nucleoporin 62kDa (NUP62), transcript variant 1, mRNA. | | | NUP62 | | | -1.22 | | | | | | | 2.53 | |
| Homo sapiens splicing factor, arginine/serine-rich 2 (SFRS2), mRNA. | | | SFRS2 | | | -1.22 | | | | | | | 4.69 | |
| Homo sapiens phosphoprotein associated with glycosphingolipid microdomains 1 (PAG1), mRNA. | | | PAG1 | | | -1.22 | | | | | | | 4.69 | |
| Homo sapiens mex-3 homolog B (C. elegans) (MEX3B), mRNA. | | | MEX3B | | | -1.22 | | | | | | | 4.69 | |
| Homo sapiens chromosome 16 open reading frame 33 (C16orf33), mRNA. | | | C16ORF33 | | | -1.22 | | | | | | | 3.61 | |
| Homo sapiens transcription elongation factor B (SIII), polypeptide 2 (18kDa, elongin B) (TCEB2), transcript variant 1, mRNA. | | | TCEB2 | | | -1.22 | | | | | | | 4.69 | |
| Homo sapiens polycomb group ring finger 3 (PCGF3), mRNA. | | | PCGF3 | | | -1.22 | | | | | | | 2.53 | |
| Homo sapiens sema domain, immunoglobulin domain (Ig), transmembrane domain (TM) and short cytoplasmic domain, (semaphorin) 4D (SEMA4D), mRNA. | | | SEMA4D | | | -1.22 | | | | | | | 4.69 | |
| Homo sapiens heterogeneous nuclear ribonucleoprotein D (AU-rich element RNA binding protein 1, 37kDa) (HNRNPD), transcript variant 2, mRNA. | | | HNRNPD | | | -1.22 | | | | | | | 3.61 | |
| Homo sapiens CD27-binding (Siva) protein (SIVA), transcript variant 2, mRNA. | | | SIVA | | | -1.22 | | | | | | | 4.69 | |
| Homo sapiens cytochrome c, somatic (CYCS), nuclear gene encoding mitochondrial protein, mRNA. | | | CYCS | | | -1.22 | | | | | | | 3.61 | |
| Homo sapiens antigen identified by monoclonal antibody Ki-67 (MKI67), mRNA. | | | MKI67 | | | -1.22 | | | | | | | 3.61 | |
| Homo sapiens Meis homeobox 3 pseudogene 1 (MEIS3P1), non-coding RNA. | | | MEIS3P1 | | | -1.22 | | | | | | | 3.61 | |
| Homo sapiens minichromosome maintenance complex component 6 (MCM6), mRNA. | | | MCM6 | | | -1.22 | | | | | | | 4.69 | |
| Homo sapiens erythrocyte membrane protein band 4.1 like 5 (EPB41L5), mRNA. | | | EPB41L5 | | | -1.22 | | | | | | | 2.53 | |
| Homo sapiens DnaJ (Hsp40) homolog, subfamily B, member 1 (DNAJB1), mRNA. | | | DNAJB1 | | | -1.22 | | | | | | | 4.69 | |
| Homo sapiens meiotic nuclear divisions 1 homolog (S. cerevisiae) (MND1), mRNA. | | | MND1 | | | -1.22 | | | | | | | 4.69 | |
| Homo sapiens replication factor C (activator 1) 3, 38kDa (RFC3), transcript variant 2, mRNA. | | | RFC3 | | | -1.22 | | | | | | | 4.69 | |
| Homo sapiens nuclear factor I/B (NFIB), mRNA. | | | NFIB | | | -1.22 | | | | | | | 4.69 | |
| Homo sapiens sideroflexin 5 (SFXN5), mRNA. | | | SFXN5 | | | -1.22 | | | | | | | 2.53 | |
| Homo sapiens golgi reassembly stacking protein 2, 55kDa (GORASP2), mRNA. | | | GORASP2 | | | -1.22 | | | | | | | 3.61 | |
| Homo sapiens nucleoporin 107kDa (NUP107), mRNA. | | | NUP107 | | | -1.22 | | | | | | | 3.61 | |
| Homo sapiens mitogen-activated protein kinase kinase kinase 14 (MAP3K14), mRNA. | | | MAP3K14 | | | -1.22 | | | | | | | 3.61 | |
| Homo sapiens phosducin (PDC), transcript variant 1, mRNA. | | | PDC | | | -1.22 | | | | | | | 4.69 | |
| Homo sapiens chromosome 14 open reading frame 106 (C14orf106), mRNA. | | | C14ORF106 | | | -1.23 | | | | | | | 4.69 | |
| Homo sapiens Glucoside Xylosyltransferase 1 | | | GXYLT1 | | | -1.23 | | | | | | | 4.69 | |
| Homo sapiens retinoblastoma binding protein 9 (RBBP9), mRNA. | | | RBBP9 | | | -1.23 | | | | | | | 3.61 | |
| Homo sapiens myristoylated alanine-rich protein kinase C substrate (MARCKS), mRNA. | | | MARCKS | | | -1.23 | | | | | | | 3.61 | |
| Homo sapiens zinc finger protein 22 (KOX 15) (ZNF22), mRNA. | | | ZNF22 | | | -1.23 | | | | | | | 2.53 | |
| Homo sapiens CCCTC-binding factor (zinc finger protein) (CTCF), mRNA. | | | CTCF | | | -1.23 | | | | | | | 4.69 | |
| Homo sapiens putative homeodomain transcription factor 1 (PHTF1), mRNA. | | | PHTF1 | | | -1.23 | | | | | | | 4.69 | |
| Homo sapiens DENN/MADD domain containing 1A (DENND1A), transcript variant 2, mRNA. | | | DENND1A | | | -1.23 | | | | | | | 2.53 | |
| Homo sapiens phosphoglycerate mutase family member 4 (PGAM4), mRNA. | | | PGAM4 | | | -1.23 | | | | | | | 3.61 | |
| Homo sapiens Sp4 transcription factor (SP4), mRNA. | | | SP4 | | | -1.23 | | | | | | | 4.69 | |
| Homo sapiens Holliday junction recognition protein (HJURP), mRNA. | | | HJURP | | | -1.23 | | | | | | | 2.53 | |
| Homo sapiens bromodomain containing protein 75 kDa pseudogene (BP75) on chromosome 6. | | | BP75 | | | -1.23 | | | | | | | 3.61 | |
| Homo sapiens sorting nexin 26 (SNX26), mRNA. | | | SNX26 | | | -1.23 | | | | | | | 3.61 | |
| Homo sapiens thymocyte nuclear protein 1 (THYN1), transcript variant 2, mRNA. | | | THYN1 | | | -1.23 | | | | | | | 3.61 | |
| Homo sapiens enhancer of yellow 2 homolog (Drosophila) (ENY2), mRNA. | | | ENY2 | | | -1.23 | | | | | | | 3.61 | |
| Homo sapiens tripartite motif-containing 36 (TRIM36), transcript variant 1, mRNA. | | | TRIM36 | | | -1.23 | | | | | | | 1.85 | |
| Homo sapiens Transmembrane Protein 249 | | | C8ORFK29 | | | -1.23 | | | | | | | 3.61 | |
| Homo sapiens acyl-Coenzyme A dehydrogenase, C-4 to C-12 straight chain (ACADM), nuclear gene encoding mitochondrial protein, mRNA. | | | ACADM | | | -1.23 | | | | | | | 3.61 | |
| Homo sapiens metastasis suppressor 1 (MTSS1), mRNA. | | | MTSS1 | | | -1.23 | | | | | | | 4.69 | |
| Homo sapiens guanine nucleotide binding protein (G protein), gamma 4 (GNG4), transcript variant 2, mRNA. | | | GNG4 | | | -1.23 | | | | | | | 3.61 | |
| Homo sapiens transcription factor 4 (TCF4), transcript variant 2, mRNA. | | | TCF4 | | | -1.23 | | | | | | | 3.61 | |
| Homo sapiens nuclear transport factor 2 (NUTF2), mRNA. | | | NUTF2 | | | -1.23 | | | | | | | 4.69 | |
| Homo sapiens retinoic acid receptor responder (tazarotene induced) 3 (RARRES3), mRNA. | | | RARRES3 | | | -1.23 | | | | | | | 3.61 | |
| Homo sapiens TRM5 tRNA methyltransferase 5 homolog (S. cerevisiae) (TRMT5), mRNA. | | | TRMT5 | | | -1.23 | | | | | | | 3.61 | |
| Homo sapiens phosphoserine phosphatase (PSPH), mRNA. | | | PSPH | | | -1.23 | | | | | | | 4.69 | |
| Homo sapiens SIVA1, apoptosis-inducing factor (SIVA1), transcript variant 1, mRNA. | | | SIVA1 | | | -1.23 | | | | | | | 2.53 | |
| Homo sapiens interleukin enhancer binding factor 3, 90kDa (ILF3), transcript variant 1, mRNA. | | | ILF3 | | | -1.23 | | | | | | | 3.61 | |
| Homo sapiens retinoblastoma binding protein 4 (RBBP4), transcript variant 2, mRNA. | | | RBBP4 | | | -1.23 | | | | | | | 2.53 | |
| Homo sapiens geminin, DNA replication inhibitor (GMNN), mRNA. | | | GMNN | | | -1.23 | | | | | | | 3.61 | |
| Homo sapiens drebrin 1 (DBN1), transcript variant 1, mRNA. | | | DBN1 | | | -1.24 | | | | | | | 2.53 | |
| Homo sapiens denticleless homolog (Drosophila) (DTL), mRNA. | | | DTL | | | -1.24 | | | | | | | 3.61 | |
| Homo sapiens NCK adaptor protein 2 (NCK2), transcript variant 2, mRNA. | | | NCK2 | | | -1.24 | | | | | | | 2.53 | |
| Homo sapiens TPX2, microtubule-associated, homolog (Xenopus laevis) (TPX2), mRNA. | | | TPX2 | | | -1.24 | | | | | | | 3.61 | |
| Homo sapiens Bloom syndrome, RecQ helicase-like (BLM), mRNA. | | | BLM | | | -1.24 | | | | | | | 3.61 | |
| Homo sapiens zinc finger protein 184 (ZNF184), mRNA. | | | ZNF184 | | | -1.24 | | | | | | | 3.61 | |
| Homo sapiens cathepsin L2 (CTSL2), mRNA. | | | CTSL2 | | | -1.24 | | | | | | | 2.53 | |
| Homo sapiens resistance to inhibitors of cholinesterase 8 homolog B (C. elegans) (RIC8B), mRNA. | | | RIC8B | | | -1.24 | | | | | | | 3.61 | |
| Homo sapiens LIM and calponin homology domains 1 (LIMCH1), mRNA. | | | LIMCH1 | | | -1.24 | | | | | | | 1.85 | |
| Homo sapiens ribonucleotide reductase M2 polypeptide (RRM2), mRNA. | | | RRM2 | | | -1.24 | | | | | | | 3.61 | |
| Homo sapiens paraneoplastic antigen MA1 (PNMA1), mRNA. | | | PNMA1 | | | -1.24 | | | | | | | 4.69 | |
| Homo sapiens kelch-like 7 (Drosophila) (KLHL7), transcript variant 2, mRNA. | | | KLHL7 | | | -1.24 | | | | | | | 3.61 | |
| Homo sapiens Sin3A-associated protein, 30kDa (SAP30), mRNA. | | | SAP30 | | | -1.24 | | | | | | | 3.61 | |
| Homo sapiens inhibitor of growth family, member 3 (ING3), transcript variant 1, mRNA. | | | ING3 | | | -1.24 | | | | | | | 4.69 | |
| Homo sapiens plexin B1 (PLXNB1), mRNA. | | | PLXNB1 | | | -1.24 | | | | | | | 2.53 | |
| Homo sapiens death-domain associated protein (DAXX), mRNA. | | | DAXX | | | -1.24 | | | | | | | 1.40 | |
| Homo sapiens replication factor C (activator 1) 2, 40kDa (RFC2), transcript variant 1, mRNA. | | | RFC2 | | | -1.24 | | | | | | | 3.61 | |
| Homo sapiens dynein, light chain, LC8-type 1 (DYNLL1), transcript variant 1, mRNA. | | | DYNLL1 | | | -1.24 | | | | | | | 3.61 | |
| Homo sapiens heat shock 105kDa/110kDa protein 1 (HSPH1), mRNA. | | | HSPH1 | | | -1.24 | | | | | | | 3.61 | |
| Homo sapiens heterogeneous nuclear ribonucleoprotein H1 (H) (HNRNPH1), mRNA. | | | HNRNPH1 | | | -1.24 | | | | | | | 2.53 | |
| Homo sapiens kinesin family member 14 (KIF14), mRNA. | | | KIF14 | | | -1.24 | | | | | | | 1.40 | |
| Homo sapiens minichromosome maintenance complex component 3 (MCM3), mRNA. | | | MCM3 | | | -1.24 | | | | | | | 2.53 | |
| Homo sapiens MU-2/AP1M2 domain containing, death-inducing (MUDENG), mRNA. | | | MUDENG | | | -1.24 | | | | | | | 3.61 | |
| Homo sapiens HAUS augmin-like complex, subunit 8 (HAUS8), transcript variant 2, mRNA. | | | HAUS8 | | | -1.24 | | | | | | | 3.61 | |
| Homo sapiens fucosyltransferase 9 (alpha (1,3) fucosyltransferase) (FUT9), mRNA. | | | FUT9 | | | -1.24 | | | | | | | 3.61 | |
| Homo sapiens shroom family member 2 (SHROOM2), mRNA. | | | SHROOM2 | | | -1.24 | | | | | | | 4.69 | |
| Homo sapiens metallophosphoesterase domain containing 2 (MPPED2), mRNA. | | | MPPED2 | | | -1.24 | | | | | | | 3.61 | |
| Homo sapiens replication factor C (activator 1) 5, 36.5kDa (RFC5), transcript variant 1, mRNA. | | | RFC5 | | | -1.24 | | | | | | | 3.61 | |
| Homo sapiens dedicator of cytokinesis 10 (DOCK10), mRNA. | | | DOCK10 | | | -1.24 | | | | | | | 2.53 | |
| Homo sapiens solute carrier family 25, member 13 (citrin) (SLC25A13), mRNA. | | | SLC25A13 | | | -1.24 | | | | | | | 4.69 | |
| Homo sapiens scaffold attachment factor B2 (SAFB2), mRNA. | | | SAFB2 | | | -1.24 | | | | | | | 1.85 | |
| Homo sapiens actin-like 6A (ACTL6A), transcript variant 2, mRNA. | | | ACTL6A | | | -1.24 | | | | | | | 2.53 | |
| Homo sapiens proprotein convertase subtilisin/kexin type 5 (PCSK5), mRNA. | | | PCSK5 | | | -1.24 | | | | | | | 3.61 | |
| Homo sapiens cyclin B1 (CCNB1), mRNA. | | | CCNB1 | | | -1.24 | | | | | | | 2.53 | |
| Homo sapiens Fanconi anemia, complementation group C (FANCC), mRNA. | | | FANCC | | | -1.24 | | | | | | | 1.85 | |
| Homo sapiens activating transcription factor 3 (ATF3), transcript variant 4, mRNA. | | | ATF3 | | | -1.24 | | | | | | | 2.53 | |
| Homo sapiens polymerase (DNA directed), alpha 1, catalytic subunit (POLA1), mRNA. | | | POLA1 | | | -1.24 | | | | | | | 3.61 | |
| Homo sapiens transmembrane protein 81 (TMEM81), mRNA. | | | TMEM81 | | | -1.24 | | | | | | | 4.69 | |
| Homo sapiens transmembrane protein 51 (TMEM51), mRNA. | | | TMEM51 | | | -1.24 | | | | | | | 4.69 | |
| Homo sapiens immediate early response 5 (IER5), mRNA. | | | IER5 | | | -1.24 | | | | | | | 2.53 | |
| Homo sapiens branched chain aminotransferase 1, cytosolic (BCAT1), mRNA. | | | BCAT1 | | | -1.24 | | | | | | | 3.61 | |
| Homo sapiens heterogeneous nuclear ribonucleoprotein R (HNRNPR), transcript variant 1, mRNA. | | | HNRNPR | | | -1.24 | | | | | | | 2.53 | |
| Homo sapiens upstream binding transcription factor, RNA polymerase I (UBTF), transcript variant 1, mRNA. | | | UBTF | | | -1.24 | | | | | | | 2.53 | |
| Homo sapiens transcription factor Dp-1 (TFDP1), mRNA. | | | TFDP1 | | | -1.24 | | | | | | | 2.53 | |
| Homo sapiens platelet-activating factor acetylhydrolase, isoform Ib, gamma subunit 29kDa (PAFAH1B3), mRNA. | | | PAFAH1B3 | | | -1.24 | | | | | | | 3.61 | |
| Homo sapiens FK506 binding protein 3, 25kDa (FKBP3), mRNA. | | | FKBP3 | | | -1.24 | | | | | | | 1.85 | |
| Homo sapiens cell division cycle 2, G1 to S and G2 to M (CDC2), transcript variant 1, mRNA. | | | CDC2 | | | -1.25 | | | | | | | 2.53 | |
| Homo sapiens pleckstrin homology domain interacting protein (PHIP), mRNA. | | | PHIP | | | -1.25 | | | | | | | 1.85 | |
| Homo sapiens microtubule-associated protein 6 (MAP6), transcript variant 2, mRNA. | | | MAP6 | | | -1.25 | | | | | | | 2.53 | |
| Homo sapiens chromosome 14 open reading frame 80 (C14orf80), mRNA. | | | C14ORF80 | | | -1.25 | | | | | | | 3.61 | |
| Homo sapiens Fanconi anemia, complementation group I (FANCI), transcript variant 2, mRNA. | | | FANCI | | | -1.25 | | | | | | | 2.53 | |
| Homo sapiens H2A histone family, member Y2 (H2AFY2), mRNA. | | | H2AFY2 | | | -1.25 | | | | | | | 4.69 | |
| Homo sapiens microsomal triglyceride transfer protein (MTTP), mRNA. | | | MTTP | | | -1.25 | | | | | | | 1.85 | |
| Homo sapiens leucine rich repeat and coiled-coil domain containing 1 (LRRCC1), transcript variant 1, mRNA. | | | LRRCC1 | | | -1.25 | | | | | | | 3.61 | |
| Homo sapiens phosphoglycerate dehydrogenase (PHGDH), mRNA. | | | PHGDH | | | -1.25 | | | | | | | 1.85 | |
| Homo sapiens von Hippel-Lindau binding protein 1 (VBP1), mRNA. | | | VBP1 | | | -1.25 | | | | | | | 2.53 | |
| Homo sapiens retinoic acid induced 14 (RAI14), mRNA. | | | RAI14 | | | -1.25 | | | | | | | 2.53 | |
| Homo sapiens transcription factor 3 (E2A immunoglobulin enhancer binding factors E12/E47) (TCF3), mRNA. | | | TCF3 | | | -1.25 | | | | | | | 2.53 | |
| Homo sapiens KIAA1967 (KIAA1967), transcript variant 2, mRNA. | | | KIAA1967 | | | -1.25 | | | | | | | 2.53 | |
| Homo sapiens protein arginine methyltransferase 2 (PRMT2), transcript variant 2, mRNA. | | | PRMT2 | | | -1.25 | | | | | | | 2.53 | |
| Homo sapiens isocitrate dehydrogenase 1 (NADP+), soluble (IDH1), mRNA. | | | IDH1 | | | -1.25 | | | | | | | 2.53 | |
| Homo sapiens Zwilch, kinetochore associated, homolog (Drosophila) (ZWILCH), transcript variant 2, transcribed RNA. | | | ZWILCH | | | -1.25 | | | | | | | 1.85 | |
| Homo sapiens nucleoporin 188kDa (NUP188), mRNA. | | | NUP188 | | | -1.25 | | | | | | | 2.53 | |
| Homo sapiens DCP2 decapping enzyme homolog (S. cerevisiae) (DCP2), mRNA. | | | DCP2 | | | -1.25 | | | | | | | 2.53 | |
| Homo sapiens polo-like kinase 4 (Drosophila) (PLK4), mRNA. | | | PLK4 | | | -1.25 | | | | | | | 1.85 | |
| Homo sapiens guanine nucleotide binding protein (G protein), alpha transducing activity polypeptide 1 (GNAT1), transcript variant 2, mRNA. | | | GNAT1 | | | -1.25 | | | | | | | 3.61 | |
| Homo sapiens transmembrane protein 149 (TMEM149), mRNA. | | | TMEM149 | | | -1.25 | | | | | | | 1.85 | |
| Homo sapiens WW and C2 domain containing 1 (WWC1), mRNA. | | | WWC1 | | | -1.25 | | | | | | | 1.40 | |
| Homo sapiens lethal giant larvae homolog 1 (Drosophila) (LLGL1), mRNA. | | | LLGL1 | | | -1.25 | | | | | | | 1.85 | |
| Homo sapiens phosphoribosyl transferase domain containing 1 (PRTFDC1), mRNA. | | | PRTFDC1 | | | -1.25 | | | | | | | 3.61 | |
| Homo sapiens ZW10 interactor (ZWINT), transcript variant 3, mRNA. | | | ZWINT | | | -1.25 | | | | | | | 2.53 | |
| Homo sapiens hyaluronan-mediated motility receptor (RHAMM) (HMMR), transcript variant 1, mRNA. | | | HMMR | | | -1.25 | | | | | | | 1.85 | |
| Homo sapiens SPC25, NDC80 kinetochore complex component, homolog (S. cerevisiae) (SPC25), mRNA. | | | SPC25 | | | -1.25 | | | | | | | 3.61 | |
| Homo sapiens COMM domain containing 4 (COMMD4), mRNA. | | | COMMD4 | | | -1.25 | | | | | | | 2.53 | |
| Homo sapiens family with sequence similarity 110, member B (FAM110B), mRNA. | | | FAM110B | | | -1.25 | | | | | | | 2.53 | |
| Homo sapiens proline/serine-rich coiled-coil 1 (PSRC1), transcript variant 3, mRNA. | | | PSRC1 | | | -1.25 | | | | | | | 1.85 | |
| Homo sapiens debranching enzyme homolog 1 (S. cerevisiae) (DBR1), mRNA. | | | DBR1 | | | -1.25 | | | | | | | 1.85 | |
| Homo sapiens cytoskeleton associated protein 2-like (CKAP2L), mRNA. | | | CKAP2L | | | -1.25 | | | | | | | 1.85 | |
| Homo sapiens mitogen-activated protein kinase 8 interacting protein 1 (MAPK8IP1), mRNA. | | | MAPK8IP1 | | | -1.25 | | | | | | | 3.61 | |
| Homo sapiens pleckstrin homology domain containing, family G (with RhoGef domain) member 3 (PLEKHG3), mRNA. | | | PLEKHG3 | | | -1.25 | | | | | | | 1.85 | |
| Homo sapiens DnaJ (Hsp40) homolog, subfamily A, member 1 (DNAJA1), mRNA. | | | DNAJA1 | | | -1.25 | | | | | | | 1.40 | |
| Homo sapiens KIAA1009 (KIAA1009), mRNA. | | | KIAA1009 | | | -1.25 | | | | | | | 1.05 | |
| Homo sapiens GS homeobox 2 (GSX2), mRNA. | | | GSX2 | | | -1.25 | | | | | | | 1.40 | |
| Homo sapiens polymerase (DNA directed), alpha 2 (70kD subunit) (POLA2), mRNA. | | | POLA2 | | | -1.25 | | | | | | | 2.53 | |
| Homo sapiens solute carrier family 2 (facilitated glucose transporter), member 6 (SLC2A6), mRNA. | | | SLC2A6 | | | -1.25 | | | | | | | 1.40 | |
| Homo sapiens forkhead box M1 (FOXM1), transcript variant 2, mRNA. | | | FOXM1 | | | -1.25 | | | | | | | 2.53 | |
| Homo sapiens histone cluster 1, H4c (HIST1H4C), mRNA. | | | HIST1H4C | | | -1.25 | | | | | | | 1.40 | |
| Homo sapiens PSMC3 interacting protein (PSMC3IP), transcript variant 2, mRNA. | | | PSMC3IP | | | -1.26 | | | | | | | 2.53 | |
| Homo sapiens tubulin, beta 3 (TUBB3), mRNA. | | | TUBB3 | | | -1.26 | | | | | | | 3.61 | |
| Homo sapiens chaperonin containing TCP1, subunit 6A (zeta 1) (CCT6A), transcript variant 2, mRNA. | | | CCT6A | | | -1.26 | | | | | | | 3.61 | |
| Homo sapiens chromosome 4 open reading frame 27 (C4orf27), mRNA. | | | C4ORF27 | | | -1.26 | | | | | | | 1.40 | |
| Homo sapiens pleckstrin homology domain containing, family A (phosphoinositide binding specific) member 4 (PLEKHA4), mRNA. | | | PLEKHA4 | | | -1.26 | | | | | | | 3.61 | |
| Homo sapiens synaptotagmin binding, cytoplasmic RNA interacting protein (SYNCRIP), mRNA. | | | SYNCRIP | | | -1.26 | | | | | | | 2.53 | |
| Homo sapiens hexokinase 2 (HK2), mRNA. | | | HK2 | | | -1.26 | | | | | | | 4.69 | |
| Homo sapiens tripartite motif-containing 24 (TRIM24), transcript variant 1, mRNA. | | | TRIM24 | | | -1.26 | | | | | | | 1.40 | |
| Homo sapiens mediator of DNA damage checkpoint 1 (MDC1), mRNA. | | | MDC1 | | | -1.26 | | | | | | | 1.40 | |
| Homo sapiens regulating synaptic membrane exocytosis 3 (RIMS3), mRNA. | | | RIMS3 | | | -1.26 | | | | | | | 2.53 | |
| Homo sapiens RAB7B, member RAS oncogene family (RAB7B), mRNA. | | | RAB7B | | | -1.26 | | | | | | | 1.85 | |
| Homo sapiens H2A histone family, member Z (H2AFZ), mRNA. | | | H2AFZ | | | -1.26 | | | | | | | 1.05 | |
| Homo sapiens LSM3 homolog, U6 small nuclear RNA associated (S. cerevisiae) (LSM3), mRNA. | | | LSM3 | | | -1.26 | | | | | | | 2.53 | |
| Homo sapiens coiled-coil domain containing 88A (CCDC88A), mRNA. | | | CCDC88A | | | -1.26 | | | | | | | 3.61 | |
| Homo sapiens frizzled homolog 2 (Drosophila) (FZD2), mRNA. | | | FZD2 | | | -1.26 | | | | | | | 1.40 | |
| Homo sapiens myeloid/lymphoid or mixed-lineage leukemia (trithorax homolog, Drosophila); translocated to, 11 (MLLT11), mRNA. | | | MLLT11 | | | -1.26 | | | | | | | 2.53 | |
| Homo sapiens stathmin 1 (STMN1), transcript variant 2, mRNA. | | | STMN1 | | | -1.26 | | | | | | | 2.53 | |
| Homo sapiens cell division cycle associated 8 (CDCA8), mRNA. | | | CDCA8 | | | -1.26 | | | | | | | 1.40 | |
| Homo sapiens carbonic anhydrase XIV (CA14), mRNA. | | | CA14 | | | -1.26 | | | | | | | 2.53 | |
| Homo sapiens chromosome 1 open reading frame 41 (C1orf41), mRNA. | | | C1ORF41 | | | -1.26 | | | | | | | 1.85 | |
| Homo sapiens guanine nucleotide binding protein (G protein), gamma 2 (GNG2), mRNA. | | | GNG2 | | | -1.26 | | | | | | | 2.53 | |
| Homo sapiens nuclear receptor coactivator 5 (NCOA5), mRNA. | | | NCOA5 | | | -1.26 | | | | | | | 1.40 | |
| Homo sapiens ribonucleotide reductase M1 polypeptide (RRM1), mRNA. | | | RRM1 | | | -1.26 | | | | | | | 1.40 | |
| Homo sapiens spermatid perinuclear RNA binding protein (STRBP), mRNA. | | | STRBP | | | -1.26 | | | | | | | 1.85 | |
| Homo sapiens tyrosine 3-monooxygenase/tryptophan 5-monooxygenase activation protein, eta polypeptide (YWHAH), mRNA. | | | YWHAH | | | -1.26 | | | | | | | 1.40 | |
| Homo sapiens acyl-Coenzyme A binding domain containing 7 (ACBD7), mRNA. | | | ACBD7 | | | -1.26 | | | | | | | 1.40 | |
| Homo sapiens kinesin family member 23 (KIF23), transcript variant 1, mRNA. | | | KIF23 | | | -1.26 | | | | | | | 2.53 | |
| Homo sapiens SEC16 homolog A (S. cerevisiae) (SEC16A), mRNA. | | | SEC16A | | | -1.26 | | | | | | | 1.40 | |
| Homo sapiens coiled-coil domain containing 99 (CCDC99), mRNA. | | | CCDC99 | | | -1.26 | | | | | | | 1.85 | |
| Homo sapiens RNA binding motif protein 12 (RBM12), transcript variant 1, mRNA. | | | RBM12 | | | -1.26 | | | | | | | 1.40 | |
| Homo sapiens carbohydrate (N-acetylgalactosamine 4-0) sulfotransferase 9 (CHST9), mRNA. | | | CHST9 | | | -1.26 | | | | | | | 1.40 | |
| Homo sapiens mannose-P-dolichol utilization defect 1 (MPDU1), mRNA. | | | MPDU1 | | | -1.26 | | | | | | | 2.53 | |
| Homo sapiens selenophosphate synthetase 1 (SEPHS1), mRNA. | | | SEPHS1 | | | -1.26 | | | | | | | 1.40 | |
| Homo sapiens hydrolethalus syndrome 1 (HYLS1), mRNA. | | | HYLS1 | | | -1.27 | | | | | | | 1.40 | |
| Homo sapiens RAD51 associated protein 1 (RAD51AP1), mRNA. | | | RAD51AP1 | | | -1.27 | | | | | | | 1.05 | |
| Homo sapiens cadherin, EGF LAG seven-pass G-type receptor 2 (flamingo homolog, Drosophila) (CELSR2), mRNA. | | | CELSR2 | | | -1.27 | | | | | | | 1.40 | |
| Homo sapiens protein kinase (cAMP-dependent, catalytic) inhibitor alpha (PKIA), transcript variant 6, mRNA. | | | PKIA | | | -1.27 | | | | | | | 2.53 | |
| Homo sapiens WD repeat domain 51A (WDR51A), mRNA. | | | WDR51A | | | -1.27 | | | | | | | 1.05 | |
| Homo sapiens polymerase (DNA directed), theta (POLQ), mRNA. | | | POLQ | | | -1.27 | | | | | | | 2.53 | |
| Homo sapiens MARCKS-like 1 (MARCKSL1), mRNA. | | | MARCKSL1 | | | -1.27 | | | | | | | 0.99 | |
| Homo sapiens zinc finger CCCH-type containing 4 (ZC3H4), mRNA. | | | ZC3H4 | | | -1.27 | | | | | | | 1.40 | |
| Homo sapiens nucleolar and spindle associated protein 1 (NUSAP1), transcript variant 2, mRNA. | | | NUSAP1 | | | -1.27 | | | | | | | 1.05 | |
| Homo sapiens ring finger protein, transmembrane 2 (RNFT2), transcript variant 2, mRNA. | | | RNFT2 | | | -1.27 | | | | | | | 1.40 | |
| Homo sapiens sphingosine-1-phosphate receptor 3 (S1PR3), mRNA. | | | S1PR3 | | | -1.27 | | | | | | | 1.85 | |
| Homo sapiens tweety homolog 1 (Drosophila) (TTYH1), transcript variant 1, mRNA. | | | TTYH1 | | | -1.27 | | | | | | | 1.05 | |
| Homo sapiens microtubule-associated protein 1A (MAP1A), mRNA. | | | MAP1A | | | -1.27 | | | | | | | 1.85 | |
| Homo sapiens dynein, cytoplasmic 1, intermediate chain 1 (DYNC1I1), mRNA. | | | DYNC1I1 | | | -1.27 | | | | | | | 1.05 | |
| Homo sapiens Protein HEG Homolog 1 (HEG1), mRNA. | | | HEG1 | | | -1.27 | | | | | | | 0.99 | |
| Homo sapiens NUF2, NDC80 kinetochore complex component, homolog (S. cerevisiae) (NUF2), transcript variant 2, mRNA. | | | NUF2 | | | -1.27 | | | | | | | 3.61 | |
| Homo sapiens achaete-scute complex homolog 1 (Drosophila) (ASCL1), mRNA. | | | ASCL1 | | | -1.27 | | | | | | | 2.53 | |
| Homo sapiens non-SMC condensin I complex, subunit D2 (NCAPD2), mRNA. | | | NCAPD2 | | | -1.27 | | | | | | | 2.53 | |
| Homo sapiens myosin, heavy chain 10, non-muscle (MYH10), mRNA. | | | MYH10 | | | -1.27 | | | | | | | 1.05 | |
| Homo sapiens tubulin, alpha 1a (TUBA1A), mRNA. | | | TUBA1A | | | -1.27 | | | | | | | 1.85 | |
| Homo sapiens chromatin assembly factor 1, subunit A (p150) (CHAF1A), mRNA. | | | CHAF1A | | | -1.27 | | | | | | | 1.85 | |
| Homo sapiens ATPase family, AAA domain containing 2 (ATAD2), mRNA. | | | ATAD2 | | | -1.27 | | | | | | | 1.85 | |
| Homo sapiens suppressor of variegation 3-9 homolog 1 (Drosophila) (SUV39H1), mRNA. | | | SUV39H1 | | | -1.27 | | | | | | | 1.85 | |
| Homo sapiens minichromosome maintenance complex component 5 (MCM5), mRNA. | | | MCM5 | | | -1.27 | | | | | | | 1.05 | |
| Homo sapiens transcription factor 7-like 1 (T-cell specific, HMG-box) (TCF7L1), mRNA. | | | TCF7L1 | | | -1.27 | | | | | | | 1.05 | |
| Homo sapiens phosphoglycerate mutase 2 (muscle) (PGAM2), mRNA. | | | PGAM2 | | | -1.28 | | | | | | | 1.85 | |
| Homo sapiens RAD54-like (S. cerevisiae) (RAD54L), mRNA. | | | RAD54L | | | -1.28 | | | | | | | 0.99 | |
| Homo sapiens BUB1 budding uninhibited by benzimidazoles 1 homolog (yeast) (BUB1), mRNA. | | | BUB1 | | | -1.28 | | | | | | | 1.85 | |
| Homo sapiens enhancer of zeste homolog 2 (Drosophila) (EZH2), transcript variant 1, mRNA. | | | EZH2 | | | -1.28 | | | | | | | 1.40 | |
| Homo sapiens SHC (Src homology 2 domain containing) transforming protein 3 (SHC3), mRNA. | | | SHC3 | | | -1.28 | | | | | | | 1.40 | |
| Homo sapiens discoidin domain receptor tyrosine kinase 2 (DDR2), transcript variant 2, mRNA. | | | DDR2 | | | -1.28 | | | | | | | 1.85 | |
| Homo sapiens kelch domain containing 8B (KLHDC8B), mRNA. | | | KLHDC8B | | | -1.28 | | | | | | | 1.05 | |
| Homo sapiens secretagogin, EF-hand calcium binding protein (SCGN), mRNA. | | | SCGN | | | -1.28 | | | | | | | 1.05 | |
| Homo sapiens MAD2 mitotic arrest deficient-like 1 (yeast) (MAD2L1), mRNA. | | | MAD2L1 | | | -1.28 | | | | | | | 0.99 | |
| Homo sapiens Ras association (RalGDS/AF-6) domain family member 2 (RASSF2), transcript variant 2, mRNA. | | | RASSF2 | | | -1.28 | | | | | | | 0.99 | |
| Homo sapiens replication factor C (activator 1) 4, 37kDa (RFC4), transcript variant 1, mRNA. | | | RFC4 | | | -1.28 | | | | | | | 1.05 | |
| Homo sapiens receptor accessory protein 1 (REEP1), mRNA. | | | REEP1 | | | -1.28 | | | | | | | 0.72 | |
| Homo sapiens chromosome 11 open reading frame 75 (C11orf75), mRNA. | | | C11ORF75 | | | -1.28 | | | | | | | 1.05 | |
| Homo sapiens Chromosome 9 Open Reading Frame 62 | | | C9ORF62 | | | -1.28 | | | | | | | 4.69 | |
| Homo sapiens tubulin, gamma 1 (TUBG1), mRNA. | | | TUBG1 | | | -1.28 | | | | | | | 1.40 | |
| Homo sapiens centromere protein M (CENPM), transcript variant 2, mRNA. | | | CENPM | | | -1.28 | | | | | | | 0.72 | |
| Homo sapiens chromosome 9 open reading frame 140 (C9orf140), mRNA. | | | C9ORF140 | | | -1.28 | | | | | | | 0.99 | |
| Homo sapiens zinc finger protein 618 (ZNF618), mRNA. | | | ZNF618 | | | -1.28 | | | | | | | 1.05 | |
| Homo sapiens centromere protein E, 312kDa (CENPE), mRNA. | | | CENPE | | | -1.28 | | | | | | | 0.72 | |
| Homo sapiens minichromosome maintenance complex component 7 (MCM7), transcript variant 1, mRNA. | | | MCM7 | | | -1.28 | | | | | | | 0.72 | |
| Homo sapiens ninein (GSK3B interacting protein) (NIN), transcript variant 1, mRNA. | | | NIN | | | -1.28 | | | | | | | 1.05 | |
| Homo sapiens fumarate hydratase (FH), nuclear gene encoding mitochondrial protein, mRNA. | | | FH | | | -1.28 | | | | | | | 1.40 | |
| Homo sapiens GDP-mannose pyrophosphorylase B (GMPPB), transcript variant 1, mRNA. | | | GMPPB | | | -1.28 | | | | | | | 1.05 | |
| Homo sapiens cytoskeleton associated protein 2 (CKAP2), mRNA. | | | CKAP2 | | | -1.28 | | | | | | | 0.99 | |
| Homo sapiens high-mobility group nucleosomal binding domain 2 (HMGN2), mRNA. | | | HMGN2 | | | -1.28 | | | | | | | 1.05 | |
| Homo sapiens transforming, acidic coiled-coil containing protein 3 (TACC3), mRNA. | | | TACC3 | | | -1.28 | | | | | | | 3.61 | |
| Homo sapiens Kruppel-like factor 13 (KLF13), mRNA. | | | KLF13 | | | -1.28 | | | | | | | 1.05 | |
| Homo sapiens baculoviral IAP repeat-containing 5 (BIRC5), transcript variant 1, mRNA. | | | BIRC5 | | | -1.28 | | | | | | | 1.05 | |
| Homo sapiens chromosome 13 open reading frame 37 (C13orf37), mRNA. | | | C13ORF37 | | | -1.28 | | | | | | | 0.72 | |
| Homo sapiens coiled-coil domain containing 74B (CCDC74B), mRNA. | | | CCDC74B | | | -1.29 | | | | | | | 0.99 | |
| Homo sapiens heterogeneous nuclear ribonucleoprotein M (HNRNPM), transcript variant 1, mRNA. | | | HNRNPM | | | -1.29 | | | | | | | 0.72 | |
| Homo sapiens apoptosis-inducing, TAF9-like domain 1 (APITD1), transcript variant B, mRNA. | | | APITD1 | | | -1.29 | | | | | | | 0.99 | |
| Homo sapiens kinesin family member 20A (KIF20A), mRNA. | | | KIF20A | | | -1.29 | | | | | | | 0.99 | |
| Homo sapiens Rac GTPase activating protein 1 (RACGAP1), mRNA. | | | RACGAP1 | | | -1.29 | | | | | | | 0.72 | |
| Homo sapiens pellino homolog 1 (Drosophila) (PELI1), mRNA. | | | PELI1 | | | -1.29 | | | | | | | 0.99 | |
| Homo sapiens RMI1, RecQ mediated genome instability 1, homolog (S. cerevisiae) (RMI1), mRNA. | | | RMI1 | | | -1.29 | | | | | | | 1.05 | |
| Homo sapiens metastasis associated 1 family, member 2 (MTA2), mRNA. | | | MTA2 | | | -1.29 | | | | | | | 0.99 | |
| Homo sapiens GINS complex subunit 3 (Psf3 homolog) (GINS3), mRNA. | | | GINS3 | | | -1.29 | | | | | | | 1.40 | |
| Homo sapiens minichromosome maintenance complex component 10 (MCM10), transcript variant 2, mRNA. | | | MCM10 | | | -1.29 | | | | | | | 1.85 | |
| Homo sapiens sperm autoantigenic protein 17 (SPA17), mRNA. | | | SPA17 | | | -1.29 | | | | | | | 0.99 | |
| Homo sapiens leucine zipper, down-regulated in cancer 1-like (LDOC1L), mRNA. | | | LDOC1L | | | -1.29 | | | | | | | 0.99 | |
| Homo sapiens cell division cycle 20 homolog (S. cerevisiae) (CDC20), mRNA. | | | CDC20 | | | -1.29 | | | | | | | 0.72 | |
| Homo sapiens lamin B2 (LMNB2), mRNA. | | | LMNB2 | | | -1.29 | | | | | | | 0.72 | |
| Homo sapiens BUB3 budding uninhibited by benzimidazoles 3 homolog (yeast) (BUB3), transcript variant 1, mRNA. | | | BUB3 | | | -1.29 | | | | | | | 0.99 | |
| Homo sapiens hexokinase domain containing 1 (HKDC1), mRNA. | | | HKDC1 | | | -1.29 | | | | | | | 0.72 | |
| Homo sapiens zinc finger protein 828 (ZNF828), mRNA. | | | ZNF828 | | | -1.29 | | | | | | | 0.99 | |
| Homo sapiens gamma-glutamyl hydrolase (conjugase, folylpolygammaglutamyl hydrolase) (GGH), mRNA. | | | GGH | | | -1.29 | | | | | | | 0.72 | |
| Homo sapiens polymerase (DNA directed), epsilon 2 (p59 subunit) (POLE2), mRNA. | | | POLE2 | | | -1.30 | | | | | | | 1.05 | |
| Homo sapiens leucine rich repeat containing 42 (LRRC42), mRNA. | | | LRRC42 | | | -1.30 | | | | | | | 0.72 | |
| Homo sapiens chromosome 1 open reading frame 106 (C1orf106), mRNA. | | | C1ORF106 | | | -1.30 | | | | | | | 0.99 | |
| Homo sapiens exonuclease 1 (EXO1), transcript variant 2, mRNA. | | | EXO1 | | | -1.30 | | | | | | | 0.99 | |
| Homo sapiens high-mobility group box 3 (HMGB3), mRNA. | | | HMGB3 | | | -1.30 | | | | | | | 0.99 | |
| Homo sapiens squalene epoxidase (SQLE), mRNA. | | | SQLE | | | -1.30 | | | | | | | 0.72 | |
| Homo sapiens myosin binding protein H-like (MYBPHL), mRNA. | | | MYBPHL | | | -1.30 | | | | | | | 0.72 | |
| Homo sapiens thymidylate synthetase (TYMS), mRNA. | | | TYMS | | | -1.30 | | | | | | | 0.72 | |
| Homo sapiens F-box protein 5 (FBXO5), mRNA. | | | FBXO5 | | | -1.30 | | | | | | | 1.40 | |
| Homo sapiens replication protein A3, 14kDa (RPA3), mRNA. | | | RPA3 | | | -1.30 | | | | | | | 0.72 | |
| Homo sapiens aurora kinase B (AURKB), mRNA. | | | AURKB | | | -1.30 | | | | | | | 0.72 | |
| Homo sapiens CHK1 checkpoint homolog (S. pombe) (CHEK1), mRNA. | | | CHEK1 | | | -1.30 | | | | | | | 0.72 | |
| Homo sapiens delta-like 1 (Drosophila) (DLL1), mRNA. | | | DLL1 | | | -1.30 | | | | | | | 0.72 | |
| Homo sapiens chromosome 6 open reading frame 173 (C6orf173), mRNA. | | | C6ORF173 | | | -1.30 | | | | | | | 0.72 | |
| Homo sapiens cache domain containing 1 (CACHD1), mRNA. | | | CACHD1 | | | -1.30 | | | | | | | 0.99 | |
| Homo sapiens isocitrate dehydrogenase 2 (NADP+), mitochondrial (IDH2), nuclear gene encoding mitochondrial protein, mRNA. | | | IDH2 | | | -1.30 | | | | | | | 0.72 | |
| Homo sapiens v-src sarcoma (Schmidt-Ruppin A-2) viral oncogene homolog (avian) (SRC), transcript variant 2, mRNA. | | | SRC | | | -1.30 | | | | | | | 0.72 | |
| Homo sapiens tubulin, beta 2A (TUBB2A), mRNA. | | | TUBB2A | | | -1.30 | | | | | | | 1.05 | |
| Homo sapiens chromosome 13 open reading frame 23 (C13orf23), transcript variant 1, mRNA. | | | C13ORF23 | | | -1.30 | | | | | | | 0.99 | |
| Homo sapiens DnaJ (Hsp40) homolog, subfamily C, member 9 (DNAJC9), mRNA. | | | DNAJC9 | | | -1.30 | | | | | | | 0.72 | |
| Homo sapiens caspase 2, apoptosis-related cysteine peptidase (CASP2), transcript variant 1, mRNA. | | | CASP2 | | | -1.30 | | | | | | | 0.72 | |
| Homo sapiens cyclin B2 (CCNB2), mRNA. | | | CCNB2 | | | -1.31 | | | | | | | 0.72 | |
| Homo sapiens E2F transcription factor 2 (E2F2), mRNA. | | | E2F2 | | | -1.31 | | | | | | | 0.72 | |
| Homo sapiens growth factor receptor-bound protein 10 (GRB10), transcript variant 4, mRNA. | | | GRB10 | | | -1.31 | | | | | | | 0.99 | |
| Homo sapiens centrosomal protein 55kDa (CEP55), mRNA. | | | CEP55 | | | -1.31 | | | | | | | 0.72 | |
| Homo sapiens TTK protein kinase (TTK), mRNA. | | | TTK | | | -1.31 | | | | | | | 0.72 | |
| Homo sapiens solute carrier family 35, member B4 (SLC35B4), mRNA. | | | SLC35B4 | | | -1.31 | | | | | | | 0.72 | |
| Homo sapiens glucosaminyl (N-acetyl) transferase 2, I-branching enzyme (GCNT2), transcript variant 1, mRNA. | | | GCNT2 | | | -1.31 | | | | | | | 0.72 | |
| Homo sapiens Sp8 transcription factor (SP8), transcript variant 1, mRNA. | | | SP8 | | | -1.31 | | | | | | | 0.72 | |
| Homo sapiens E2F transcription factor 7 (E2F7), mRNA. | | | E2F7 | | | -1.31 | | | | | | | 0.72 | |
| Homo sapiens G-2 and S-phase expressed 1 (GTSE1), mRNA. | | | GTSE1 | | | -1.31 | | | | | | | 0.72 | |
| Homo sapiens shugoshin-like 2 (S. pombe) (SGOL2), mRNA. | | | SGOL2 | | | -1.31 | | | | | | | 0.72 | |
| Homo sapiens thymopoietin (TMPO), transcript variant 2, mRNA. | | | TMPO | | | -1.31 | | | | | | | 0.72 | |
| Homo sapiens leucine rich repeat neuronal 1 (LRRN1), mRNA. | | | LRRN1 | | | -1.31 | | | | | | | 1.85 | |
| Homo sapiens structural maintenance of chromosomes 4 (SMC4), transcript variant 2, mRNA. | | | SMC4 | | | -1.31 | | | | | | | 0.72 | |
| Homo sapiens timeless homolog (Drosophila) (TIMELESS), mRNA. | | | TIMELESS | | | -1.31 | | | | | | | 0.72 | |
| Homo sapiens MLF1 interacting protein (MLF1IP), mRNA. | | | MLF1IP | | | -1.31 | | | | | | | 0.72 | |
| Homo sapiens ribosomal protein L39-like (RPL39L), mRNA. | | | RPL39L | | | -1.31 | | | | | | | 0.99 | |
| Homo sapiens protein regulator of cytokinesis 1 (PRC1), transcript variant 2, mRNA. | | | PRC1 | | | -1.31 | | | | | | | 0.72 | |
| Homo sapiens chromatin assembly factor 1, subunit B (p60) (CHAF1B), mRNA. | | | CHAF1B | | | -1.31 | | | | | | | 0.72 | |
| Homo sapiens nuclear autoantigenic sperm protein (histone-binding) (NASP), transcript variant 2, mRNA. | | | NASP | | | -1.31 | | | | | | | 0.72 | |
| Homo sapiens acetyl-Coenzyme A acetyltransferase 2 (ACAT2), mRNA. | | | ACAT2 | | | -1.32 | | | | | | | 0.72 | |
| Homo sapiens THO complex 4 (THOC4), mRNA. | | | THOC4 | | | -1.32 | | | | | | | 0.99 | |
| Homo sapiens paternally expressed 10 (PEG10), transcript variant 1, mRNA. | | | PEG10 | | | -1.32 | | | | | | | 1.85 | |
| Homo sapiens cyclin E2 (CCNE2), mRNA. | | | CCNE2 | | | -1.32 | | | | | | | 1.05 | |
| Homo sapiens plexin A2 (PLXNA2), mRNA. | | | PLXNA2 | | | -1.32 | | | | | | | 0.72 | |
| Homo sapiens kinesin family member 15 (KIF15), mRNA. | | | KIF15 | | | -1.32 | | | | | | | 0.72 | |
| Homo sapiens peptidylprolyl isomerase (cyclophilin)-like 5 (PPIL5), transcript variant 1, mRNA. | | | PPIL5 | | | -1.32 | | | | | | | 0.72 | |
| Homo sapiens transmembrane and tetratricopeptide repeat containing 2 (TMTC2), mRNA. | | | TMTC2 | | | -1.32 | | | | | | | 0.72 | |
| Homo sapiens secreted frizzled-related protein 1 (SFRP1), mRNA. | | | SFRP1 | | | -1.32 | | | | | | | 0.72 | |
| Homo sapiens DEK oncogene (DNA binding) (DEK), mRNA. | | | DEK | | | -1.32 | | | | | | | 1.05 | |
| Homo sapiens zinc finger and SCAN domain containing 16 (ZSCAN16), mRNA. | | | ZSCAN16 | | | -1.32 | | | | | | | 0.72 | |
| Homo sapiens kinesin family member 11 (KIF11), mRNA. | | | KIF11 | | | -1.32 | | | | | | | 0.99 | |
| Homo sapiens kinesin family member 4A (KIF4A), mRNA. | | | KIF4A | | | -1.32 | | | | | | | 0.72 | |
| Homo sapiens CDC45 cell division cycle 45-like (S. cerevisiae) (CDC45L), mRNA. | | | CDC45L | | | -1.32 | | | | | | | 0.72 | |
| Homo sapiens phosphoglucomutase 5 (PGM5), mRNA. | | | PGM5 | | | -1.32 | | | | | | | 0.72 | |
| Homo sapiens aurora kinase A (AURKA), transcript variant 3, mRNA. | | | AURKA | | | -1.32 | | | | | | | 0.72 | |
| Homo sapiens neural precursor cell expressed, developmentally down-regulated 4-like (NEDD4L), mRNA. | | | NEDD4L | | | -1.33 | | | | | | | 0.72 | |
| Homo sapiens doublecortin-like kinase 2 (DCLK2), transcript variant 1, mRNA. | | | DCLK2 | | | -1.33 | | | | | | | 0.72 | |
| Homo sapiens kinesin family member 22 (KIF22), mRNA. | | | KIF22 | | | -1.33 | | | | | | | 0.72 | |
| Homo sapiens chromobox homolog 2 (Pc class homolog, Drosophila) (CBX2), transcript variant 1, mRNA. | | | CBX2 | | | -1.33 | | | | | | | 0.72 | |
| Homo sapiens cyclin A2 (CCNA2), mRNA. | | | CCNA2 | | | -1.33 | | | | | | | 1.05 | |
| Homo sapiens stannin (SNN), mRNA. | | | SNN | | | -1.33 | | | | | | | 0.99 | |
| Homo sapiens myelin transcription factor 1 (MYT1), mRNA. | | | MYT1 | | | -1.33 | | | | | | | 0.72 | |
| Homo sapiens structural maintenance of chromosomes 2 (SMC2), transcript variant 1, mRNA. | | | SMC2 | | | -1.33 | | | | | | | 0.72 | |
| Homo sapiens cyclin-dependent kinase 6 (CDK6), mRNA. | | | CDK6 | | | -1.33 | | | | | | | 0.72 | |
| Homo sapiens chromosome 3 open reading frame 58 (C3orf58), mRNA. | | | C3ORF58 | | | -1.33 | | | | | | | 0.72 | |
| Homo sapiens ubiquitin-conjugating enzyme E2C (UBE2C), transcript variant 5, mRNA. | | | UBE2C | | | -1.33 | | | | | | | 0.72 | |
| Homo sapiens CDC28 protein kinase regulatory subunit 2 (CKS2), mRNA. | | | CKS2 | | | -1.33 | | | | | | | 0.72 | |
| Homo sapiens RGM domain family, member A (RGMA), mRNA. | | | RGMA | | | -1.33 | | | | | | | 0.72 | |
| Homo sapiens SRY (sex determining region Y)-box 11 (SOX11), mRNA. | | | SOX11 | | | -1.33 | | | | | | | 0.72 | |
| Homo sapiens protocadherin 18 (PCDH18), mRNA. | | | PCDH18 | | | -1.34 | | | | | | | 0.72 | |
| Homo sapiens minichromosome maintenance complex component 2 (MCM2), mRNA. | | | MCM2 | | | -1.34 | | | | | | | 0.72 | |
| Homo sapiens chromosome 5 open reading frame 13 (C5orf13), mRNA. | | | C5ORF13 | | | -1.34 | | | | | | | 0.72 | |
| Homo sapiens chromosome 5 open reading frame 54 (C5orf54), mRNA. | | | C5ORF54 | | | -1.34 | | | | | | | 0.72 | |
| Homo sapiens non-SMC condensin I complex, subunit G (NCAPG), mRNA. | | | NCAPG | | | -1.34 | | | | | | | 0.72 | |
| Homo sapiens ubiquitin specific peptidase 1 (USP1), transcript variant 1, mRNA. | | | USP1 | | | -1.34 | | | | | | | 0.72 | |
| Homo sapiens cell division cycle associated 5 (CDCA5), mRNA. | | | CDCA5 | | | -1.35 | | | | | | | 0.72 | |
| Homo sapiens topoisomerase (DNA) II alpha 170kDa (TOP2A), mRNA. | | | TOP2A | | | -1.35 | | | | | | | 0.72 | |
| Homo sapiens Fanconi anemia, complementation group D2 (FANCD2), transcript variant 1, mRNA. | | | FANCD2 | | | -1.35 | | | | | | | 0.72 | |
| Homo sapiens GINS complex subunit 2 (Psf2 homolog) (GINS2), mRNA. | | | GINS2 | | | -1.35 | | | | | | | 0.72 | |
| Homo sapiens ectodermal-neural cortex (with BTB-like domain) (ENC1), mRNA. | | | ENC1 | | | -1.35 | | | | | | | 0.72 | |
| Homo sapiens leucine rich repeat containing 17 (LRRC17), transcript variant 1, mRNA. | | | LRRC17 | | | -1.35 | | | | | | | 0.72 | |
| Homo sapiens carboxypeptidase E (CPE), mRNA. | | | CPE | | | -1.35 | | | | | | | 0.72 | |
| Homo sapiens minichromosome maintenance complex component 4 (MCM4), transcript variant 2, mRNA. | | | MCM4 | | | -1.35 | | | | | | | 0.72 | |
| Homo sapiens WD repeat domain 34 (WDR34), mRNA. | | | WDR34 | | | -1.35 | | | | | | | 0.72 | |
| Homo sapiens SPARC related modular calcium binding 1 (SMOC1), transcript variant 1, mRNA. | | | SMOC1 | | | -1.36 | | | | | | | 0.72 | |
| Homo sapiens BTB (POZ) domain containing 17 (BTBD17), mRNA. | | | BTBD17 | | | -1.36 | | | | | | | 0.72 | |
| Homo sapiens homolog of rat pragma of Rnd2 (PRAGMIN), mRNA. | | | PRAGMIN | | | -1.36 | | | | | | | 0.72 | |
| Homo sapiens KIAA0101 (KIAA0101), transcript variant 2, mRNA. | | | KIAA0101 | | | -1.36 | | | | | | | 0.72 | |
| Homo sapiens protein phosphatase 1, regulatory (inhibitor) subunit 1C (PPP1R1C), mRNA. | | | PPP1R1C | | | -1.36 | | | | | | | 0.72 | |
| Homo sapiens brain abundant, membrane attached signal protein 1 (BASP1), mRNA. | | | BASP1 | | | -1.36 | | | | | | | 0.72 | |
| Homo sapiens schlafen family member 11 (SLFN11), mRNA. | | | SLFN11 | | | -1.36 | | | | | | | 0.72 | |
| Homo sapiens brevican (BCAN), transcript variant 2, mRNA. | | | BCAN | | | -1.36 | | | | | | | 0.72 | |
| Homo sapiens ubiquitin-conjugating enzyme E2T (putative) (UBE2T), mRNA. | | | UBE2T | | | -1.36 | | | | | | | 0.72 | |
| Homo sapiens centromere protein V (CENPV), mRNA. | | | CENPV | | | -1.36 | | | | | | | 0.72 | |
| Homo sapiens cell division cycle 25 homolog A (S. pombe) (CDC25A), transcript variant 1, mRNA. | | | CDC25A | | | -1.36 | | | | | | | 0.72 | |
| Homo sapiens cell division cycle associated 3 (CDCA3), mRNA. | | | CDCA3 | | | -1.36 | | | | | | | 0.72 | |
| Homo sapiens myosin VC (MYO5C), mRNA. | | | MYO5C | | | -1.36 | | | | | | | 0.72 | |
| Homo sapiens flap structure-specific endonuclease 1 (FEN1), mRNA. | | | FEN1 | | | -1.37 | | | | | | | 0.72 | |
| Homo sapiens REST corepressor 2 (RCOR2), mRNA. | | | RCOR2 | | | -1.37 | | | | | | | 0.72 | |
| Homo sapiens cyclin F (CCNF), mRNA. | | | CCNF | | | -1.37 | | | | | | | 0.72 | |
| Homo sapiens thymidine kinase 1, soluble (TK1), mRNA. | | | TK1 | | | -1.37 | | | | | | | 0.72 | |
| Homo sapiens H2A histone family, member X (H2AFX), mRNA. | | | H2AFX | | | -1.37 | | | | | | | 0.72 | |
| Homo sapiens protein tyrosine phosphatase, receptor type, D (PTPRD), transcript variant 2, mRNA. | | | PTPRD | | | -1.37 | | | | | | | 0.72 | |
| Homo sapiens high-mobility group box 2 (HMGB2), mRNA. | | | HMGB2 | | | -1.37 | | | | | | | 0.72 | |
| Homo sapiens Opa interacting protein 5 (OIP5), mRNA. | | | OIP5 | | | -1.37 | | | | | | | 0.72 | |
| Homo sapiens SRY (sex determining region Y)-box 4 (SOX4), mRNA. | | | SOX4 | | | -1.37 | | | | | | | 0.72 | |
| Homo sapiens discs, large (Drosophila) homolog-associated protein 5 (DLGAP5), mRNA. | | | DLGAP5 | | | -1.37 | | | | | | | 0.72 | |
| Homo sapiens tetraspanin 12 (TSPAN12), mRNA. | | | TSPAN12 | | | -1.38 | | | | | | | 0.72 | |
| Homo sapiens doublecortin (DCX), transcript variant 4, mRNA. | | | DCX | | | -1.38 | | | | | | | 0.72 | |
| Homo sapiens centromere protein A (CENPA), transcript variant 2, mRNA. | | | CENPA | | | -1.38 | | | | | | | 0.72 | |
| Homo sapiens heterogeneous nuclear ribonucleoprotein A/B (HNRNPAB), transcript variant 1, mRNA. | | | HNRNPAB | | | -1.38 | | | | | | | 0.72 | |
| Homo sapiens cornichon homolog 2 (Drosophila) (CNIH2), mRNA. | | | CNIH2 | | | -1.39 | | | | | | | 0.72 | |
| Homo sapiens proliferating cell nuclear antigen (PCNA), transcript variant 2, mRNA. | | | PCNA | | | -1.40 | | | | | | | 0.72 | |
| Homo sapiens vaccinia related kinase 1 (VRK1), mRNA. | | | VRK1 | | | -1.40 | | | | | | | 0 | |
| Homo sapiens growth arrest and DNA-damage-inducible, gamma (GADD45G), mRNA. | | | GADD45G | | | -1.40 | | | | | | | 0.72 | |
| Homo sapiens family with sequence similarity 83, member D (FAM83D), mRNA. | | | FAM83D | | | -1.41 | | | | | | | 0.72 | |
| Homo sapiens maternal embryonic leucine zipper kinase (MELK), mRNA. | | | MELK | | | -1.41 | | | | | | | 0.72 | |
| Homo sapiens lipase, endothelial (LIPG), mRNA. | | | LIPG | | | -1.41 | | | | | | | 0.72 | |
| Homo sapiens retinol binding protein 1, cellular (RBP1), mRNA. | | | RBP1 | | | -1.41 | | | | | | | 0.72 | |
| Homo sapiens insulinoma-associated 1 (INSM1), mRNA. | | | INSM1 | | | -1.42 | | | | | | | 0.72 | |
| Homo sapiens Golgi-localized protein (GOLSYN), transcript variant 7, mRNA. | | | GOLSYN | | | -1.42 | | | | | | | 0.72 | |
| Homo sapiens MFNG O-fucosylpeptide 3-beta-N-acetylglucosaminyltransferase (MFNG), mRNA. | | | MFNG | | | -1.42 | | | | | | | 0.72 | |
| Homo sapiens neuronatin (NNAT), transcript variant 2, mRNA. | | | NNAT | | | -1.42 | | | | | | | 0.72 | |
| Homo sapiens cell division cycle 7 homolog (S. cerevisiae) (CDC7), mRNA. | | | CDC7 | | | -1.43 | | | | | | | 0.72 | |
| Homo sapiens rhomboid, veinlet-like 3 (Drosophila) (RHBDL3), mRNA. | | | RHBDL3 | | | -1.44 | | | | | | | 0 | |
| Homo sapiens distal-less homeobox 5 (DLX5), mRNA. | | | DLX5 | | | -1.45 | | | | | | | 0 | |
| Homo sapiens karyopherin alpha 2 (RAG cohort 1, importin alpha 1) (KPNA2), mRNA. XM_001133262 XM_001133265 XM_001133267 XM_001133271 | | | KPNA2 | | | -1.45 | | | | | | | 0 | |
| Homo sapiens transgelin 3 (TAGLN3), transcript variant 3, mRNA. | | | TAGLN3 | | | -1.45 | | | | | | | 0 | |
| Homo sapiens delta-like 3 (Drosophila) (DLL3), transcript variant 1, mRNA. | | | DLL3 | | | -1.47 | | | | | | | 0 | |
| Homo sapiens hairy and enhancer of split 5 (Drosophila) (HES5), mRNA. | | | HES5 | | | -1.61 | | | | | | | 0 | |
|  | | | | | | | | | | | | | | |
| **Gene Name** | | | **EtOH vs Cortisol__DD vs DC** | | **Fold Change**  **(EtOH vs Cortisol)** | **q value**  **(EtOH vs Cortisol) (%)** | | | **Fold Change**  **(DD vs DC)** | | **q value**  **(DD vs DC) (%)** | | | |
| Homo sapiens chromosome 14 open reading frame 37 (C14orf37), mRNA. | | | C14ORF37 | | -1.30 | 4.66 | | | -1.27 | | 2.46 | | | |
|  | | | | | | | | | | | | | | |
| **Gene Name** | **DD vs DC__DD vs DDC** | | | | **Fold Change**  **(DD vs DC)** | | **q value**  **(DD vs DC) (%)** | | | **Fold Change (DD vs DDC)** | **q value**  **(DD vs DDC) (%)** | | | |
| Homo sapiens VGF nerve growth factor inducible (VGF), mRNA. | VGF | | | | 2.05 | | 0 | | | 2.78 | 0 | | | |
| Homo sapiens proline dehydrogenase (oxidase) 1 (PRODH), nuclear gene encoding mitochondrial protein, mRNA. | PRODH | | | | 1.79 | | 0 | | | 1.35 | 1.40 | | | |
| Homo sapiens sterol regulatory element binding transcription factor 1 (SREBF1), transcript variant 1, mRNA. | SREBF1 | | | | 1.74 | | 0 | | | 1.42 | 0.70 | | | |
| Homo sapiens RNA, 5S ribosomal 9 (RN5S9), ribosomal RNA. | RN5S9 | | | | 1.72 | | 0 | | | 1.67 | 0 | | | |
| Homo sapiens RAS-like, family 10, member A (RASL10A), transcript variant 2, mRNA. | RASL10A | | | | 1.71 | | 0 | | | 1.79 | 0 | | | |
| Homo sapiens tumor necrosis factor receptor superfamily, member 21 (TNFRSF21), mRNA. | TNFRSF21 | | | | 1.62 | | 1.27 | | | 1.65 | 0.34 | | | |
| Homo sapiens LEM domain containing 1 (LEMD1), mRNA. | LEMD1 | | | | 1.61 | | 2.46 | | | 1.39 | 1.85 | | | |
| Homo sapiens tsukushin (TSKU), mRNA. | TSKU | | | | 1.57 | | 0 | | | 1.38 | 1.05 | | | |
| Homo sapiens asparagine synthetase (ASNS), transcript variant 1, mRNA. | ASNS | | | | 1.55 | | 0 | | | 1.31 | 4.69 | | | |
| Homo sapiens family with sequence similarity 107, member A (FAM107A), transcript variant 2, mRNA. | FAM107A | | | | 1.54 | | 0.74 | | | 1.59 | 0 | | | |
| Homo sapiens poly (ADP-ribose) polymerase family, member 8 (PARP8), mRNA. | PARP8 | | | | 1.54 | | 0 | | | 1.38 | 0.70 | | | |
| Homo sapiens COBL-like 1 (COBLL1), mRNA. | COBLL1 | | | | 1.53 | | 0 | | | 1.49 | 0.34 | | | |
| Homo sapiens metallothionein E (MTE), mRNA. | MTE | | | | 1.51 | | 0 | | | 1.42 | 0.34 | | | |
| Homo sapiens GrpE-like 1, mitochondrial (E. coli) (GRPEL1), nuclear gene encoding mitochondrial protein, mRNA. | GRPEL1 | | | | 1.50 | | 0 | | | 1.50 | 0.34 | | | |
| Homo sapiens mucolipin 2 (MCOLN2), mRNA. | MCOLN2 | | | | 1.50 | | 0 | | | 1.41 | 1.05 | | | |
| Homo sapiens hydroxyacylglutathione hydrolase-like (HAGHL), transcript variant 1, mRNA. | HAGHL | | | | 1.49 | | 1.27 | | | 1.39 | 0.99 | | | |
| Homo sapiens Ras association (RalGDS/AF-6) domain family member 4 (RASSF4), mRNA. | RASSF4 | | | | 1.49 | | 0.74 | | | 1.42 | 0.70 | | | |
| Homo sapiens matrix metallopeptidase 28 (MMP28), transcript variant 3, mRNA. | MMP28 | | | | 1.49 | | 0 | | | 1.68 | 0 | | | |
| Homo sapiens MAP kinase interacting serine/threonine kinase 2 (MKNK2), transcript variant 2, mRNA. | MKNK2 | | | | 1.48 | | 1.27 | | | 1.27 | 4.69 | | | |
| Homo sapiens 5',3'-nucleotidase, mitochondrial (NT5M), nuclear gene encoding mitochondrial protein, mRNA. | NT5M | | | | 1.41 | | 1.27 | | | 1.35 | 2.53 | | | |
| Homo sapiens solute carrier family 16, member 9 (monocarboxylic acid transporter 9) (SLC16A9), mRNA. | SLC16A9 | | | | 1.40 | | 2.46 | | | 1.29 | 2.53 | | | |
| Homo sapiens diacylglycerol kinase, delta 130kDa (DGKD), transcript variant 1, mRNA. | DGKD | | | | 1.36 | | 2.46 | | | 1.33 | 1.05 | | | |
| Homo sapiens calcium channel, voltage-dependent, T type, alpha 1H subunit (CACNA1H), transcript variant 2, mRNA. | CACNA1H | | | | 1.33 | | 2.46 | | | 1.42 | 0.99 | | | |
| Homo sapiens chromosome 10 open reading frame 140 (C10orf140), mRNA. | C10ORF140 | | | | -1.24 | | 1.69 | | | -1.44 | 0 | | | |
| Homo sapiens Kallmann syndrome 1 sequence (KAL1), mRNA. | KAL1 | | | | -1.25 | | 1.69 | | | -1.30 | 0.72 | | | |
| Homo sapiens family with sequence similarity 111, member A (FAM111A), transcript variant 1, mRNA. | FAM111A | | | | -1.25 | | 0 | | | -1.26 | 2.53 | | | |
| Homo sapiens signal-induced proliferation-associated 1 like 2 (SIPA1L2), mRNA. | SIPA1L2 | | | | -1.25 | | 1.69 | | | -1.21 | 3.61 | | | |
| Homo sapiens ATP-binding cassette, sub-family C, member 6 pseudogene 1 (ABCC6P1), non-coding RNA. | ABCC6P1 | | | | -1.25 | | 1.27 | | | -1.22 | 4.69 | | | |
| Homo sapiens HLA complex P5 (HCP5), mRNA. | HCP5 | | | | -1.26 | | 0.74 | | | -1.27 | 0.99 | | | |
| Homo sapiens CAP, adenylate cyclase-associated protein, 2 (yeast) (CAP2), mRNA. | CAP2 | | | | -1.26 | | 1.27 | | | -1.24 | 1.85 | | | |
| Homo sapiens centromere protein F, 350/400ka (mitosin) (CENPF), mRNA. | CENPF | | | | -1.27 | | 0 | | | -1.30 | 0.72 | | | |
| Homo sapiens LY6/PLAUR domain containing 6 (LYPD6), mRNA. | LYPD6 | | | | -1.27 | | 0.74 | | | -1.25 | 1.40 | | | |
| Homo sapiens vang-like 2 (van gogh, Drosophila) (VANGL2), mRNA. | VANGL2 | | | | -1.27 | | 1.69 | | | -1.23 | 2.53 | | | |
| Homo sapiens Meis homeobox 2 (MEIS2), transcript variant f, mRNA. | MEIS2 | | | | -1.28 | | 0 | | | -1.32 | 0.72 | | | |
| Homo sapiens histidine decarboxylase (HDC), mRNA. | HDC | | | | -1.28 | | 2.46 | | | -1.22 | 4.69 | | | |
| Homo sapiens phospholipase D family, member 5 (PLD5), mRNA. | PLD5 | | | | -1.28 | | 1.27 | | | -1.35 | 0.72 | | | |
| Homo sapiens NIMA (never in mitosis gene a)- related kinase 11 (NEK11), transcript variant 2, mRNA. | NEK11 | | | | -1.28 | | 0.74 | | | -1.27 | 1.40 | | | |
| Homo sapiens dynein, axonemal, light intermediate chain 1 (DNALI1), mRNA. | DNALI1 | | | | -1.28 | | 0.74 | | | -1.26 | 1.40 | | | |
| Homo sapiens neurocalcin delta (NCALD), mRNA. | NCALD | | | | -1.28 | | 0 | | | -1.28 | 0.72 | | | |
| Homo sapiens cryptochrome 1 (photolyase-like) (CRY1), mRNA. | CRY1 | | | | -1.29 | | 0.74 | | | -1.28 | 0.99 | | | |
| Homo sapiens lysyl oxidase (LOX), mRNA. | LOX | | | | -1.29 | | 1.69 | | | -1.27 | 0.72 | | | |
| Homo sapiens FYVE, RhoGEF and PH domain containing 3 (FGD3), transcript variant 2, mRNA. | FGD3 | | | | -1.30 | | 1.27 | | | -1.34 | 0.72 | | | |
| Homo sapiens chromosome 2 open reading frame 80 (C2orf80), mRNA. | C2ORF80 | | | | -1.30 | | 0 | | | -1.25 | 1.85 | | | |
| Homo sapiens xylosyltransferase I (XYLT1), mRNA. | XYLT1 | | | | -1.30 | | 0 | | | -1.29 | 0.72 | | | |
| Homo sapiens growth arrest-specific 7 (GAS7), transcript variant b, mRNA. | GAS7 | | | | -1.30 | | 0 | | | -1.34 | 0.72 | | | |
| Homo sapiens TOX high mobility group box family member 3 (TOX3), mRNA. | TOX3 | | | | -1.30 | | 0.74 | | | -1.23 | 4.69 | | | |
| Homo sapiens family with sequence similarity 46, member B (FAM46B), mRNA. | FAM46B | | | | -1.31 | | 0 | | | -1.31 | 0.72 | | | |
| Homo sapiens dimethylarginine dimethylaminohydrolase 1 (DDAH1), mRNA. | DDAH1 | | | | -1.32 | | 0 | | | -1.30 | 0.72 | | | |
| Homo sapiens microtubule-associated protein 1 light chain 3 gamma (MAP1LC3C), mRNA. | MAP1LC3C | | | | -1.32 | | 0 | | | -1.33 | 0.72 | | | |
| Homo sapiens phospholipase C, eta 1 (PLCH1), mRNA. | PLCH1 | | | | -1.32 | | 0.74 | | | -1.24 | 3.61 | | | |
| Homo sapiens actin-related protein 10 homolog (S. cerevisiae) (ACTR10), mRNA. | ACTR10 | | | | -1.33 | | 0 | | | -1.29 | 0.72 | | | |
| Homo sapiens follistatin-like 1 (FSTL1), mRNA. | FSTL1 | | | | -1.34 | | 0 | | | -1.33 | 0.72 | | | |
| Homo sapiens suppressor of cytokine signaling 2 (SOCS2), mRNA. | SOCS2 | | | | -1.34 | | 0 | | | -1.26 | 1.40 | | | |
| Homo sapiens radial spokehead-like 3 (RSHL3), mRNA. | RSHL3 | | | | -1.35 | | 0 | | | -1.47 | 0 | | | |
| Homo sapiens argininosuccinate synthetase 1 (ASS1), transcript variant 2, mRNA. | ASS1 | | | | -1.35 | | 0 | | | -1.37 | 0.72 | | | |
| Homo sapiens multimerin 1 (MMRN1), mRNA. | MMRN1 | | | | -1.35 | | 0 | | | -1.36 | 0.72 | | | |
| Homo sapiens C1q and tumor necrosis factor related protein 1 (C1QTNF1), transcript variant 2, mRNA. | C1QTNF1 | | | | -1.36 | | 0 | | | -1.25 | 2.53 | | | |
| Homo sapiens sema domain, seven thrombospondin repeats (type 1 and type 1-like), transmembrane domain (TM) and short cytoplasmic domain, (semaphorin) 5B (SEMA5B), transcript variant 1, mRNA. | SEMA5B | | | | -1.37 | | 1.69 | | | -1.34 | 0.72 | | | |
| Homo sapiens olfactomedin-like 2A (OLFML2A), mRNA. | OLFML2A | | | | -1.38 | | 0 | | | -1.33 | 0.72 | | | |
| Homo sapiens transforming growth factor, beta 2 (TGFB2), mRNA. | TGFB2 | | | | -1.38 | | 0 | | | -1.37 | 0.72 | | | |
| Homo sapiens solute carrier family 40 (iron-regulated transporter), member 1 (SLC40A1), mRNA. | SLC40A1 | | | | -1.40 | | 0 | | | -1.26 | 2.53 | | | |
| Homo sapiens seizure related 6 homolog (mouse) (SEZ6), transcript variant 2, mRNA. | SEZ6 | | | | -1.40 | | 2.46 | | | -1.25 | 2.53 | | | |
| Homo sapiens cysteine dioxygenase, type I (CDO1), mRNA. | CDO1 | | | | -1.41 | | 0 | | | -1.32 | 1.40 | | | |
| Homo sapiens ring finger protein 19A (RNF19A), transcript variant 1, mRNA. | RNF19A | | | | -1.42 | | 0 | | | -1.26 | 3.61 | | | |
| Homo sapiens engulfment and cell motility 1 (ELMO1), transcript variant 2, mRNA. | ELMO1 | | | | -1.42 | | 0 | | | -1.28 | 1.05 | | | |
| Homo sapiens cysteine-rich secretory protein LCCL domain containing 1 (CRISPLD1), mRNA. | CRISPLD1 | | | | -1.42 | | 0 | | | -1.25 | 1.85 | | | |
| Homo sapiens microRNA 1978 (MIR1978), microRNA. | MIR1978 | | | | -1.43 | | 0 | | | -1.30 | 1.85 | | | |
| Homo sapiens carbonic anhydrase VIII (CA8), mRNA. | CA8 | | | | -1.43 | | 0 | | | -1.36 | 0.72 | | | |
| Homo sapiens family with sequence similarity 84, member B (FAM84B), mRNA. | FAM84B | | | | -1.44 | | 0 | | | -1.25 | 1.40 | | | |
| Homo sapiens G protein-coupled receptor 177 (GPR177), transcript variant 2, mRNA. | GPR177 | | | | -1.45 | | 0 | | | -1.31 | 1.05 | | | |
| Homo sapiens arrestin domain containing 4 (ARRDC4), mRNA. | ARRDC4 | | | | -1.46 | | 0 | | | -1.33 | 0.72 | | | |
| Homo sapiens alpha-2-macroglobulin (A2M), mRNA. | A2M | | | | -1.48 | | 0 | | | -1.32 | 0.99 | | | |
| Homo sapiens alpha-kinase 2 (ALPK2), mRNA. | ALPK2 | | | | -1.57 | | 0 | | | -1.40 | 0.72 | | | |
| Homo sapiens synaptotagmin-like 2 (SYTL2), transcript variant b, mRNA. | SYTL2 | | | | -1.59 | | 0 | | | -1.32 | 0.72 | | | |
| Homo sapiens connective tissue growth factor (CTGF), mRNA. | CTGF | | | | -1.65 | | 0 | | | -1.30 | 0.69 | | | |
|  | | | | |  | | | | | | | | | |
| **Gene Name** | | **EtOH vs Cortisol__DD vs DDC** | | | **Fold Change**  **(EtOH vs Cortisol)** | | | **q value**  **(EtOH vs Cortisol) (%)** | | **Fold Change (DD vs DDC)** | | **q value**  **(DD vs DDC) (%)** | | |
| Homo sapiens ATP-binding cassette, sub-family C (CFTR/MRP), member 3 (ABCC3), mRNA. | | ABCC3 | | | 2.11 | | | 1.66 | | 2.74 | | 0 | | |
| Homo sapiens neuronal PAS domain protein 1 (NPAS1), mRNA. | | NPAS1 | | | 2.03 | | | 0 | | 2.26 | | 0 | | |
| Homo sapiens adrenomedullin (ADM), mRNA. | | ADM | | | 1.96 | | | 0 | | 2.15 | | 0 | | |
| Homo sapiens small nucleolar RNA, C/D box 3D (SNORD3D), small nucleolar RNA. | | SNORD3D | | | 1.88 | | | 3.93 | | 1.63 | | 0.34 | | |
| Homo sapiens sushi-repeat-containing protein, X-linked (SRPX), mRNA. | | SRPX | | | 1.86 | | | 1.66 | | 2.34 | | 0 | | |
| Homo sapiens delta-like 1 homolog (Drosophila) (DLK1), mRNA. | | DLK1 | | | 1.82 | | | 3.93 | | 2.06 | | 0 | | |
| Homo sapiens growth arrest and DNA-damage-inducible, alpha (GADD45A), mRNA. | | GADD45A | | | 1.80 | | | 1.66 | | 2.31 | | 0 | | |
| Homo sapiens zinc finger and BTB domain containing 16 (ZBTB16), transcript variant 1, mRNA. | | ZBTB16 | | | 1.79 | | | 0 | | 1.93 | | 0 | | |
| Homo sapiens nebulette (NEBL), transcript variant 2, mRNA. | | NEBL | | | 1.78 | | | 0 | | 1.68 | | 0 | | |
| Homo sapiens integrin, alpha 10 (ITGA10), mRNA. | | ITGA10 | | | 1.78 | | | 0 | | 1.34 | | 1.85 | | |
| Homo sapiens B-cell CLL/lymphoma 6 (zinc finger protein 51) (BCL6), transcript variant 2, mRNA. | | BCL6 | | | 1.74 | | | 1.66 | | 1.71 | | 0 | | |
| Homo sapiens cannabinoid receptor 1 (brain) (CNR1), transcript variant 1, mRNA. | | CNR1 | | | 1.68 | | | 0 | | 1.48 | | 0.70 | | |
| Homo sapiens phosphodiesterase 8B (PDE8B), transcript variant 3, mRNA. | | PDE8B | | | 1.62 | | | 0 | | 1.59 | | 0.34 | | |
| Homo sapiens neurofilament, light polypeptide 68kDa (NEFL), mRNA. | | NEFL | | | 1.61 | | | 1.66 | | 2.34 | | 0 | | |
| Homo sapiens v-myc myelocytomatosis viral oncogene homolog (avian) (MYC), mRNA. | | MYC | | | 1.60 | | | 0 | | 1.52 | | 0 | | |
| Homo sapiens neurofilament, medium polypeptide 150kDa (NEFM), mRNA. | | NEFM | | | 1.60 | | | 3.09 | | 1.57 | | 0.34 | | |
| Homo sapiens choline phosphotransferase 1 (CHPT1), mRNA. | | CHPT1 | | | 1.59 | | | 3.09 | | 1.35 | | 1.40 | | |
| Homo sapiens NUAK family, SNF1-like kinase, 1 (NUAK1), mRNA. | | NUAK1 | | | 1.54 | | | 0 | | 1.35 | | 1.85 | | |
| Homo sapiens neuron navigator 2 (NAV2), transcript variant 2, mRNA. | | NAV2 | | | 1.52 | | | 0 | | 1.66 | | 0 | | |
| Homo sapiens collagen, type XXIII, alpha 1 (COL23A1), mRNA. | | COL23A1 | | | 1.51 | | | 1.66 | | 1.37 | | 1.85 | | |
| Homo sapiens regulator of G-protein signaling 10 (RGS10), transcript variant 2, mRNA. | | RGS10 | | | 1.49 | | | 1.66 | | 1.33 | | 1.40 | | |
| Homo sapiens branched chain aminotransferase 2, mitochondrial (BCAT2), nuclear gene encoding mitochondrial protein, mRNA. | | BCAT2 | | | 1.47 | | | 3.93 | | 1.39 | | 1.05 | | |
| Homo sapiens DEAD/H-Box Helicase 12 | | DDX12 | | | 1.45 | | | 3.09 | | 1.33 | | 1.85 | | |
| Homo sapiens glycerol-3-phosphate dehydrogenase 1-like (GPD1L), mRNA. | | GPD1L | | | 1.37 | | | 1.66 | | 1.35 | | 1.05 | | |
| Homo sapiens parathyroid hormone 1 receptor (PTH1R), mRNA. | | PTH1R | | | 1.33 | | | 3.93 | | 1.30 | | 2.53 | | |
| Homo sapiens family with sequence similarity 64, member A (FAM64A), mRNA. | | FAM64A | | | -1.31 | | | 4.66 | | -1.34 | | 0.72 | | |
| Homo sapiens SH3-binding domain kinase 1 (SBK1), mRNA. | | SBK1 | | | -1.32 | | | 4.66 | | -1.34 | | 0.72 | | |
| Homo sapiens tubulin, beta (TUBB), mRNA. | | TUBB | | | -1.32 | | | 4.66 | | -1.37 | | 0.72 | | |
| Homo sapiens Rho family GTPase 3 (RND3), mRNA. | | RND3 | | | -1.34 | | | 4.66 | | -1.46 | | 0 | | |
| Homo sapiens B-cell CLL/lymphoma 11A (zinc finger protein) (BCL11A), transcript variant 1, mRNA. | | BCL11A | | | -1.34 | | | 4.66 | | -1.28 | | 0.72 | | |
| Homo sapiens kinesin family member C1 (KIFC1), mRNA. | | KIFC1 | | | -1.35 | | | 4.66 | | -1.26 | | 1.40 | | |
| Homo sapiens TEA domain family member 2 (TEAD2), mRNA. | | TEAD2 | | | -1.36 | | | 4.66 | | -1.33 | | 0.72 | | |
| Homo sapiens calpain 5 (CAPN5), mRNA. | | CAPN5 | | | -1.38 | | | 4.66 | | -1.28 | | 1.40 | | |
| Homo sapiens v-myc myelocytomatosis viral related oncogene, neuroblastoma derived (avian) (MYCN), mRNA. | | MYCN | | | -1.39 | | | 4.66 | | -1.28 | | 1.40 | | |
| Homo sapiens family with sequence similarity 181, member A (FAM181A), mRNA. | | FAM181A | | | -1.40 | | | 1.97 | | -1.38 | | 0.72 | | |
| Homo sapiens tubulin, beta 2C (TUBB2C), mRNA. | | TUBB2C | | | -1.40 | | | 0 | | -1.37 | | 0.72 | | |
| Homo sapiens synuclein, alpha interacting protein (SNCAIP), mRNA. | | SNCAIP | | | -1.40 | | | 4.66 | | -1.49 | | 0 | | |
| Homo sapiens collagen, type I, alpha 2 (COL1A2), mRNA. | | COL1A2 | | | -1.44 | | | 4.66 | | -1.49 | | 0 | | |
| Homo sapiens thymosin beta 15a (TMSB15A), mRNA. | | TMSB15A | | | -1.51 | | | 0 | | -1.48 | | 0 | | |
| Homo sapiens calbindin 2, 29kDa (calretinin) (CALB2), transcript variant CALB2c, mRNA. | | CALB2 | | | -1.59 | | | 4.66 | | -1.49 | | 0 | | |
|  | | | | | | | | | | | | | | |
| **Gene Name** | | **All conditions** | | **Fold Change (EtOH vs Cortisol)** | **q value**  **(EtOH vs Cortisol) (%)** | | | **Fold Change**  **(DD vs DC)** | | **q value**  **(DD vs DC) (%)** | | **Fold Change (DD vs DDC)** | | **q value**  **(DD vs DDC) (%)** |
| Homo sapiens metallothionein 3 (MT3), mRNA. | | MT3 | | 8.77 | 0 | | | 8.71 | | 0 | | 7.24 | | 0 |
| Homo sapiens TSC22 domain family, member 3 (TSC22D3), transcript variant 3, mRNA. | | TSC22D3 | | 5.24 | 0 | | | 2.76 | | 0 | | 3.50 | | 0 |
| Homo sapiens solute carrier organic anion transporter family, member 2A1 (SLCO2A1), mRNA. | | SLCO2A1 | | 4.71 | 0 | | | 4.55 | | 0 | | 4.47 | | 0 |
| Homo sapiens secreted phosphoprotein 1 (SPP1), transcript variant 1, mRNA. | | SPP1 | | 4.16 | 0 | | | 2.17 | | 0 | | 5.07 | | 0 |
| Homo sapiens receptor (G protein-coupled) activity modifying protein 1 (RAMP1), mRNA. | | RAMP1 | | 3.83 | 0 | | | 3.50 | | 0 | | 4.15 | | 0 |
| Homo sapiens metallothionein 2A (MT2A), mRNA. | | MT2A | | 3.77 | 0 | | | 3.00 | | 0 | | 3.46 | | 0 |
| Homo sapiens Kruppel-like factor 9 (KLF9), mRNA. | | KLF9 | | 3.20 | 0 | | | 2.74 | | 0 | | 3.19 | | 0 |
| Homo sapiens metallothionein 1A (MT1A), mRNA. | | MT1A | | 3.10 | 0 | | | 2.42 | | 0 | | 2.51 | | 0 |
| Homo sapiens aldehyde dehydrogenase 1 family, member L1 (ALDH1L1), mRNA. | | ALDH1L1 | | 2.85 | 0 | | | 2.42 | | 0 | | 2.93 | | 0 |
| Homo sapiens chromosome 13 open reading frame 15 (C13orf15), mRNA. | | C13ORF15 | | 2.20 | 0 | | | 2.44 | | 0 | | 2.05 | | 0 |
| Homo sapiens fibulin 1 (FBLN1), transcript variant C, mRNA. | | FBLN1 | | 2.14 | 0 | | | 1.81 | | 0 | | 2.40 | | 0 |
| Homo sapiens neuronal cell adhesion molecule (NRCAM), transcript variant 2, mRNA. | | NRCAM | | 1.95 | 0 | | | 1.85 | | 0.74 | | 2.37 | | 0 |
| Homo sapiens Bardet-Biedl syndrome 2 (BBS2), mRNA. | | BBS2 | | 1.87 | 0 | | | 1.54 | | 0.74 | | 1.60 | | 0 |
| Homo sapiens metallothionein 1X (MT1X), mRNA. | | MT1X | | 1.81 | 0 | | | 1.75 | | 0 | | 1.56 | | 0.34 |
| Homo sapiens solute carrier family 4, sodium bicarbonate cotransporter, member 4 (SLC4A4), mRNA. | | SLC4A4 | | 1.73 | 0 | | | 1.65 | | 0 | | 1.51 | | 0.34 |
| Homo sapiens cyclin D3 (CCND3), mRNA. | | CCND3 | | 1.70 | 0 | | | 1.62 | | 0 | | 2.03 | | 0 |
| Homo sapiens immunoglobulin superfamily, member 11 (IGSF11), transcript variant 2, mRNA. | | IGSF11 | | 1.67 | 3.93 | | | 1.38 | | 0.74 | | 1.34 | | 1.05 |
| Homo sapiens metallothionein 1G (MT1G), mRNA. | | MT1G | | 1.67 | 0 | | | 1.56 | | 0 | | 1.77 | | 0 |
| Homo sapiens aldolase C, fructose-bisphosphate (ALDOC), mRNA. | | ALDOC | | 1.67 | 1.66 | | | 1.77 | | 0 | | 1.49 | | 0.34 |
| Homo sapiens FK506 binding protein 5 (FKBP5), mRNA. | | FKBP5 | | 1.65 | 1.66 | | | 1.63 | | 0.74 | | 1.73 | | 0 |
| Homo sapiens hemoglobin, alpha 2 (HBA2), mRNA. | | HBA2 | | 1.59 | 0 | | | 1.67 | | 0 | | 1.65 | | 0 |
| Homo sapiens cystatin C (CST3), mRNA. | | CST3 | | 1.54 | 3.93 | | | 1.64 | | 0 | | 1.44 | | 0.34 |
| Homo sapiens interleukin 11 receptor, alpha (IL11RA), transcript variant 2, mRNA. | | IL11RA | | 1.54 | 3.09 | | | 1.57 | | 0 | | 1.46 | | 0.34 |
| Homo sapiens glutaredoxin (thioltransferase) (GLRX), mRNA. | | GLRX | | -1.32 | 4.66 | | | -1.35 | | 0.74 | | -1.31 | | 0.72 |
| Homo sapiens erythrocyte membrane protein band 4.1-like 3 (EPB41L3), mRNA. | | EPB41L3 | | -1.32 | 4.66 | | | -1.36 | | 0 | | -1.38 | | 0.72 |
| Homo sapiens ADAM metallopeptidase domain 19 (meltrin beta) (ADAM19), transcript variant 1, mRNA. | | ADAM19 | | -1.35 | 4.66 | | | -1.41 | | 0 | | -1.23 | | 4.69 |
| Homo sapiens MAM domain containing 2 (MAMDC2), mRNA. | | MAMDC2 | | -1.36 | 0 | | | -1.34 | | 1.69 | | -1.28 | | 0.99 |
| Homo sapiens chemokine (C-X-C motif) receptor 4 (CXCR4), transcript variant 2, mRNA. | | CXCR4 | | -1.39 | 4.66 | | | -1.33 | | 0.74 | | -1.43 | | 0.72 |
| Homo sapiens proteolipid protein 1 (PLP1), transcript variant 2, mRNA. | | PLP1 | | -1.39 | 4.66 | | | -1.30 | | 2.46 | | -1.24 | | 2.53 |
| Homo sapiens plasticity related gene 1 (LPPR4), mRNA. | | LPPR4 | | -1.40 | 4.66 | | | -1.34 | | 0 | | -1.36 | | 0.72 |
| Homo sapiens tenascin C (TNC), mRNA. | | TNC | | -1.40 | 4.66 | | | -1.43 | | 0 | | -1.28 | | 0.99 |
| Homo sapiens forkhead box J1 (FOXJ1), mRNA. | | FOXJ1 | | -1.42 | 4.66 | | | -1.44 | | 0 | | -1.53 | | 0 |
| Homo sapiens RALY RNA binding protein-like (RALYL), transcript variant 3, mRNA. | | RALYL | | -1.44 | 1.97 | | | -1.35 | | 0 | | -1.45 | | 0 |
| Homo sapiens calpain 9 (CAPN9), transcript variant 2, mRNA. | | CAPN9 | | -1.45 | 4.66 | | | -1.40 | | 0 | | -1.25 | | 1.85 |
| Homo sapiens contactin associated protein-like 2 (CNTNAP2), mRNA. | | CNTNAP2 | | -1.49 | 0 | | | -1.40 | | 0 | | -1.38 | | 0.72 |
| Homo sapiens chemokine (C-C motif) ligand 2 (CCL2), mRNA. | | CCL2 | | -1.50 | 0 | | | -1.64 | | 0 | | -1.32 | | 0.72 |
| Homo sapiens insulin-like growth factor binding protein 3 (IGFBP3), transcript variant 1, mRNA. | | IGFBP3 | | -1.56 | 4.66 | | | -1.43 | | 0 | | -1.29 | | 0.72 |
