# Supplementary material for: The role of omega-3 fatty acids in preventing glucocorticoid-induced reduction in human hippocampal neurogenesis and increase in apoptosis
Source: Transl Psychiatry. 2020 Jul 7;10:219. doi: 10.1038/s41398-020-00908-0 (PMC7341841; doi:10.1038/s41398-020-00908-0)
Supplement: Supplementary file 7 — Table S4 [file 41398_2020_908_MOESM7_ESM.docx]

**Table S4.** Signalling pathways modulated by cortisol, DHA alone and by DHA used in pre-treatment, and both pre- and co-treatment with cortisol.

| **EtOH vs Cortisol** |  |
| --- | --- |
| **Pathways** | **Molecules** |
| Agrin Interactions at Neuromuscular Junction | LAMB1 |
| CDK5 Signaling | LAMB1, PRSS23 |
| GP6 Signaling Pathway | LAMB1 |
|  | |
| **EtOH vs DD** |  |
| **Pathways** | **Molecules** |
| LPS/IL-1 Mediated Inhibition of RXR Function | CPT1A,HMGCS1,SREBF1 |
| Sucrose Degradation V (Mammalian) | ALDOC |
| Adipogenesis pathway | LPIN1,SREBF1 |
| Ketogenesis | HMGCS1 |
| Mevalonate Pathway I | HMGCS1 |
| Mitochondrial Dysfunction | CPT1A,SNCA |
| Hepatic Fibrosis / Hepatic Stellate Cell Activation | COL8A2,IGFBP5 |
| Superpathway of Geranylgeranyldiphosphate Biosynthesis I (via Mevalonate) | HMGCS1 |
| Parkinson's Signaling | SNCA |
| Mitochondrial L-carnitine Shuttle Pathway | CPT1A |
| Glutathione Redox Reactions I | GPX3 |
| Glycolysis I | ALDOC |
| Gluconeogenesis I | ALDOC |
| Superpathway of Cholesterol Biosynthesis | HMGCS1 |
| MIF-mediated Glucocorticoid Regulation | PLA2G3 |
| Sirtuin Signaling Pathway | CPT1A,SREBF1 |
| Neuroinflammation Signaling Pathway | PLA2G3,SNCA |
|  | |
| **DD vs DC** |  |
| **Pathways** | **Molecules** |
| Interferon Signaling | IFITM3 |
| FGF Signaling | FGFR3 |
| Bladder Cancer Signaling | FGFR3,MMP15,THBS1 |
| Inhibition of Angiogenesis by TSP1 | SDC2,THBS1 |
| Inhibition of Matrix Metalloproteases | MMP15,SDC2 |
| Spliceosomal Cycle | U2AF1/U2AF1L5 |
| L-carnitine Biosynthesis | BBOX1 |
| Granulocyte Adhesion and Diapedesis | MMP15,SDC2 |
| Wnt/β-catenin Signaling | FRZB,SFRP4 |
| B Cell Development | HLA-DRA |
| Fatty Acid β-oxidation I | ACAA2 |
| Coagulation System | F2R |
|  | |
| **DD vs DDC** |  |
| **Pathways** | **Molecules** |
| p53 Signaling | BIRC5,CHEK1,COQ8A,GADD45G,KAT2B,PCNA,PIK3R3,SCO2,SERPINE2 |
| NRF2-mediated Oxidative Stress Response | DNAJA1,DNAJB1,DNAJC9,ENC1,FTH1,GSTO1,GSTO2,MAP2K2,MGST1,PIK3R3,RASD1,RRAS,SQSTM1 |
| IL-1 Signaling | ADCY9,GNAT1,GNG2,GNG4,IRAK2,MAP3K14,NFKBIA,PRKAR2A |
| CREB Signaling in Neurons | ADCY9,CACNB2,CAMK2D,GNAT1,GNG2,GNG4,MAP2K2,PIK3R3,PLCD3,PRKAR2A,RASD1,RRAS,SHC1 |
| Cell Cycle Control of Chromosomal Replication | CDC45,CDC7,CDK1,CDK6,MCM2,MCM3,MCM4,MCM5,MCM6,MCM7,PCNA,POLA1,POLA2,RPA3,TOP2A |
| Mismatch Repair in Eukaryotes | EXO1,FEN1,PCNA,RFC2,RFC3,RFC4,RFC5 |
| Cyclins and Cell Cycle Regulation | CCNA2,CCNB1,CCNB2,CCNE2,CDC25A,CDK1,CDK6,CDKN2B,E2F2,E2F7,MYT1,SUV39H1,TFDP1,WEE1 |
| Cell Cycle: G2/M DNA Damage Checkpoint Regulation | AURKA,CCNB1,CCNB2,CDK1,CHEK1,CKS2,KAT2B,PPM1D,TOP2A,WEE1,YWHAH |
| Hereditary Breast Cancer Signaling | BLM,CCNB1,CDK1,CDK6,CHEK1,FANCC,FANCD2,GADD45G,H2AFX,PIK3R3,RASD1,RFC2,RFC3,RFC4,RFC5,RRAS,TUBG1,WEE1 |
| Molecular Mechanisms of Cancer | ADCY9,AURKA,BMP6,CAMK2D,CCNE2,CDC25A,CDK1,CDK6,CDKN2B,CHEK1,CYCS,DAXX,E2F2,E2F7,FANCD2,FOXO1,FZD2,GNAT1,ITGA3,ITGA5,MAP2K2,NFKBIA,PIK3R3,PRKAR2A,RASD1,RRAS,SHC1,SRC,SUV39H1,TCF3,TCF4,TFDP1,TGFBR2 |
| Mitotic Roles of Polo-Like Kinase | CCNB1,CCNB2,CDC20,CDC25A,CDC7,CDK1,FBXO5,KIF11,KIF23,PLK4,PRC1,WEE1 |
| Role of CHK Proteins in Cell Cycle Checkpoint Control | CDC25A,CDK1,CHEK1,E2F2,E2F7,MDC1,PCNA,RFC2,RFC3,RFC4,RFC5 |
| ATM Signaling | BLM,CCNB1,CCNB2,CDC25A,CDK1,CHEK1,FANCD2,GADD45G,H2AFX,MDC1,NFKBIA,PPM1D,SMC2,SUV39H1 |
| Glioma Signaling | CAMK2D,CDK6,CDKN2B,E2F2,E2F7,IDH1,IDH2,MAP2K2,PDGFA,PIK3R3,RASD1,RRAS,SHC1,SUV39H1,TFDP1 |
| Estrogen-mediated S-phase Entry | CCNA2,CCNE2,CDC25A,CDK1,E2F2,E2F7,TFDP1 |
| NER Pathway | CHAF1A,CHAF1B,HIST1H4C,PCNA,POLA1,POLA2,POLE2,RFC2,RFC3,RFC4,RFC5,RPA3,TOP2A |
| Breast Cancer Regulation by Stathmin1 | ADCY9,CAMK2D,CCNE2,CDK1,E2F2,E2F7,GNG2,GNG4,MAP2K2,PIK3R3,PRKAR2A,RASD1,RRAS,SHC1,STMN1,TUBA1A,TUBB2A,TUBB3,TUBG1 |
| Ovarian Cancer Signaling | CD44,EDN1,FZD2,MAP2K2,MMP7,PIK3R3,PRKAR2A,RASD1,RRAS,SRC,SUV39H1,TCF3,TCF4,TCF7L1,TFDP1 |
| Role of BRCA1 in DNA Damage Response | BLM,CHEK1,E2F2,E2F7,FANCC,FANCD2,MDC1,RFC2,RFC3,RFC4,RFC5 |
| 14-3-3-mediated Signaling | FOXO1,GFAP,MAP2K2,MAPT,PIK3R3,PLCD3,RASD1,RRAS,SRC,TUBA1A,TUBB2A,TUBB3,TUBG1,YWHAH |
| Adipogenesis pathway | AGPAT2,ATG7,CEBPD,CLOCK,EGR2,EZH2,FGFRL1,FOXO1,FZD2,KAT2A,KAT2B,RBBP4,RBP1,SAP30 |
| GADD45 Signaling | CCNB1,CCNE2,CDK1,GADD45G,PCNA |
| Cell Cycle: G1/S Checkpoint Regulation | CCNE2,CDC25A,CDK6,CDKN2B,E2F2,E2F7,FOXO1,SUV39H1,TFDP1 |
| PI3K/AKT Signaling | FOXO1,GDF15,ITGA3,ITGA5,MAP2K2,MAPK8IP1,NFKBIA,PIK3R3,RASD1,RRAS,SHC1,SYNJ2,YWHAH |
| Epithelial Adherens Junction Signaling | DLL1,MYH10,RASD1,RRAS,SORBS1,SRC,TCF3,TCF4,TCF7L1,TGFBR2,TUBA1A,TUBB2A,TUBB3,TUBG1 |
| IGF-1 Signaling | FOXO1,GRB10,IGFBP7,IRS2,MAP2K2,PIK3R3,PRKAR2A,RASD1,RRAS,SHC1,YWHAH |
| Ephrin Receptor Signaling | EFNA1,GNAT1,GNG2,GNG4,ITGA3,ITGA5,MAP2K2,MAP3K14,NCK2,PDGFA,RASD1,RRAS,SHC1,SORBS1,SRC |
| Glioblastoma Multiforme Signaling | CDK6,E2F2,E2F7,FOXO1,FZD2,MAP2K2,PDGFA,PIK3R3,PLCD3,RASD1,RRAS,SHC1,SRC,TCF3 |
| Regulation of Cellular Mechanics by Calpain Protease | CCNA2,CDK1,CDK6,ITGA3,ITGA5,RASD1,RRAS,SRC |
| Thyroid Cancer Signaling | MAP2K2,RASD1,RRAS,SHC1,TCF3,TCF4,TCF7L1 |
| PAK Signaling | ARHGAP10,ITGA3,ITGA5,MAP2K2,NCK2,PDGFA,PIK3R3,RASD1,RRAS,SHC1 |
| Germ Cell-Sertoli Cell Junction Signaling | GSN,ITGA3,MAP2K2,MAP3K14,PIK3R3,RASD1,RRAS,SORBS1,SRC,TGFBR2,TUBA1A,TUBB2A,TUBB3,TUBG1 |
| Aryl Hydrocarbon Receptor Signaling | CCNA2,CCNE2,CDK6,CHEK1,GSTO1,GSTO2,MCM7,MGST1,NFIB,POLA1,SRC,TFDP1 |
| Chronic Myeloid Leukemia Signaling | CDK6,E2F2,E2F7,MAP2K2,PIK3R3,RASD1,RRAS,SUV39H1,TFDP1,TGFBR2 |
| Mouse Embryonic Stem Cell Pluripotency | FZD2,ID1,ID3,MAP2K2,PIK3R3,RASD1,RRAS,TCF3,TCF4,TCF7L1 |
| PDGF Signaling | CAV1,MAP2K2,PDGFA,PIK3R3,RASD1,RRAS,SHC1,SRC,SYNJ2 |
| Axonal Guidance Signaling | ADAM15,BMP6,EFNA1,FZD2,GNAT1,GNG2,GNG4,ITGA3,ITGA5,MAP2K2,MMP7,NCK2,PDGFA,PFN2,PIK3R3,PLCD3,PLXNA2,PLXNB1,PRKAR2A,RASD1,RRAS,SEMA4D,SEMA6A,SHC1,TUBA1A,TUBB2A,TUBB3,TUBG1 |
| Leucine Degradation I | ACADM,BCAT1,HMGCL |
| Small Cell Lung Cancer Signaling | CCNE2,CDK6,CDKN2B,CYCS,NFKBIA,PIK3R3,SUV39H1,TFDP1 |
| Acute Myeloid Leukemia Signaling | IDH1,IDH2,MAP2K2,PIK3R3,RASD1,RRAS,TCF3,TCF4,TCF7L1 |
| Ascorbate Recycling (Cytosolic) | GSTO1,GSTO2 |
| DNA damage-induced 14-3-3σ Signaling | CCNB1,CCNB2,CCNE2,CDK1 |
| Prostate Cancer Signaling | CCNE2,FOXO1,MAP2K2,NFKBIA,PIK3R3,RASD1,RRAS,SUV39H1,TFDP1 |
| Gαi Signaling | ADCY9,CAV1,GNG2,GNG4,OPRL1,PRKAR2A,RASD1,RRAS,S1PR3,SHC1,SRC |
| PTEN Signaling | FOXO1,ITGA3,ITGA5,MAP2K2,PIK3R3,RASD1,RRAS,SHC1,SYNJ2,TGFBR2,YWHAH |
| Neuregulin Signaling | ERRFI1,ITGA3,ITGA5,MAP2K2,PIK3R3,RASD1,RRAS,SHC1,SRC |
| Melanocyte Development and Pigmentation Signaling | ADCY9,MAP2K2,PIK3R3,PRKAR2A,RASD1,RPS6KA2,RRAS,SHC1,SRC |
| Reelin Signaling in Neurons | DCX,ITGA3,ITGA5,MAPK8IP1,MAPT,PAFAH1B3,PIK3R3,SRC |
| Wnt/β-catenin Signaling | CD44,CDH5,FZD2,KREMEN2,MMP7,SFRP1,SOX11,SOX4,SRC,TCF3,TCF4,TCF7L1,TGFBR2 |
| Human Embryonic Stem Cell Pluripotency | BMP6,FGFRL1,FOXO1,FZD2,PDGFA,PIK3R3,S1PR3,TCF3,TCF4,TCF7L1,TGFBR2 |
| Role of NFAT in Cardiac Hypertrophy | ADCY9,CACNB2,CAMK2D,GNG2,GNG4,MAP2K2,PIK3R3,PLCD3,PRKAR2A,RASD1,RCAN1,RRAS,SHC1,SRC,TGFBR2 |
| Spermine and Spermidine Degradation I | SAT1,SMOX |
| Insulin Receptor Signaling | FOXO1,GRB10,IRS2,MAP2K2,PIK3R3,PRKAR2A,RASD1,RRAS,SGK1,SHC1,SYNJ2 |
| Gap Junction Signaling | ADCY9,CAV1,DBN1,MAP2K2,PIK3R3,PLCD3,PRKAR2A,RASD1,RRAS,SRC,TUBA1A,TUBB2A,TUBB3,TUBG1 |
| Role of Osteoblasts, Osteoclasts and Chondrocytes in Rheumatoid Arthritis | BMP6,DLX5,FOXO1,FZD2,GSN,ITGA3,ITGA5,MAP3K14,NFKBIA,PIK3R3,SFRP1,SRC,TCF3,TCF4,TCF7L1 |
| NF-κB Activation by Viruses | ITGA3,ITGA5,ITGAV,MAP3K14,NFKBIA,PIK3R3,RASD1,RRAS |
| Phagosome Maturation | ATP6V1E1,CTSC,CTSV,DYNC1I1,DYNLL1,LAMP2,RAB7B,TUBA1A,TUBB2A,TUBB3,TUBG1 |
| G Beta Gamma Signaling | CACNB2,CAV1,GNAT1,GNG2,GNG4,PRKAR2A,RASD1,RRAS,SHC1,SRC |
| Sertoli Cell-Sertoli Cell Junction Signaling | ITGA3,ITGA5,MAP2K2,MAP3K14,PRKAR2A,RASD1,RRAS,SORBS1,SRC,TUBA1A,TUBB2A,TUBB3,TUBG1 |
| Glutathione-mediated Detoxification | GGH,GSTO1,GSTO2,MGST1 |
| Endocannabinoid Cancer Inhibition Pathway | ADCY9,ATF3,CASP2,CCNE2,MAP2K2,PIK3R3,PRKAR2A,SRC,TCF3,TCF4,TCF7L1 |
| Colorectal Cancer Metastasis Signaling | ADCY9,BIRC5,FZD2,GNG2,GNG4,MAP2K2,MMP7,PIK3R3,PRKAR2A,RASD1,RRAS,SRC,TCF3,TCF4,TCF7L1,TGFBR2 |
| Paxillin Signaling | ITGA3,ITGA5,ITGAV,ITGB4,NCK2,PIK3R3,RASD1,RRAS,SRC |
| Colanic Acid Building Blocks Biosynthesis | GALK1,GMDS,GMPPB |
| Serine Biosynthesis | PHGDH,PSPH |
| Integrin Signaling | CAV1,GSN,ITGA3,ITGA5,ITGAV,ITGB4,MAP2K2,NCK2,PFN2,PIK3R3,RASD1,RRAS,SHC1,SRC |
| Protein Kinase A Signaling | ADCY9,AKAP12,CAMK2D,CDC25A,DUSP1,GNG2,GNG4,MAP2K2,MYH10,NFKBIA,PDE4C,PLCD3,PRKAR2A,PTPRD,PTPRE,PTPRG,PYGB,TCF3,TCF4,TCF7L1,TGFBR2,YWHAH |
| Pancreatic Adenocarcinoma Signaling | BIRC5,CDKN2B,E2F2,E2F7,MAP2K2,PIK3R3,SUV39H1,TFDP1,TGFBR2 |
| Non-Small Cell Lung Cancer Signaling | CDK6,MAP2K2,PIK3R3,RASD1,RRAS,SUV39H1,TFDP1 |
| Cancer Drug Resistance By Drug Efflux | ABCB1,FOXO1,MAP2K2,PIK3R3,RASD1,RRAS |
| Antiproliferative Role of Somatostatin Receptor 2 | GNG2,GNG4,MAP2K2,PIK3R3,RASD1,RRAS,SRC |
| α-Adrenergic Signaling | ADCY9,GNG2,GNG4,MAP2K2,PRKAR2A,PYGB,RASD1,RRAS |
| FAK Signaling | HMMR,ITGA3,ITGA5,MAP2K2,PIK3R3,RASD1,RRAS,SRC |
| Erythropoietin Signaling | MAP2K2,NFKBIA,PIK3R3,RASD1,RRAS,SHC1,SRC |
| Macropinocytosis Signaling | ITGA5,ITGB4,PDGFA,PIK3R3,RASD1,RRAS,SRC |
| Apoptosis Signaling | CASP2,CDK1,CYCS,MAP2K2,MAP3K14,NFKBIA,RASD1,RRAS |
| Acute Phase Response Signaling | MAP2K2,MAP3K14,NFKBIA,PIK3R3,RASD1,RBP1,RRAS,SERPINA3,SERPINE1,SHC1,TCF3,TCF4 |
| FAT10 Cancer Signaling Pathway | MAD2L1,NFKBIA,PCNA,TCF4,TGFBR2 |
| PFKFB4 Signaling Pathway | HK2,MAP2K2,PFKM,PRKAR2A,TKT |
| B Cell Receptor Signaling | CAMK2D,FOXO1,MAP2K2,MAP3K14,NFKBIA,PAG1,PIK3R3,RASD1,RRAS,SHC1,SYNJ2,TCF3 |
| Thrombopoietin Signaling | IRS2,MAP2K2,PIK3R3,RASD1,RRAS,SHC1 |
| Pyridoxal 5'-phosphate Salvage Pathway | CDK1,CDK6,GRK5,MAP2K2,SGK1,TTK |
| Endothelin-1 Signaling | ADCY9,CASP2,EDN1,GNAT1,PIK3R3,PLCD3,RARRES3,RASD1,RRAS,SHC1,SHC3,SRC |
| Superpathway of Serine and Glycine Biosynthesis I | PHGDH,PSPH |
| ErbB2-ErbB3 Signaling | FOXO1,MAP2K2,PIK3R3,RASD1,RRAS,SHC1 |
| Myc Mediated Apoptosis Signaling | CYCS,PIK3R3,RASD1,RRAS,SHC1,YWHAH |
| HER-2 Signaling in Breast Cancer | CCNE2,CDK6,FOXO1,ITGB4,PIK3R3,RASD1,RRAS |
| DNA Methylation and Transcriptional Repression Signaling | HIST1H4C,MTA2,RBBP4,SAP30 |
| Role of Macrophages, Fibroblasts and Endothelial Cells in Rheumatoid Arthritis | CAMK2D,CEBPD,FZD2,IRAK2,MAP2K2,MAP3K14,NFKBIA,PDGFA,PIK3R3,PLCD3,RASD1,RRAS,SFRP1,SRC,TCF3,TCF4,TCF7L1 |
| P2Y Purigenic Receptor Signaling Pathway | ADCY9,GNG2,GNG4,MAP2K2,PIK3R3,PLCD3,PRKAR2A,RASD1,RRAS |
| Virus Entry via Endocytic Pathways | CAV1,ITGA3,ITGA5,ITGB4,PIK3R3,RASD1,RRAS,SRC |
| CD27 Signaling in Lymphocytes | CYCS,MAP2K2,MAP3K14,NFKBIA,SIVA1 |
| p70S6K Signaling | MAP2K2,MAPT,PIK3R3,PLCD3,RASD1,RRAS,SHC1,SRC,YWHAH |
| Factors Promoting Cardiogenesis in Vertebrates | BMP6,CCNE2,FZD2,TCF3,TCF4,TCF7L1,TGFBR2 |
| GM-CSF Signaling | CAMK2D,MAP2K2,PIK3R3,RASD1,RRAS,SHC1 |
| Autophagy | ATG7,CTSC,CTSV,LAMP2,SQSTM1 |
| Glutamate Removal from Folates | GGH |
| ERK5 Signaling | RASD1,RPS6KA2,RRAS,SGK1,SRC,YWHAH |
| Transcriptional Regulatory Network in Embryonic Stem Cells | FOXC1,GSX2,HIST1H4C,TCF7L1,TRIM24 |
| Pyrimidine Deoxyribonucleotides De Novo Biosynthesis I | RRM1,RRM2,TYMS |
| Caveolar-mediated Endocytosis Signaling | CAV1,ITGA3,ITGA5,ITGAV,ITGB4,SRC |
| Glioma Invasiveness Signaling | CD44,HMMR,ITGAV,PIK3R3,RASD1,RRAS |
| Aldosterone Signaling in Epithelial Cells | CRYAB,DNAJA1,DNAJB1,DNAJC9,DUSP1,HSPH1,MAP2K2,PIK3R3,PLCD3,SGK1 |
| PI3K Signaling in B Lymphocytes | ATF3,CAMK2D,IRS2,MAP2K2,NFKBIA,PLCD3,PLEKHA4,RASD1,RRAS |
| Leptin Signaling in Obesity | ADCY9,FOXO1,MAP2K2,PIK3R3,PLCD3,PRKAR2A |
| PPARα/RXRα Activation | ADCY9,CLOCK,MAP2K2,MAP3K14,NFKBIA,PLCD3,PRKAR2A,RASD1,RRAS,SHC1,TGFBR2 |
| Androgen Signaling | CACNB2,DNAJB1,GNAT1,GNG2,GNG4,KAT2B,PRKAR2A,SHC1,SRC |
| ErbB Signaling | FOXO1,MAP2K2,NCK2,PIK3R3,RASD1,RRAS,SHC1 |
| Glutamate Receptor Signaling | GLS,GNG2,HOMER2,SLC1A2,SLC38A1 |
| Angiopoietin Signaling | BIRC5,FOXO1,NFKBIA,PIK3R3,RASD1,RRAS |
| G-Protein Coupled Receptor Signaling | ADCY9,ADORA2B,CAMK2D,DUSP1,MAP2K2,NFKBIA,OPRL1,PDE4C,PIK3R3,PRKAR2A,RASD1,RRAS,S1PR3,SHC1,SRC |
| Ketogenesis | ACAT2,HMGCL |
| Notch Signaling | DLL1,DLL3,HES5,HEY2,MFNG |
| CNTF Signaling | MAP2K2,PIK3R3,RASD1,RPS6KA2,RRAS |
| Endocannabinoid Developing Neuron Pathway | ADCY9,GNG2,MAP2K2,PIK3R3,PRKAR2A,RASD1,RRAS,SRC |
| Thrombin Signaling | ADCY9,CAMK2D,GNAT1,GNG2,GNG4,MAP2K2,PIK3R3,PLCD3,RASD1,RRAS,SHC1,SRC |
| GDNF Family Ligand-Receptor Interactions | IRS2,MAP2K2,PIK3R3,RASD1,RRAS,SHC1 |
| Glycolysis I | PFKM,PGAM2,PGAM4 |
| Renin-Angiotensin Signaling | ADCY9,MAP2K2,PIK3R3,PRKAR2A,RASD1,RRAS,SHC1,SHC3 |
| Role of NANOG in Mammalian Embryonic Stem Cell Pluripotency | BMP6,FZD2,MAP2K2,PIK3R3,RASD1,RRAS,SHC1,TCF7L1 |
| VEGF Signaling | FOXO1,MAP2K2,PIK3R3,RASD1,RRAS,SHC1,SRC |
| Osteoarthritis Pathway | CASP2,DDR2,DLX5,FZD2,ITGA3,ITGA5,PTHLH,S1PR3,TCF3,TCF4,TCF7L1,TGFBR2 |
|  | |
| **EtOH vs Cortisol__DD vs DC** |  |
| **Pathways** | **Molecules** |
| mTORC2 Signaling | C14ORF37 |
|  | |
| **EtOH vs Cortisol__DD vs DDC** |  |
| **Pathways** | **Molecules** |
| Cell Cycle: G1/S Checkpoint Regulation | MYC |
| Amyotrophic Lateral Sclerosis Signaling | CAPN5,NEFL,NEFM |
| cAMP-mediated signaling | CNR1,PDE8B,PTH1R,RGS10 |
| G-Protein Coupled Receptor Signaling | CNR1,PDE8B,PTH1R,RGS10 |
| Germ Cell-Sertoli Cell Junction Signaling | RND3,TUBB,TUBB4B |
| Integrin Signaling | CAPN5,ITGA10,RND3 |
| Remodeling of Epithelial Adherens Junctions | TUBB,TUBB4B |
| Systemic Lupus Erythematosus In T Cell Signaling Pathway | BCL6,GADD45A,RND3 |
| IL-7 Signaling Pathway | BCL6,MYC |
| Phosphatidylcholine Biosynthesis I | CHPT1 |
| Sirtuin Signaling Pathway | GADD45A,MYC,MYCN |
| Leucine Degradation I | BCAT2 |
| Gαs Signaling | CNR1,PTH1R |
| GP6 Signaling Pathway | COL1A2,COL23A1 |
| Gαi Signaling | CNR1,RGS10 |
| 14-3-3-mediated Signaling | TUBB,TUBB4B |
| Isoleucine Degradation I | BCAT2 |
| Choline Biosynthesis III | CHPT1 |
| Phagosome Maturation | TUBB,TUBB4B |
| Parkinson's Signaling | SNCAIP |
| Endocannabinoid Cancer Inhibition Pathway | CNR1,MYC |
| Epithelial Adherens Junction Signaling | TUBB,TUBB4B |
| Valine Degradation I | BCAT2 |
| GADD45 Signaling | GADD45A |
| Glioblastoma Multiforme Signaling | MYC,RND3 |
| Polyamine Regulation in Colon Cancer | MYC |
| Sertoli Cell-Sertoli Cell Junction Signaling | TUBB,TUBB4B |
| Hepatic Fibrosis / Hepatic Stellate Cell Activation | COL1A2,COL23A1 |
| ILK Signaling | MYC,RND3 |
| Estrogen-mediated S-phase Entry | MYC |
| Apelin Liver Signaling Pathway | COL1A2 |
|  | |
| **DD vs DC__DD vs DDC** |  |
| **Pathways** | **Molecules** |
| TGF-β Signaling | TGFB2 |
| Superpathway of Citrulline Metabolism | ASS1,LOC102724788/PRODH |
| Apelin Cardiac Fibroblast Signaling Pathway | CCN2,TGFB2 |
| Histamine Biosynthesis | HDC |
| Asparagine Biosynthesis I | ASNS |
| Taurine Biosynthesis | CDO1 |
| Inhibition of Matrix Metalloproteases | A2M,MMP28 |
| Methylglyoxal Degradation I | HAGHL |
| Proline Degradation | LOC102724788/PRODH |
| L-cysteine Degradation I | CDO1 |
| Phospholipases | PLCH1,PLD5 |
| Citrulline-Nitric Oxide Cycle | ASS1 |
| Arginine Biosynthesis IV | ASS1 |
| Urea Cycle | ASS1 |
| Glycoaminoglycan-protein Linkage Region Biosynthesis | XYLT1 |
| Hepatic Fibrosis / Hepatic Stellate Cell Activation | A2M,CCN2,TGFB2 |
| Growth Hormone Signaling | A2M,SOCS2 |
| Citrulline Biosynthesis | LOC102724788/PRODH |
| Role of NFAT in Cardiac Hypertrophy | CACNA1H,PLCH1,TGFB2 |
| Death Receptor Signaling | PARP8,TNFRSF21 |
| Guanosine Nucleotides Degradation III | NT5M |
| Urate Biosynthesis/Inosine 5'-phosphate Degradation | NT5M |
| UVA-Induced MAPK Signaling | PARP8,PLCH1 |
| Choline Biosynthesis III | PLD5 |
| Antioxidant Action of Vitamin C | PLCH1,PLD5 |
| Adenosine Nucleotides Degradation II | NT5M |
| IGF-1 Signaling | CCN2,SOCS2 |
| Pancreatic Adenocarcinoma Signaling | PLD5,TGFB2 |
| GPCR-Mediated Nutrient Sensing in Enteroendocrine Cells | CACNA1H,PLCH1 |
| Purine Nucleotides Degradation II (Aerobic) | NT5M |
| p38 MAPK Signaling | MKNK2,TGFB2 |
| NAD Salvage Pathway II | NT5M |
| Endocannabinoid Neuronal Synapse Pathway | CACNA1H,PLCH1 |
| Adipogenesis pathway | DGKD,SREBF1 |
| STAT3 Pathway | SOCS2,TGFB2 |
| D-myo-inositol (1,4,5)-Trisphosphate Biosynthesis | PLCH1 |
| Type II Diabetes Mellitus Signaling | CACNA1H,SOCS2 |
| Antiproliferative Role of TOB in T Cell Signaling | TGFB2 |
|  | |
| **All conditions** |  |
| **Pathways** | **Molecules** |
| SPINK1 General Cancer Pathway | MT1A,MT1G,MT1X,MT2A,MT3 |
| Ascorbate Recycling (Cytosolic) | GLRX |
| Glutathione Redox Reactions II | GLRX |
| VDR/RXR Activation | IGFBP3,SPP1 |
| Chemokine Signaling | CCL2,CXCR4 |
| Sucrose Degradation V (Mammalian) | ALDOC |
| Atherosclerosis Signaling | CCL2,CXCR4 |
| Glucocorticoid Receptor Signaling | CCL2,FKBP5,TSC22D3 |
| Aryl Hydrocarbon Receptor Signaling | ALDH1L1,CCND3 |
| Vitamin-C Transport | GLRX |
| Differential Regulation of Cytokine Production in Macrophages and T Helper Cells by IL-17A and IL-17F | CCL2 |
| GADD45 Signaling | CCND3 |
| Granulocyte Adhesion and Diapedesis | CCL2,CXCR4 |
| Agranulocyte Adhesion and Diapedesis | CCL2,CXCR4 |
| Differential Regulation of Cytokine Production in Intestinal Epithelial Cells by IL-17A and IL-17F | CCL2 |
| Hepatic Fibrosis / Hepatic Stellate Cell Activation | CCL2,IGFBP3 |
| Glycolysis I | ALDOC |
| Gluconeogenesis I | ALDOC |
